# Supplementary material for: Using the Situated Learning-Guided Educational Framework to Teach Anatomy of the Infratemporal Fossa and Retromandibular Region
Source: MedEdPORTAL. 2025 Oct 3;21:11550. doi: 10.15766/mep_2374-8265.11550 (PMC12491565; doi:10.15766/mep_2374-8265.11550)
Supplement: Supplementary file 1 — Infratemporal Fossa Module (Instructor).pptxRetromandibular Region Module (Instructor).pptxInfratemporal Fossa Module (Student).pptxRetromandibular Region Module (Student).pptxPretest.docxPosttest.docxSurvey - Infratemporal Fossa.docxSurvey - Retromandibular Region.docx [file mep_2374-8265.11550-s001.zip › D. Retromandibular Region Module (Student).pptx]

## Slide 1
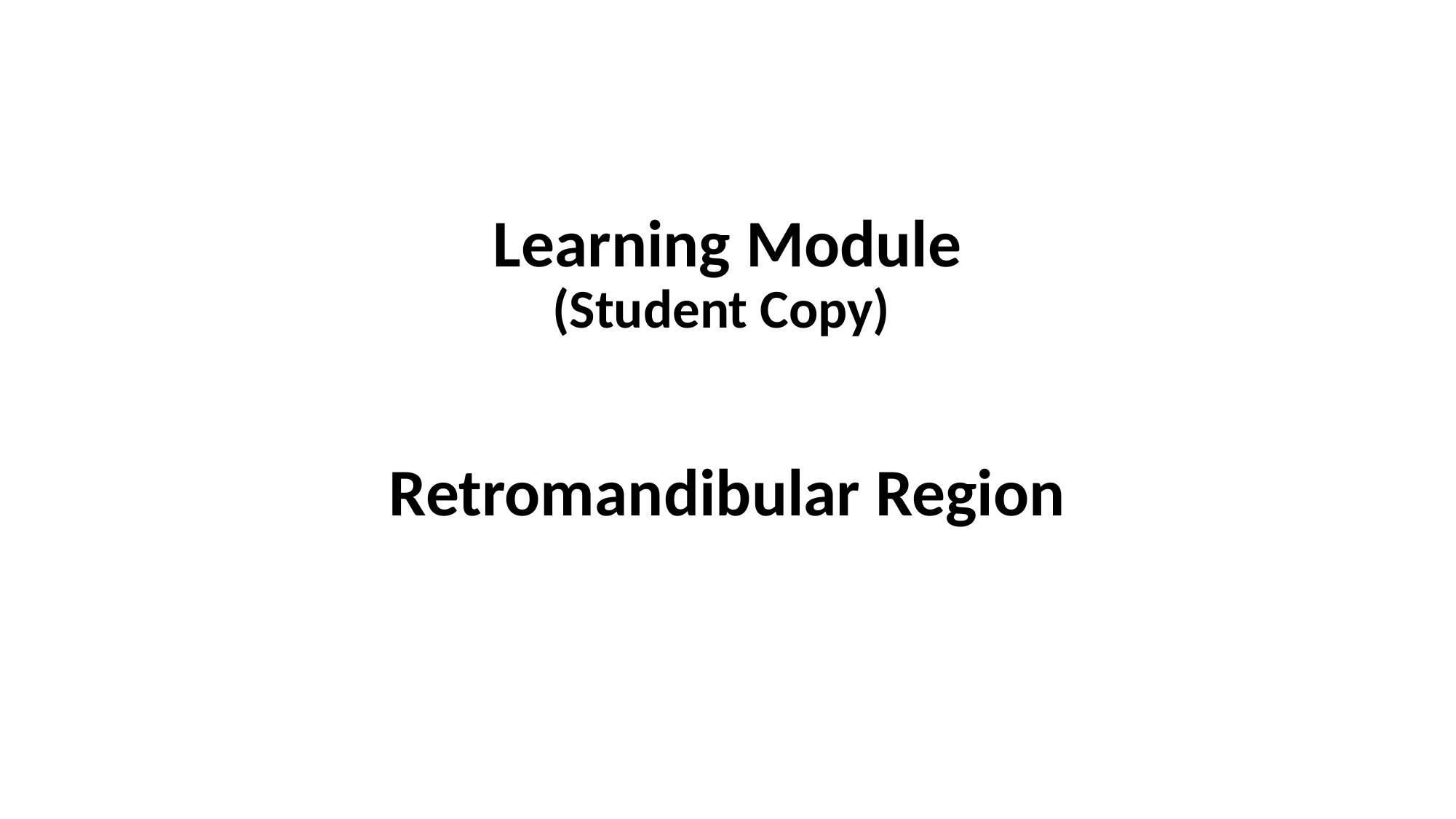

Learning Module
(Student Copy)
Retromandibular Region

## Slide 2
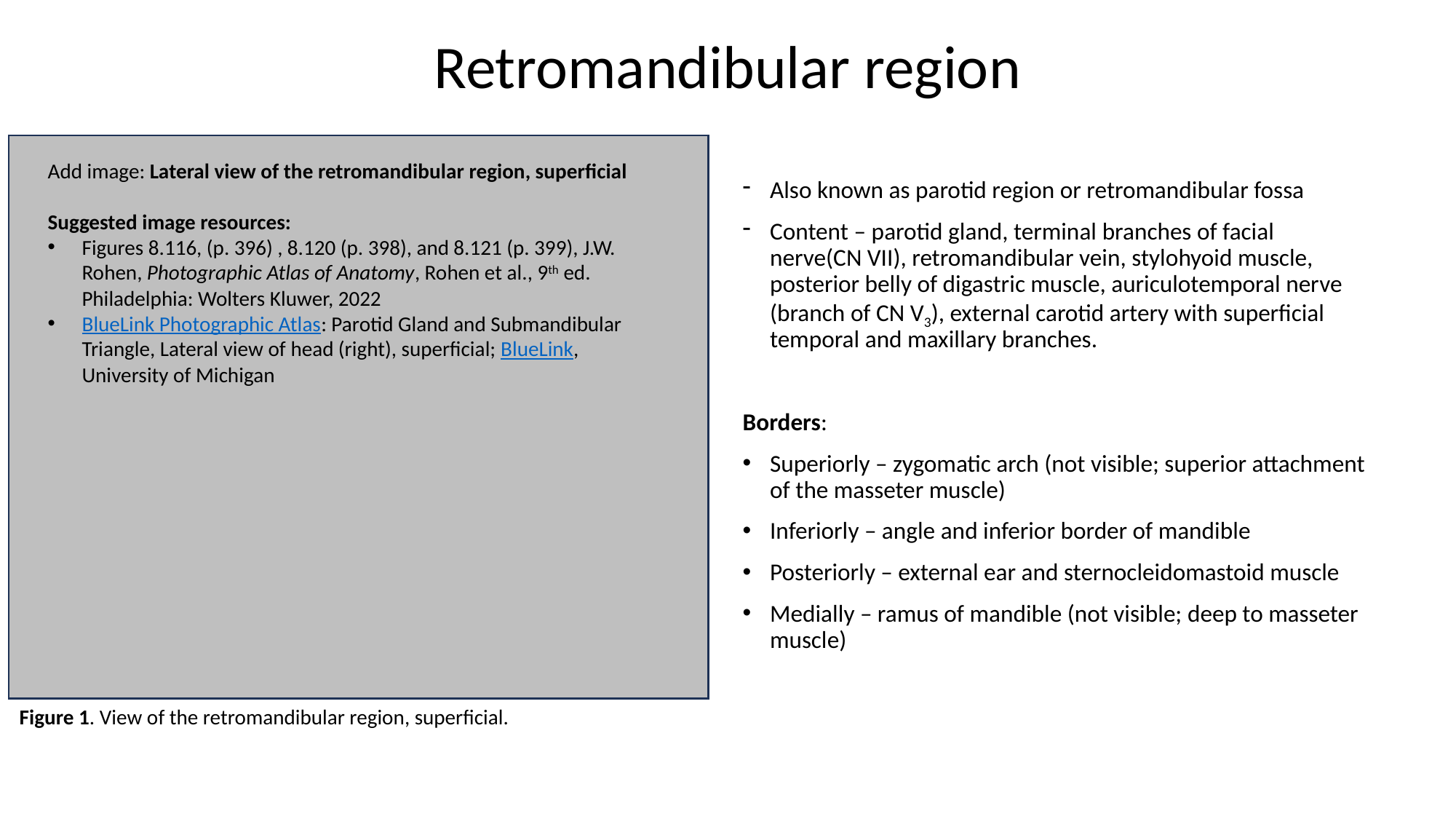

# Retromandibular region
Add image: Lateral view of the retromandibular region, superficial
Suggested image resources:
Figures 8.116, (p. 396) , 8.120 (p. 398), and 8.121 (p. 399), J.W. Rohen, Photographic Atlas of Anatomy, Rohen et al., 9th ed. Philadelphia: Wolters Kluwer, 2022
BlueLink Photographic Atlas: Parotid Gland and Submandibular Triangle, Lateral view of head (right), superficial; BlueLink, University of Michigan
Also known as parotid region or retromandibular fossa
Content – parotid gland, terminal branches of facial nerve(CN VII), retromandibular vein, stylohyoid muscle, posterior belly of digastric muscle, auriculotemporal nerve (branch of CN V3), external carotid artery with superficial temporal and maxillary branches.
Borders:
Superiorly – zygomatic arch (not visible; superior attachment of the masseter muscle)
Inferiorly – angle and inferior border of mandible
Posteriorly – external ear and sternocleidomastoid muscle
Medially – ramus of mandible (not visible; deep to masseter muscle)
Figure 1. View of the retromandibular region, superficial.

## Slide 3
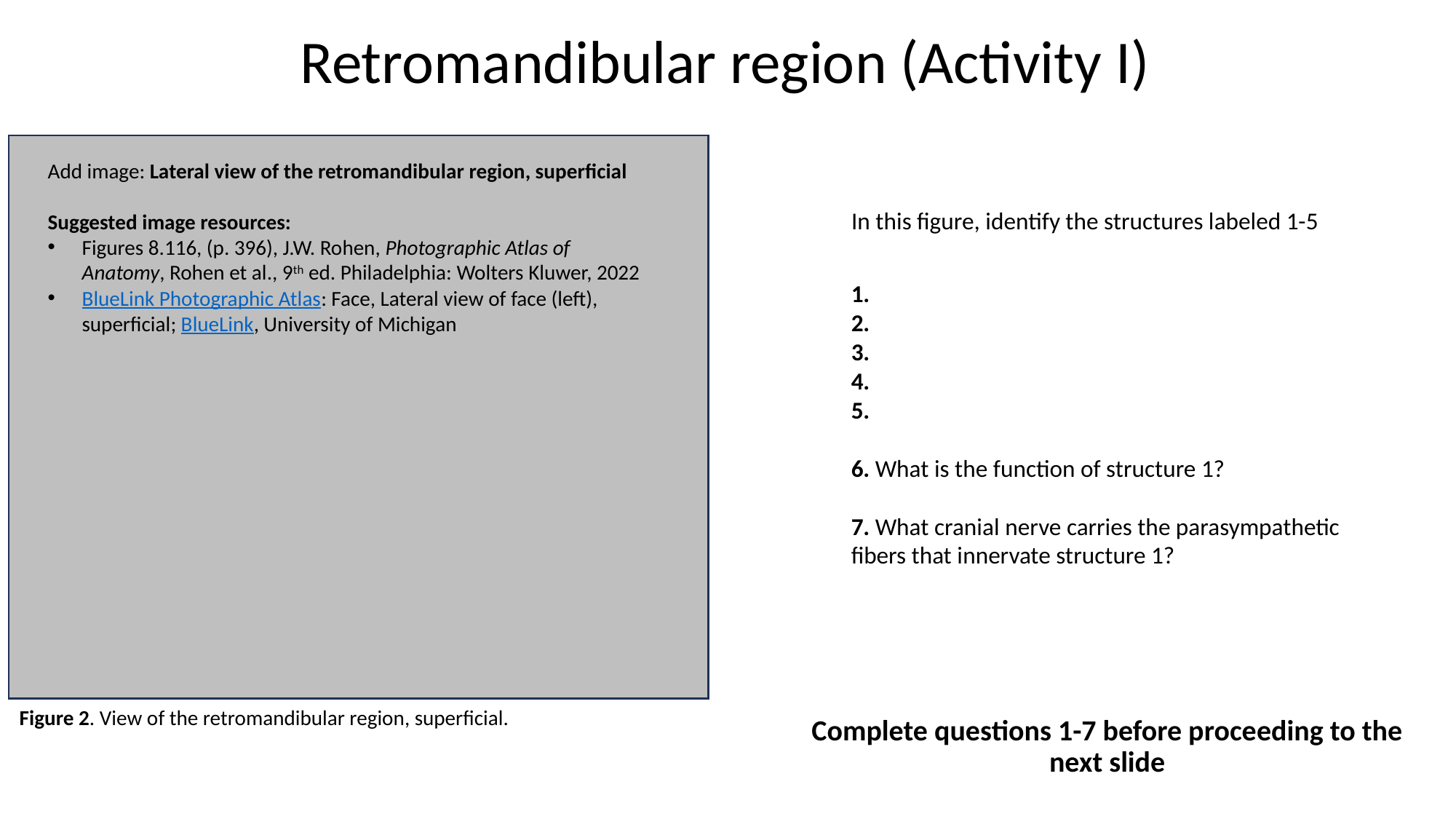

Retromandibular region (Activity I)
Add image: Lateral view of the retromandibular region, superficial
Suggested image resources:
Figures 8.116, (p. 396), J.W. Rohen, Photographic Atlas of Anatomy, Rohen et al., 9th ed. Philadelphia: Wolters Kluwer, 2022
BlueLink Photographic Atlas: Face, Lateral view of face (left), superficial; BlueLink, University of Michigan
In this figure, identify the structures labeled 1-5
1.
2.
3.
4.
5.
6. What is the function of structure 1?
7. What cranial nerve carries the parasympathetic fibers that innervate structure 1?
# Complete questions 1-7 before proceeding to the next slide
Figure 2. View of the retromandibular region, superficial.

## Slide 4
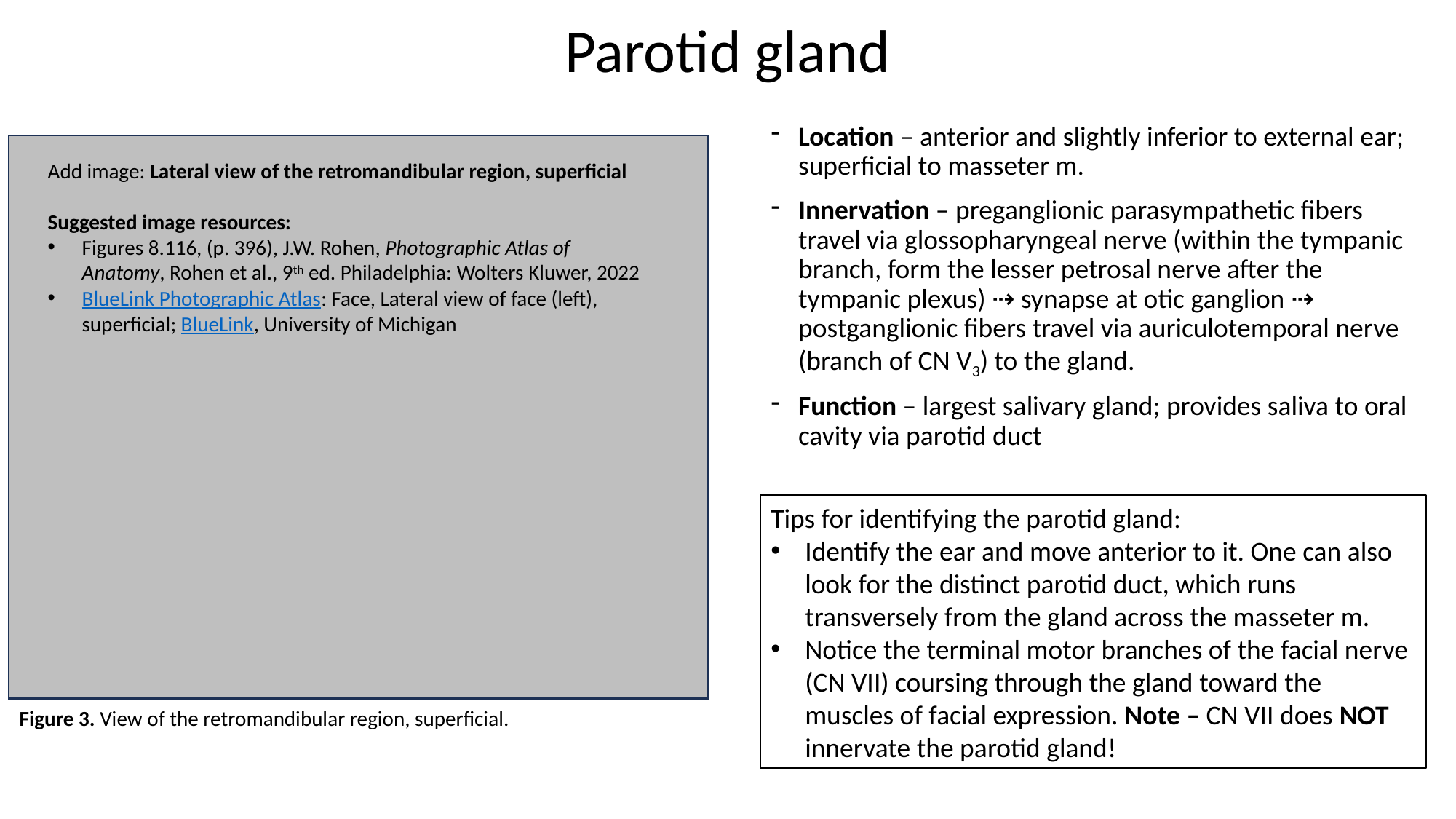

# Parotid gland
Location – anterior and slightly inferior to external ear; superficial to masseter m.
Innervation – preganglionic parasympathetic fibers travel via glossopharyngeal nerve (within the tympanic branch, form the lesser petrosal nerve after the tympanic plexus) ⇢ synapse at otic ganglion ⇢ postganglionic fibers travel via auriculotemporal nerve (branch of CN V3) to the gland.
Function – largest salivary gland; provides saliva to oral cavity via parotid duct
Add image: Lateral view of the retromandibular region, superficial
Suggested image resources:
Figures 8.116, (p. 396), J.W. Rohen, Photographic Atlas of Anatomy, Rohen et al., 9th ed. Philadelphia: Wolters Kluwer, 2022
BlueLink Photographic Atlas: Face, Lateral view of face (left), superficial; BlueLink, University of Michigan
Tips for identifying the parotid gland:
Identify the ear and move anterior to it. One can also look for the distinct parotid duct, which runs transversely from the gland across the masseter m.
Notice the terminal motor branches of the facial nerve (CN VII) coursing through the gland toward the muscles of facial expression. Note – CN VII does NOT innervate the parotid gland!
Figure 3. View of the retromandibular region, superficial.

## Slide 5
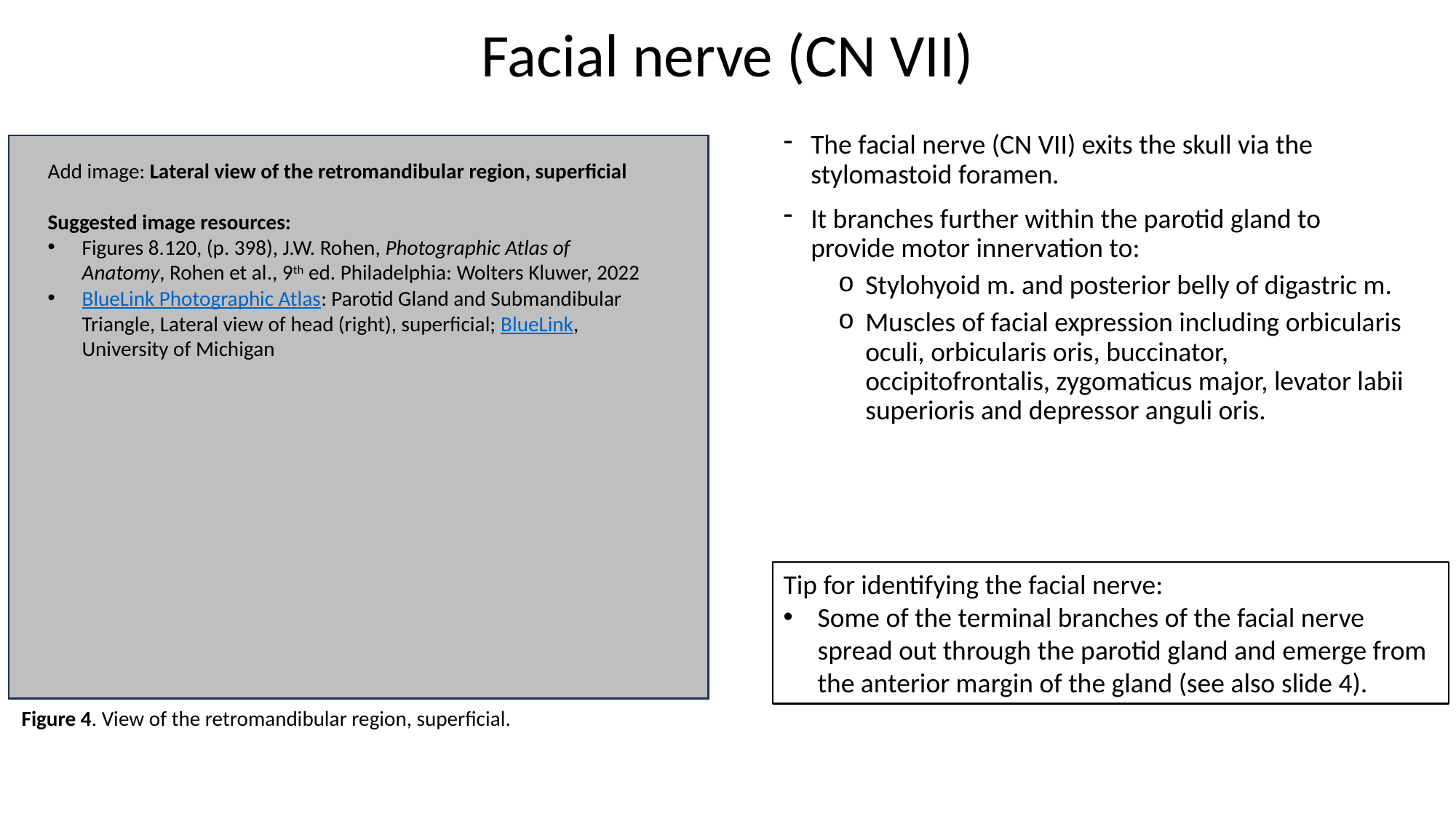

# Facial nerve (CN VII)
The facial nerve (CN VII) exits the skull via the stylomastoid foramen.
It branches further within the parotid gland to provide motor innervation to:
Stylohyoid m. and posterior belly of digastric m.
Muscles of facial expression including orbicularis oculi, orbicularis oris, buccinator, occipitofrontalis, zygomaticus major, levator labii superioris and depressor anguli oris.
Add image: Lateral view of the retromandibular region, superficial
Suggested image resources:
Figures 8.120, (p. 398), J.W. Rohen, Photographic Atlas of Anatomy, Rohen et al., 9th ed. Philadelphia: Wolters Kluwer, 2022
BlueLink Photographic Atlas: Parotid Gland and Submandibular Triangle, Lateral view of head (right), superficial; BlueLink, University of Michigan
Tip for identifying the facial nerve:
Some of the terminal branches of the facial nerve spread out through the parotid gland and emerge from the anterior margin of the gland (see also slide 4).
Figure 4. View of the retromandibular region, superficial.

## Slide 6
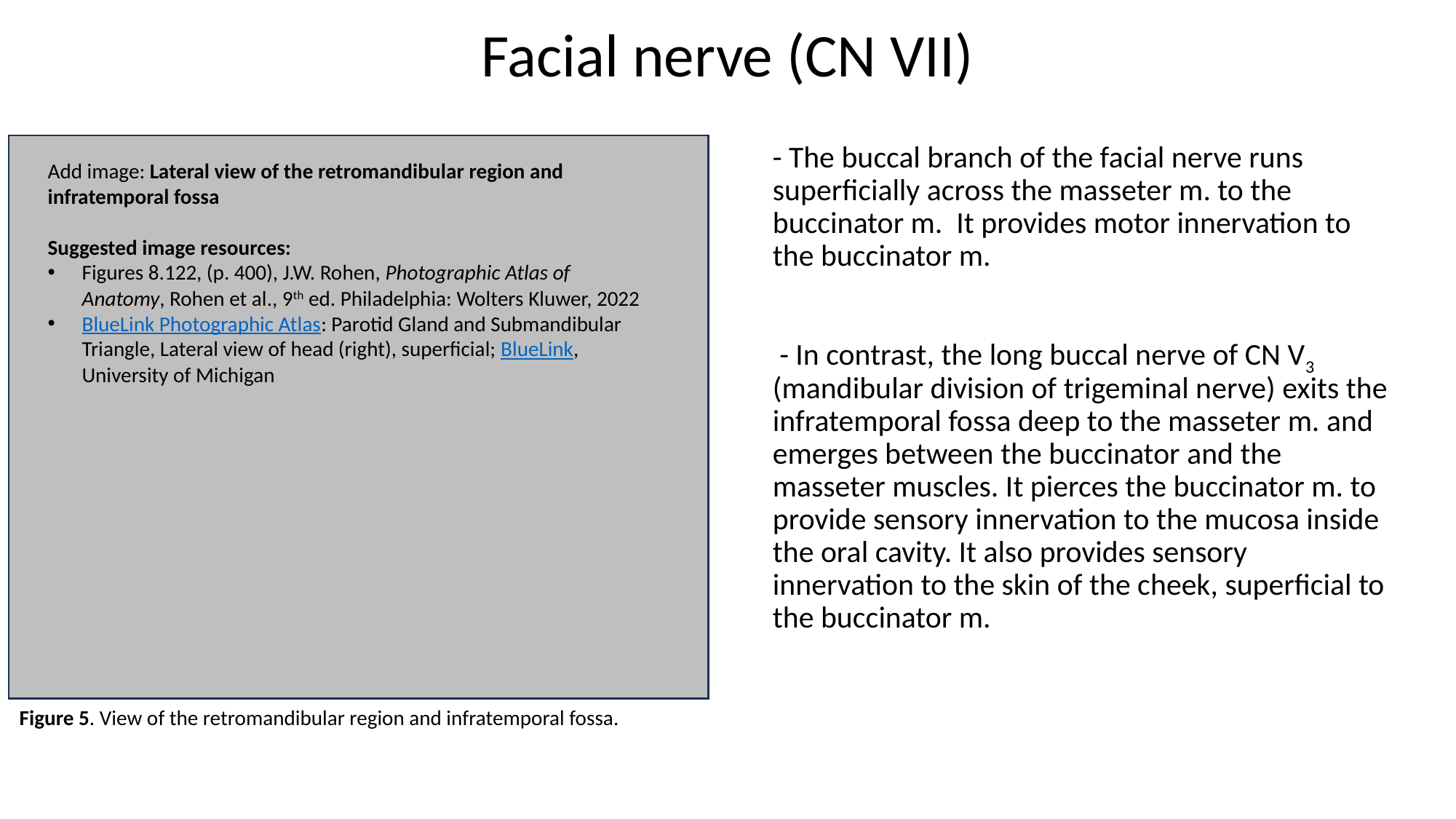

Facial nerve (CN VII)
- The buccal branch of the facial nerve runs superficially across the masseter m. to the buccinator m. It provides motor innervation to the buccinator m.
 - In contrast, the long buccal nerve of CN V3 (mandibular division of trigeminal nerve) exits the infratemporal fossa deep to the masseter m. and emerges between the buccinator and the masseter muscles. It pierces the buccinator m. to provide sensory innervation to the mucosa inside the oral cavity. It also provides sensory innervation to the skin of the cheek, superficial to the buccinator m.
Add image: Lateral view of the retromandibular region and infratemporal fossa
Suggested image resources:
Figures 8.122, (p. 400), J.W. Rohen, Photographic Atlas of Anatomy, Rohen et al., 9th ed. Philadelphia: Wolters Kluwer, 2022
BlueLink Photographic Atlas: Parotid Gland and Submandibular Triangle, Lateral view of head (right), superficial; BlueLink, University of Michigan
Figure 5. View of the retromandibular region and infratemporal fossa.

## Slide 7
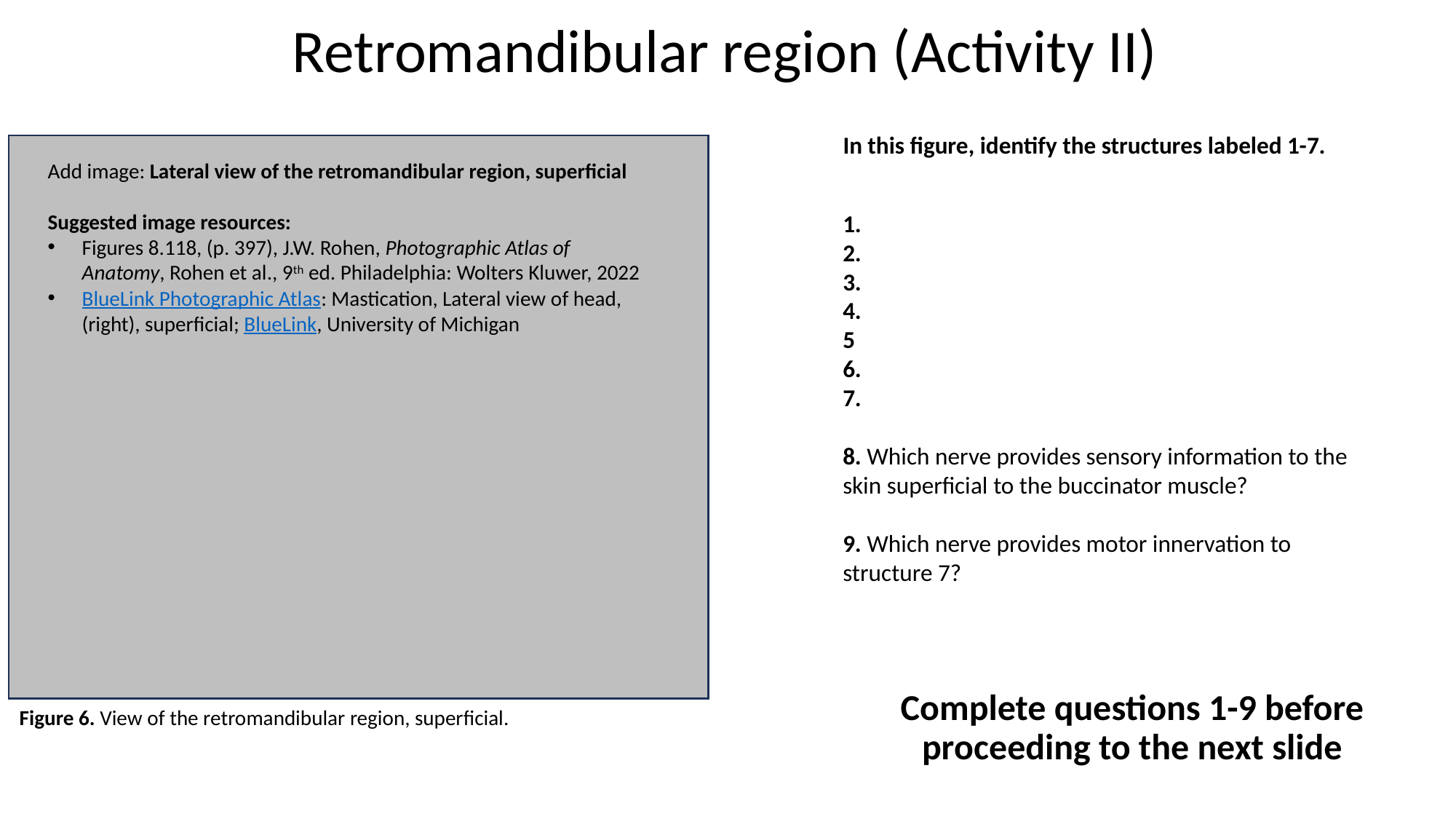

Retromandibular region (Activity II)
In this figure, identify the structures labeled 1-7.
Add image: Lateral view of the retromandibular region, superficial
Suggested image resources:
Figures 8.118, (p. 397), J.W. Rohen, Photographic Atlas of Anatomy, Rohen et al., 9th ed. Philadelphia: Wolters Kluwer, 2022
BlueLink Photographic Atlas: Mastication, Lateral view of head, (right), superficial; BlueLink, University of Michigan
1.
2.
3.
4.
5
6.
7.
8. Which nerve provides sensory information to the skin superficial to the buccinator muscle?
9. Which nerve provides motor innervation to structure 7?
Complete questions 1-9 before proceeding to the next slide
Figure 6. View of the retromandibular region, superficial.

## Slide 8
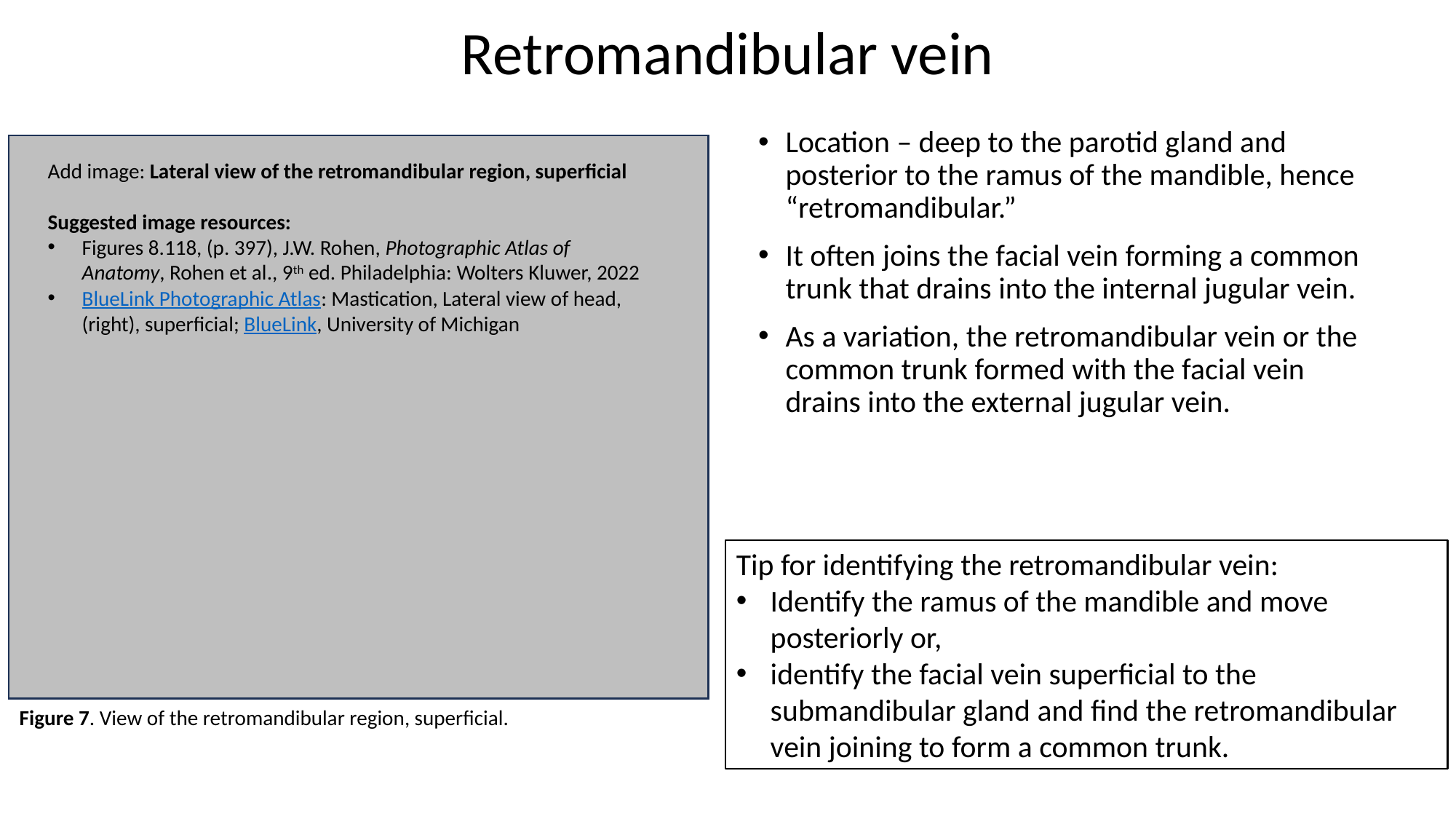

# Retromandibular vein
Location – deep to the parotid gland and posterior to the ramus of the mandible, hence “retromandibular.”
It often joins the facial vein forming a common trunk that drains into the internal jugular vein.
As a variation, the retromandibular vein or the common trunk formed with the facial vein drains into the external jugular vein.
Add image: Lateral view of the retromandibular region, superficial
Suggested image resources:
Figures 8.118, (p. 397), J.W. Rohen, Photographic Atlas of Anatomy, Rohen et al., 9th ed. Philadelphia: Wolters Kluwer, 2022
BlueLink Photographic Atlas: Mastication, Lateral view of head, (right), superficial; BlueLink, University of Michigan
Tip for identifying the retromandibular vein:
Identify the ramus of the mandible and move posteriorly or,
identify the facial vein superficial to the submandibular gland and find the retromandibular vein joining to form a common trunk.
Figure 7. View of the retromandibular region, superficial.

## Slide 9
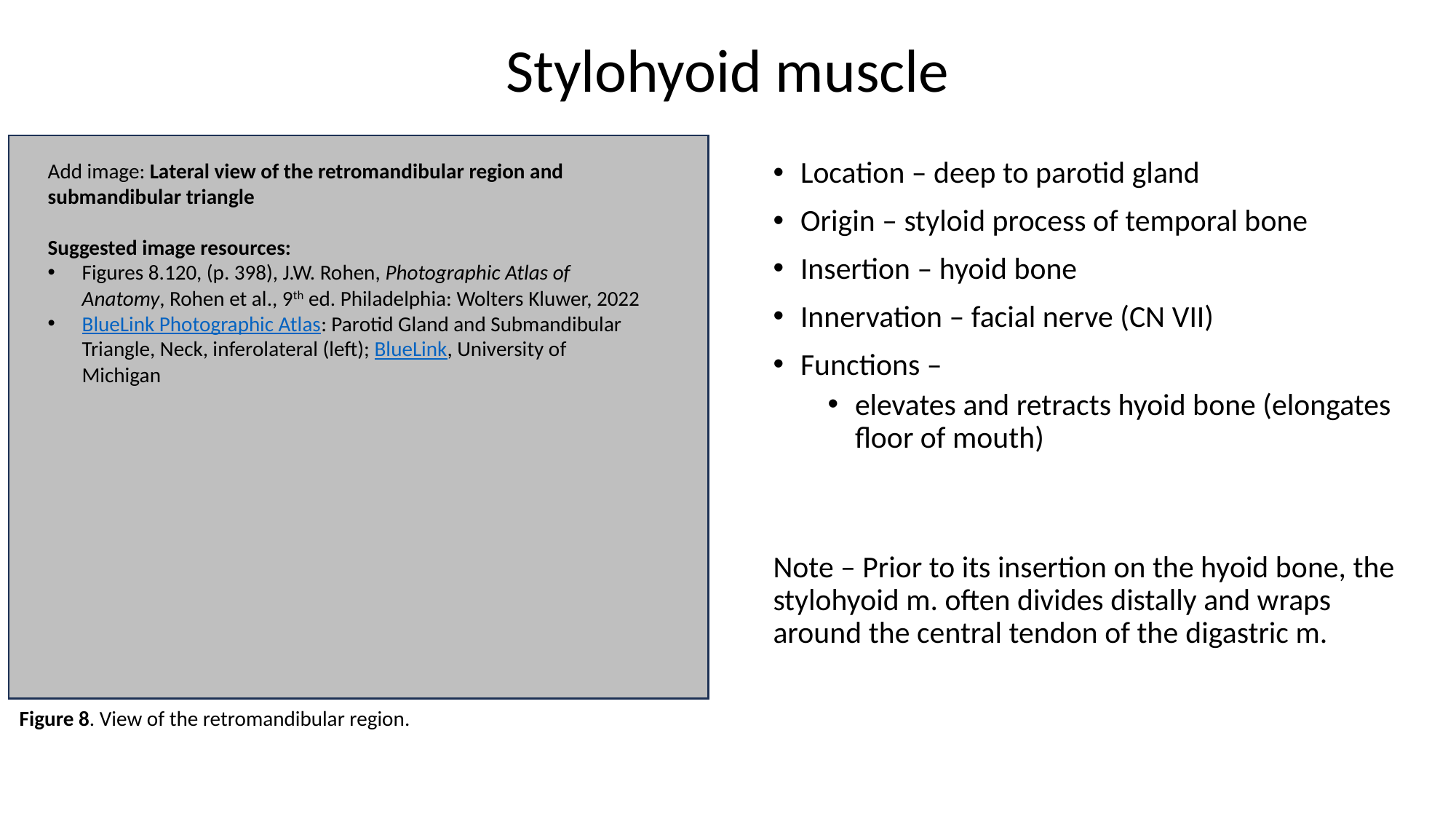

# Stylohyoid muscle
Location – deep to parotid gland
Origin – styloid process of temporal bone
Insertion – hyoid bone
Innervation – facial nerve (CN VII)
Functions –
elevates and retracts hyoid bone (elongates floor of mouth)
Note – Prior to its insertion on the hyoid bone, the stylohyoid m. often divides distally and wraps around the central tendon of the digastric m.
Add image: Lateral view of the retromandibular region and submandibular triangle
Suggested image resources:
Figures 8.120, (p. 398), J.W. Rohen, Photographic Atlas of Anatomy, Rohen et al., 9th ed. Philadelphia: Wolters Kluwer, 2022
BlueLink Photographic Atlas: Parotid Gland and Submandibular Triangle, Neck, inferolateral (left); BlueLink, University of Michigan
Figure 8. View of the retromandibular region.

## Slide 10
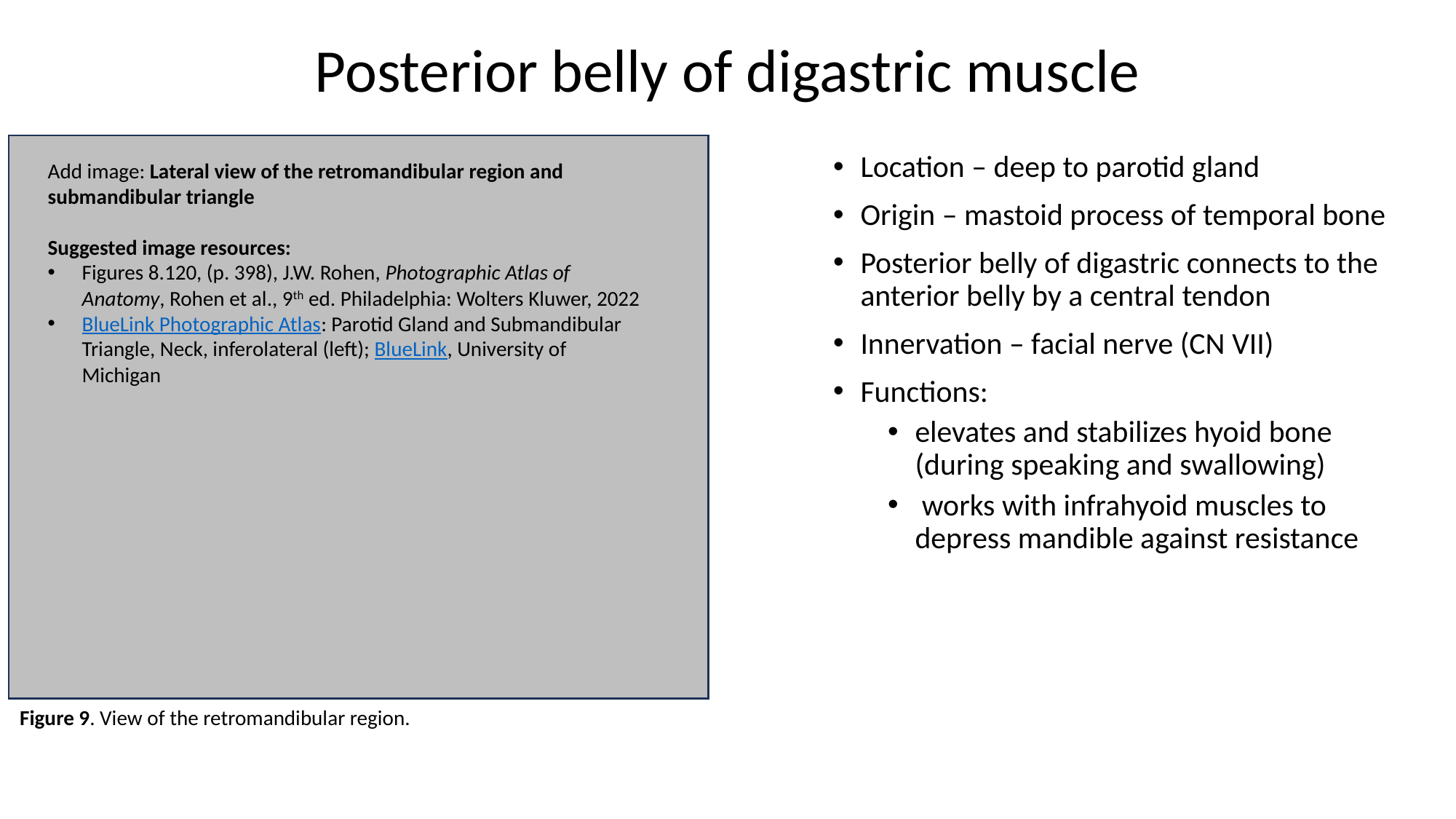

# Posterior belly of digastric muscle
Location – deep to parotid gland
Origin – mastoid process of temporal bone
Posterior belly of digastric connects to the anterior belly by a central tendon
Innervation – facial nerve (CN VII)
Functions:
elevates and stabilizes hyoid bone (during speaking and swallowing)
 works with infrahyoid muscles to depress mandible against resistance
Add image: Lateral view of the retromandibular region and submandibular triangle
Suggested image resources:
Figures 8.120, (p. 398), J.W. Rohen, Photographic Atlas of Anatomy, Rohen et al., 9th ed. Philadelphia: Wolters Kluwer, 2022
BlueLink Photographic Atlas: Parotid Gland and Submandibular Triangle, Neck, inferolateral (left); BlueLink, University of Michigan
Figure 9. View of the retromandibular region.

## Slide 11
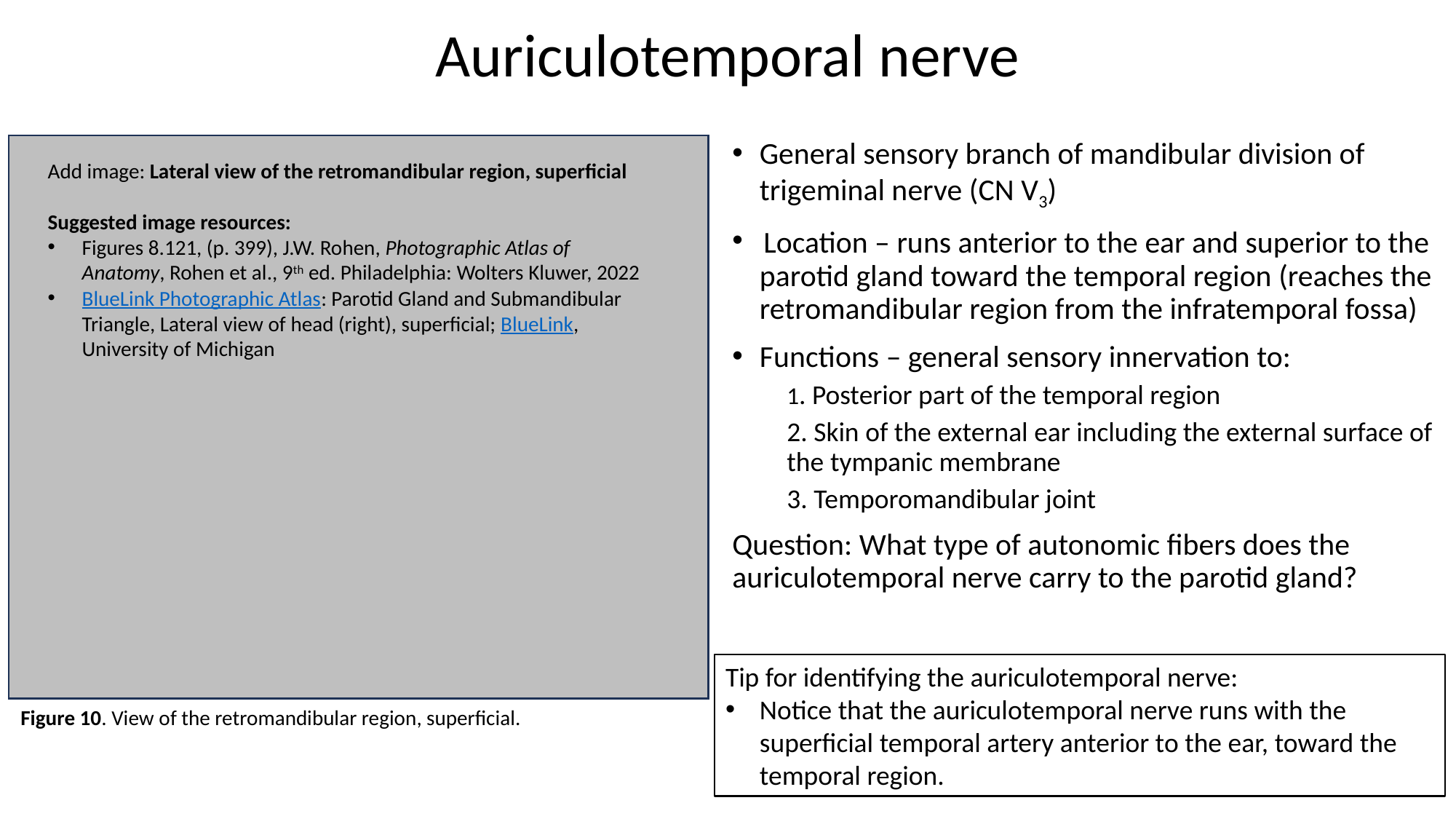

Auriculotemporal nerve
General sensory branch of mandibular division of trigeminal nerve (CN V3)
 Location – runs anterior to the ear and superior to the parotid gland toward the temporal region (reaches the retromandibular region from the infratemporal fossa)
Functions – general sensory innervation to:
1. Posterior part of the temporal region
2. Skin of the external ear including the external surface of the tympanic membrane
3. Temporomandibular joint
Question: What type of autonomic fibers does the auriculotemporal nerve carry to the parotid gland?
Add image: Lateral view of the retromandibular region, superficial
Suggested image resources:
Figures 8.121, (p. 399), J.W. Rohen, Photographic Atlas of Anatomy, Rohen et al., 9th ed. Philadelphia: Wolters Kluwer, 2022
BlueLink Photographic Atlas: Parotid Gland and Submandibular Triangle, Lateral view of head (right), superficial; BlueLink, University of Michigan
Tip for identifying the auriculotemporal nerve:
Notice that the auriculotemporal nerve runs with the superficial temporal artery anterior to the ear, toward the temporal region.
Figure 10. View of the retromandibular region, superficial.

## Slide 12
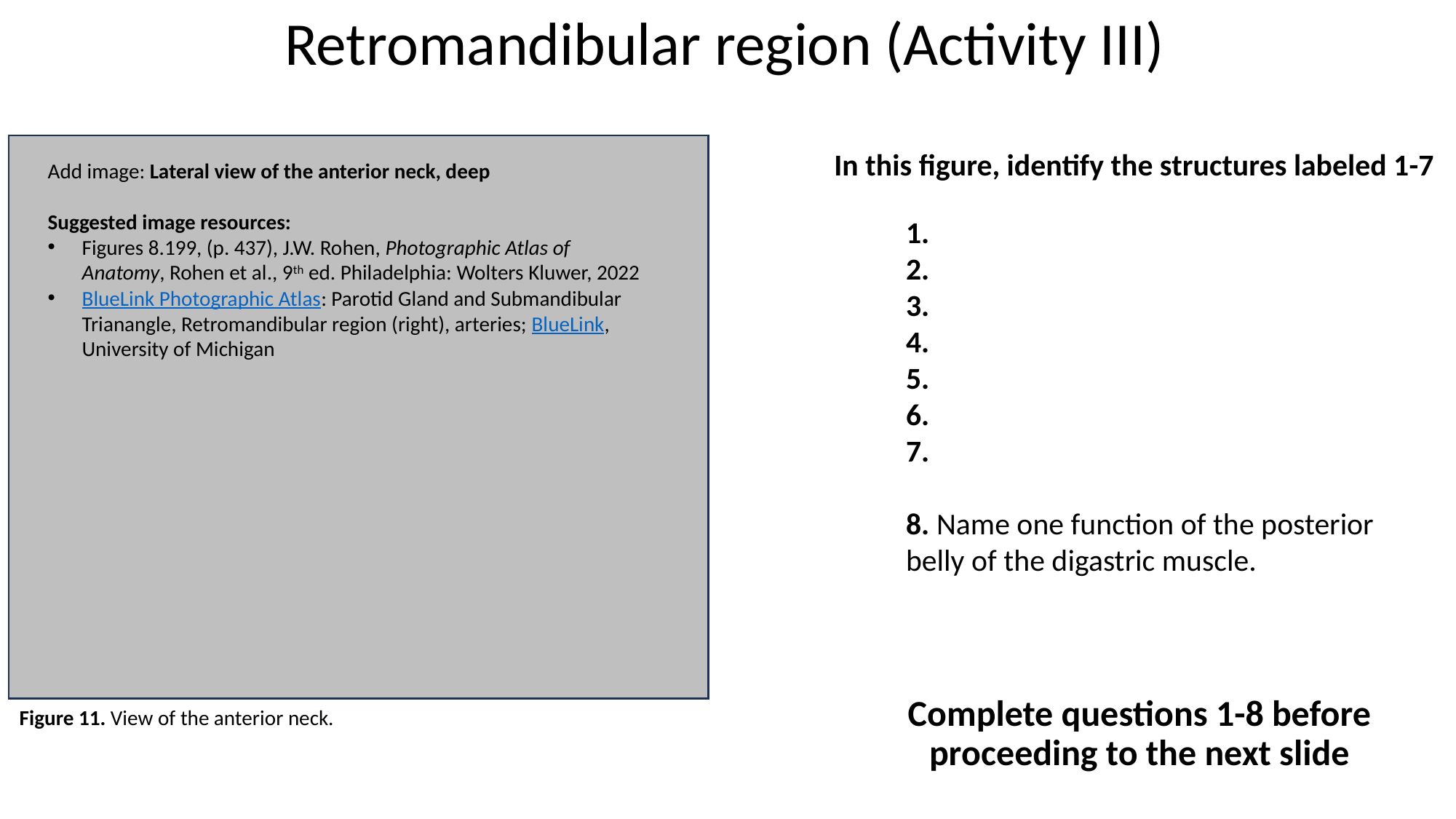

Retromandibular region (Activity III)
In this figure, identify the structures labeled 1-7
Add image: Lateral view of the anterior neck, deep
Suggested image resources:
Figures 8.199, (p. 437), J.W. Rohen, Photographic Atlas of Anatomy, Rohen et al., 9th ed. Philadelphia: Wolters Kluwer, 2022
BlueLink Photographic Atlas: Parotid Gland and Submandibular Trianangle, Retromandibular region (right), arteries; BlueLink, University of Michigan
1.
2.
3.
4.
5.
6.
7.
8. Name one function of the posterior belly of the digastric muscle.
# Complete questions 1-8 before proceeding to the next slide
Figure 11. View of the anterior neck.

## Slide 13
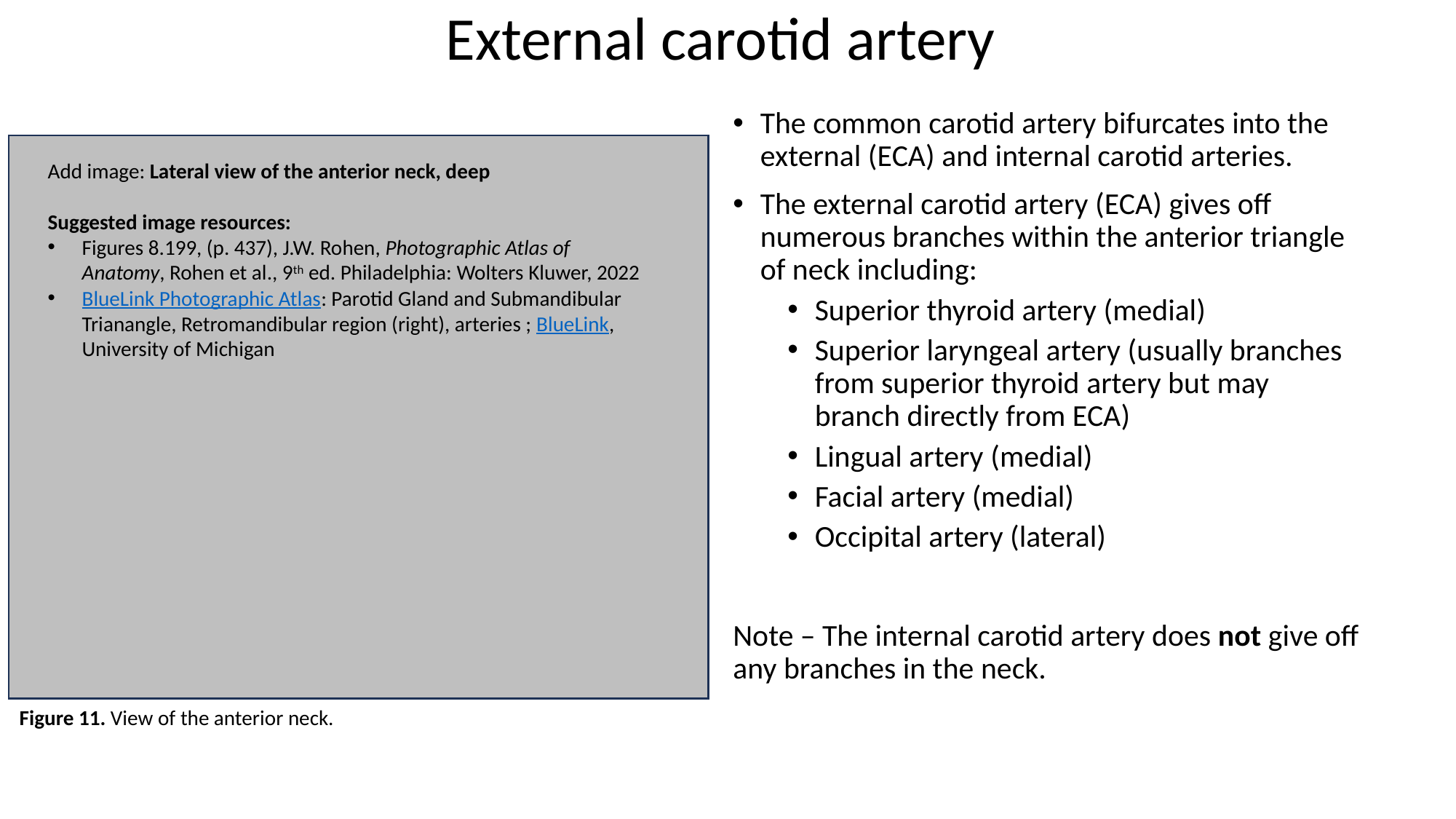

External carotid artery
The common carotid artery bifurcates into the external (ECA) and internal carotid arteries.
The external carotid artery (ECA) gives off numerous branches within the anterior triangle of neck including:
Superior thyroid artery (medial)
Superior laryngeal artery (usually branches from superior thyroid artery but may branch directly from ECA)
Lingual artery (medial)
Facial artery (medial)
Occipital artery (lateral)
Note – The internal carotid artery does not give off any branches in the neck.
Add image: Lateral view of the anterior neck, deep
Suggested image resources:
Figures 8.199, (p. 437), J.W. Rohen, Photographic Atlas of Anatomy, Rohen et al., 9th ed. Philadelphia: Wolters Kluwer, 2022
BlueLink Photographic Atlas: Parotid Gland and Submandibular Trianangle, Retromandibular region (right), arteries ; BlueLink, University of Michigan
Figure 11. View of the anterior neck.

## Slide 14
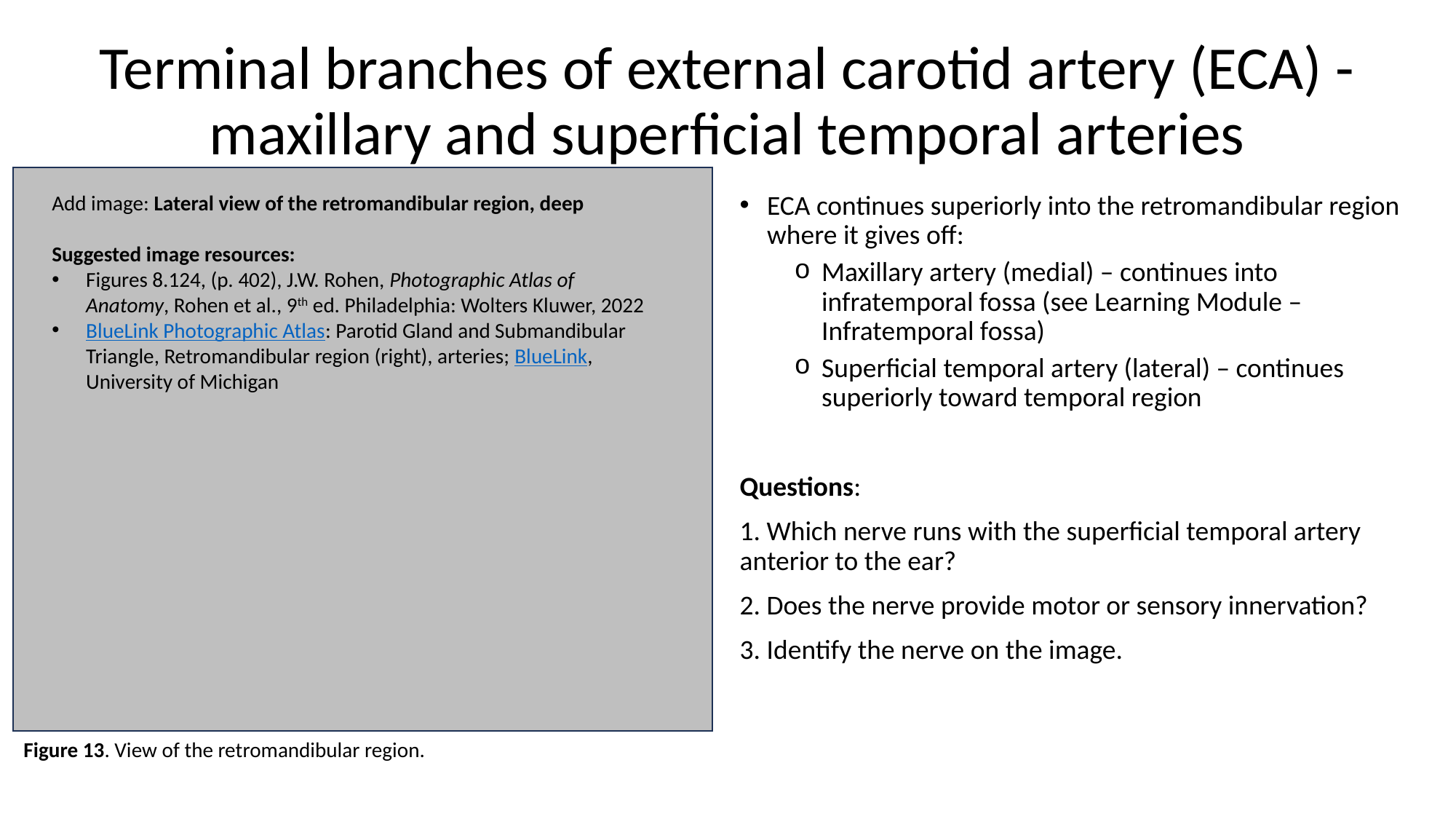

# Terminal branches of external carotid artery (ECA) - maxillary and superficial temporal arteries
Add image: Lateral view of the retromandibular region, deep
Suggested image resources:
Figures 8.124, (p. 402), J.W. Rohen, Photographic Atlas of Anatomy, Rohen et al., 9th ed. Philadelphia: Wolters Kluwer, 2022
BlueLink Photographic Atlas: Parotid Gland and Submandibular Triangle, Retromandibular region (right), arteries; BlueLink, University of Michigan
ECA continues superiorly into the retromandibular region where it gives off:
Maxillary artery (medial) – continues into infratemporal fossa (see Learning Module – Infratemporal fossa)
Superficial temporal artery (lateral) – continues superiorly toward temporal region
Questions:
1. Which nerve runs with the superficial temporal artery anterior to the ear?
2. Does the nerve provide motor or sensory innervation?
3. Identify the nerve on the image.
Figure 13. View of the retromandibular region.

## Slide 15
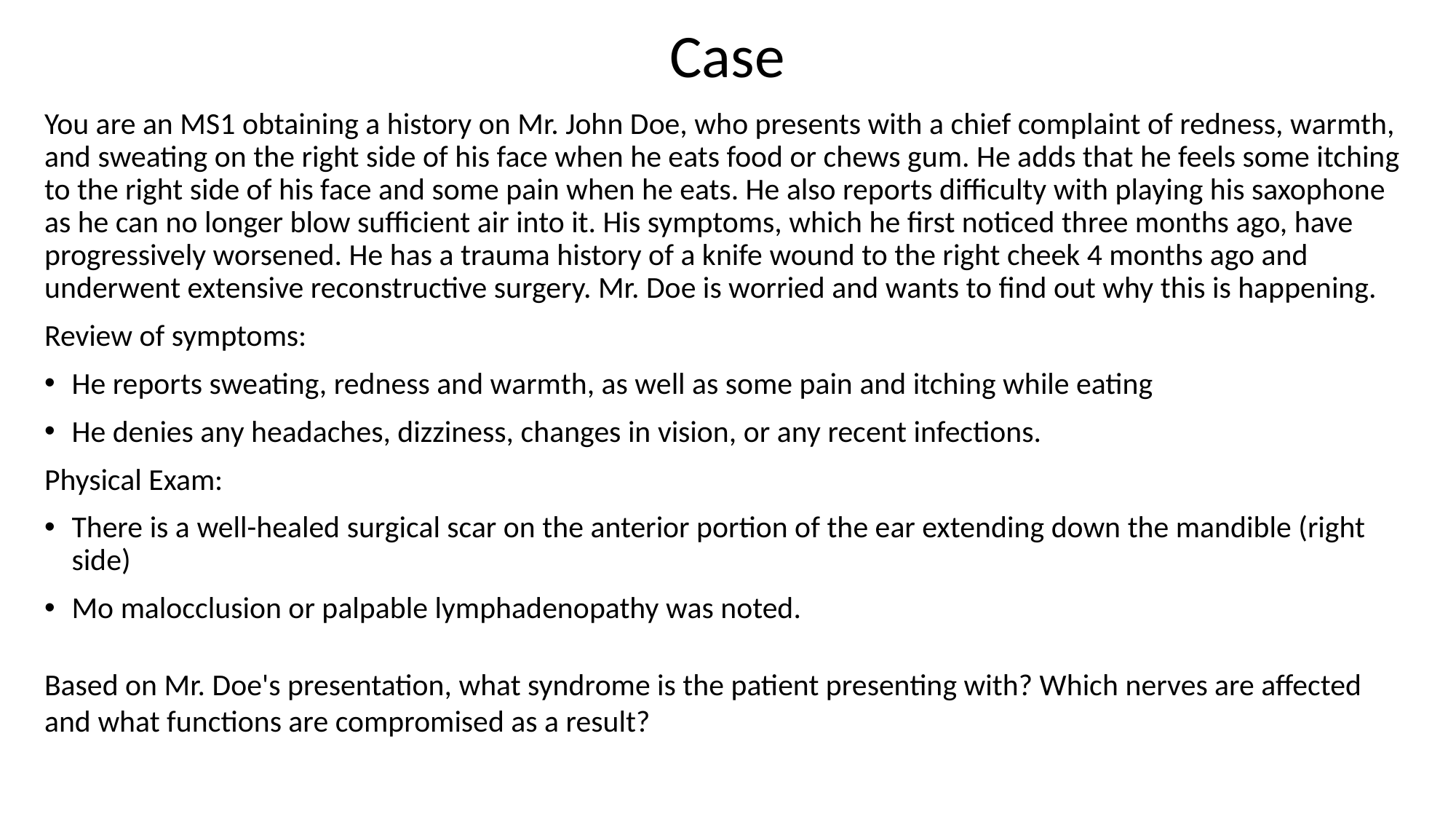

# Case
You are an MS1 obtaining a history on Mr. John Doe, who presents with a chief complaint of redness, warmth, and sweating on the right side of his face when he eats food or chews gum. He adds that he feels some itching to the right side of his face and some pain when he eats. He also reports difficulty with playing his saxophone as he can no longer blow sufficient air into it. His symptoms, which he first noticed three months ago, have progressively worsened. He has a trauma history of a knife wound to the right cheek 4 months ago and underwent extensive reconstructive surgery. Mr. Doe is worried and wants to find out why this is happening.
Review of symptoms:
He reports sweating, redness and warmth, as well as some pain and itching while eating
He denies any headaches, dizziness, changes in vision, or any recent infections.
Physical Exam:
There is a well-healed surgical scar on the anterior portion of the ear extending down the mandible (right side)
Mo malocclusion or palpable lymphadenopathy was noted.
Based on Mr. Doe's presentation, what syndrome is the patient presenting with? Which nerves are affected and what functions are compromised as a result?

## Slide 16
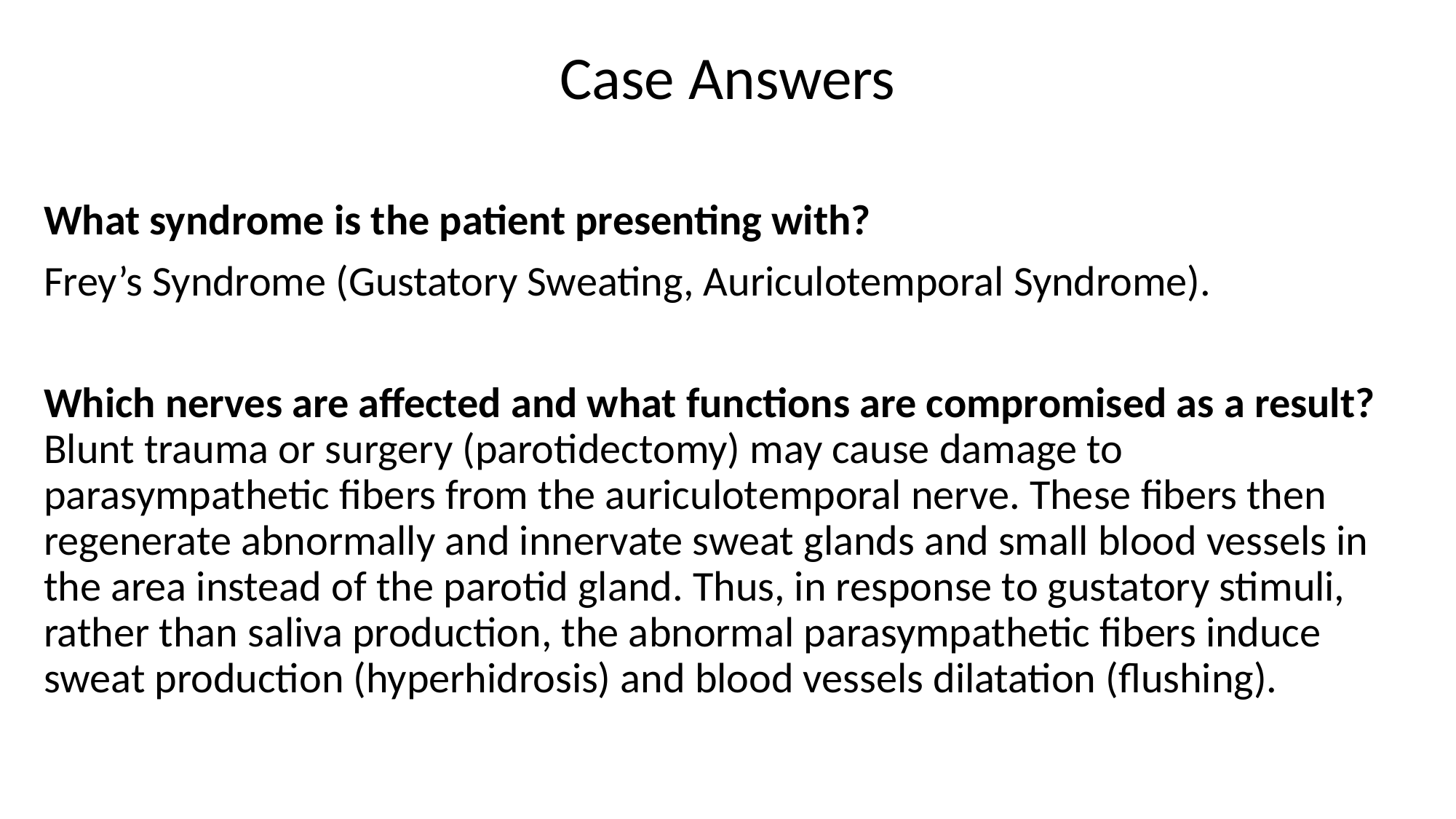

# Case Answers
What syndrome is the patient presenting with?
Frey’s Syndrome (Gustatory Sweating, Auriculotemporal Syndrome).
Which nerves are affected and what functions are compromised as a result? Blunt trauma or surgery (parotidectomy) may cause damage to parasympathetic fibers from the auriculotemporal nerve. These fibers then regenerate abnormally and innervate sweat glands and small blood vessels in the area instead of the parotid gland. Thus, in response to gustatory stimuli, rather than saliva production, the abnormal parasympathetic fibers induce sweat production (hyperhidrosis) and blood vessels dilatation (flushing).

## Slide 17
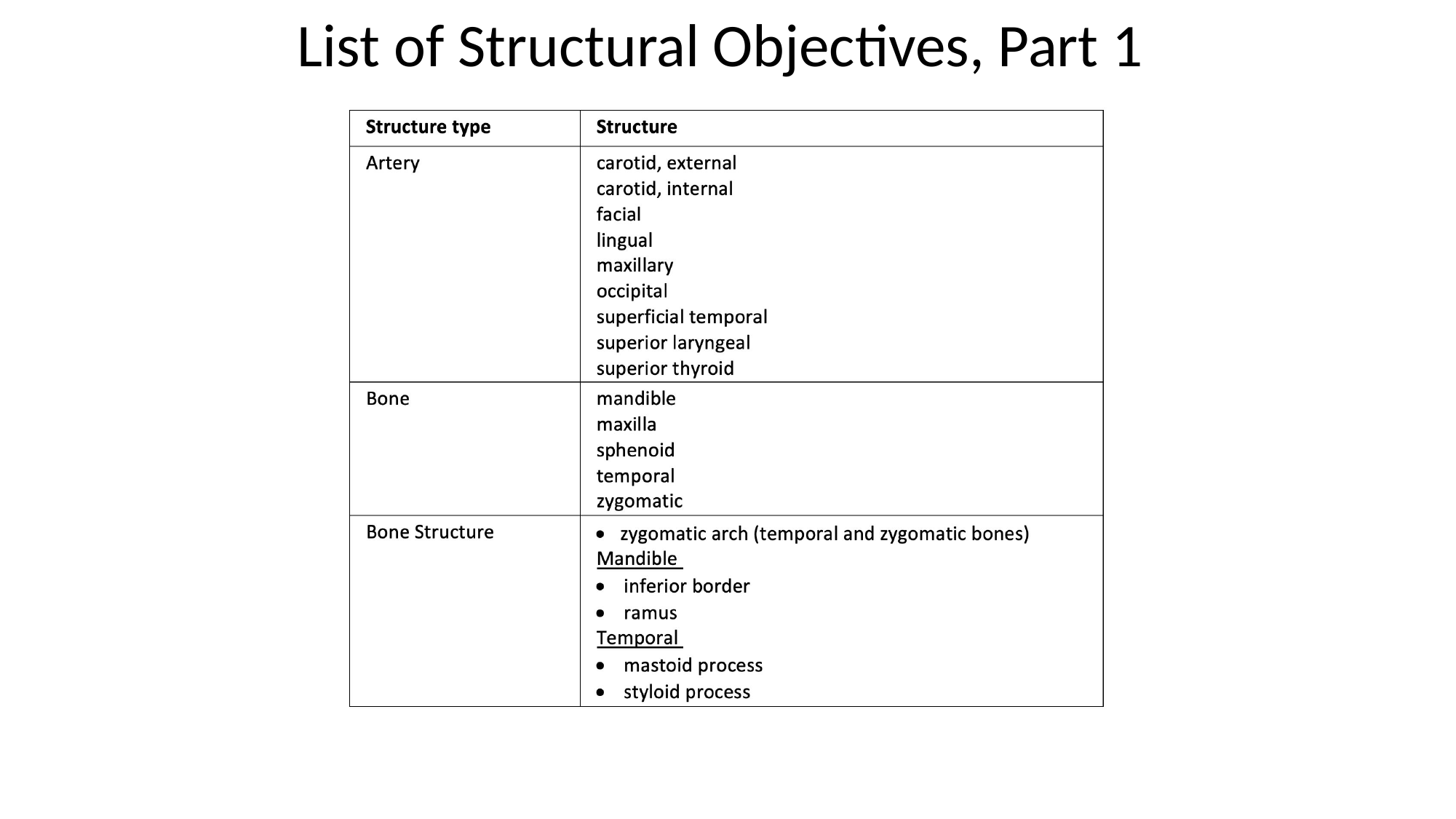

List of Structural Objectives, Part 1

## Slide 18
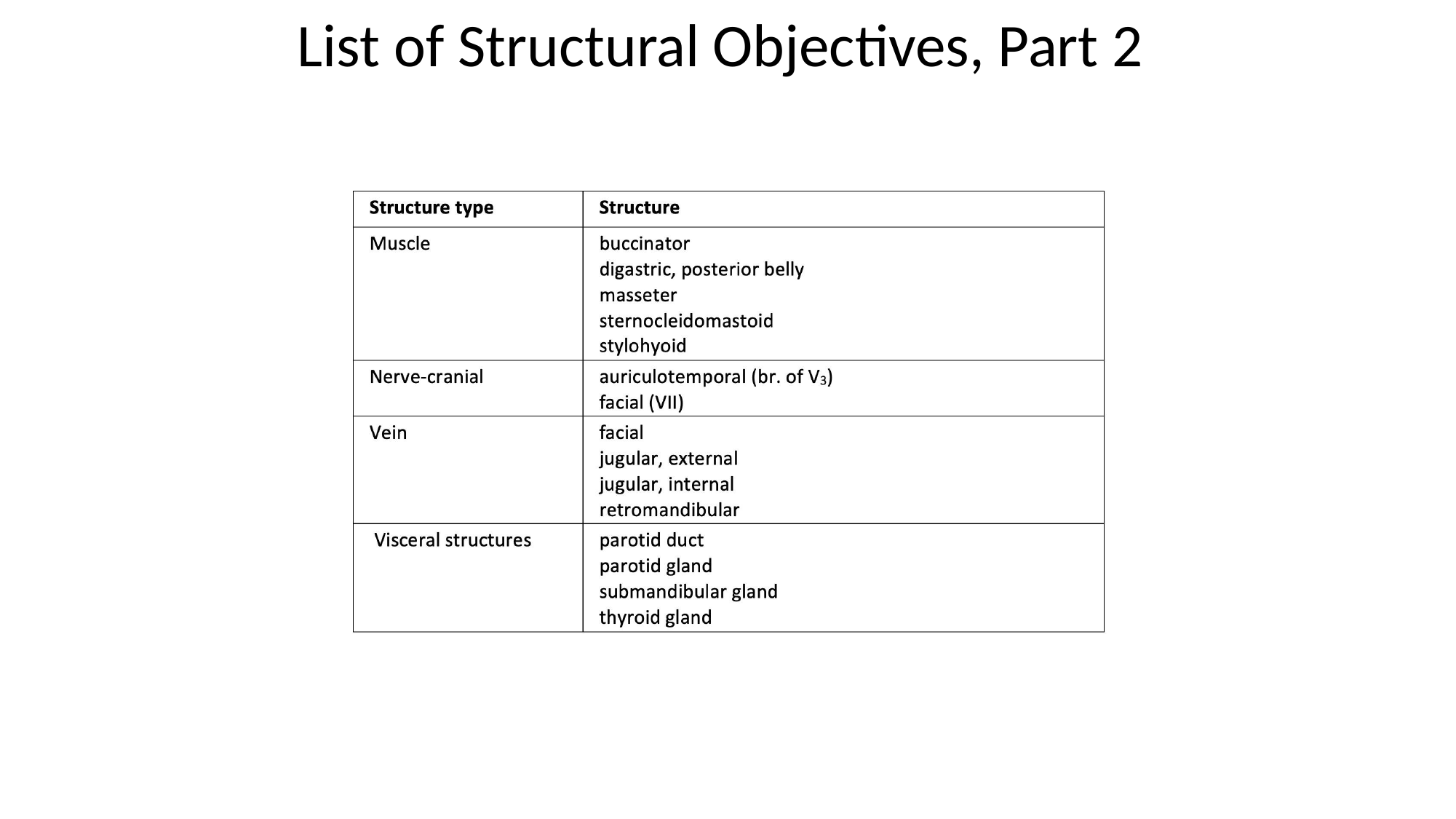

List of Structural Objectives, Part 2
