# Supplementary material for: Using the Situated Learning-Guided Educational Framework to Teach Anatomy of the Infratemporal Fossa and Retromandibular Region
Source: MedEdPORTAL. 2025 Oct 3;21:11550. doi: 10.15766/mep_2374-8265.11550 (PMC12491565; doi:10.15766/mep_2374-8265.11550)
Supplement: Supplementary file 1 — Infratemporal Fossa Module (Instructor).pptxRetromandibular Region Module (Instructor).pptxInfratemporal Fossa Module (Student).pptxRetromandibular Region Module (Student).pptxPretest.docxPosttest.docxSurvey - Infratemporal Fossa.docxSurvey - Retromandibular Region.docx [file mep_2374-8265.11550-s001.zip › A. Infratemporal Fossa Module (Instructor).pptx]

## Slide 1
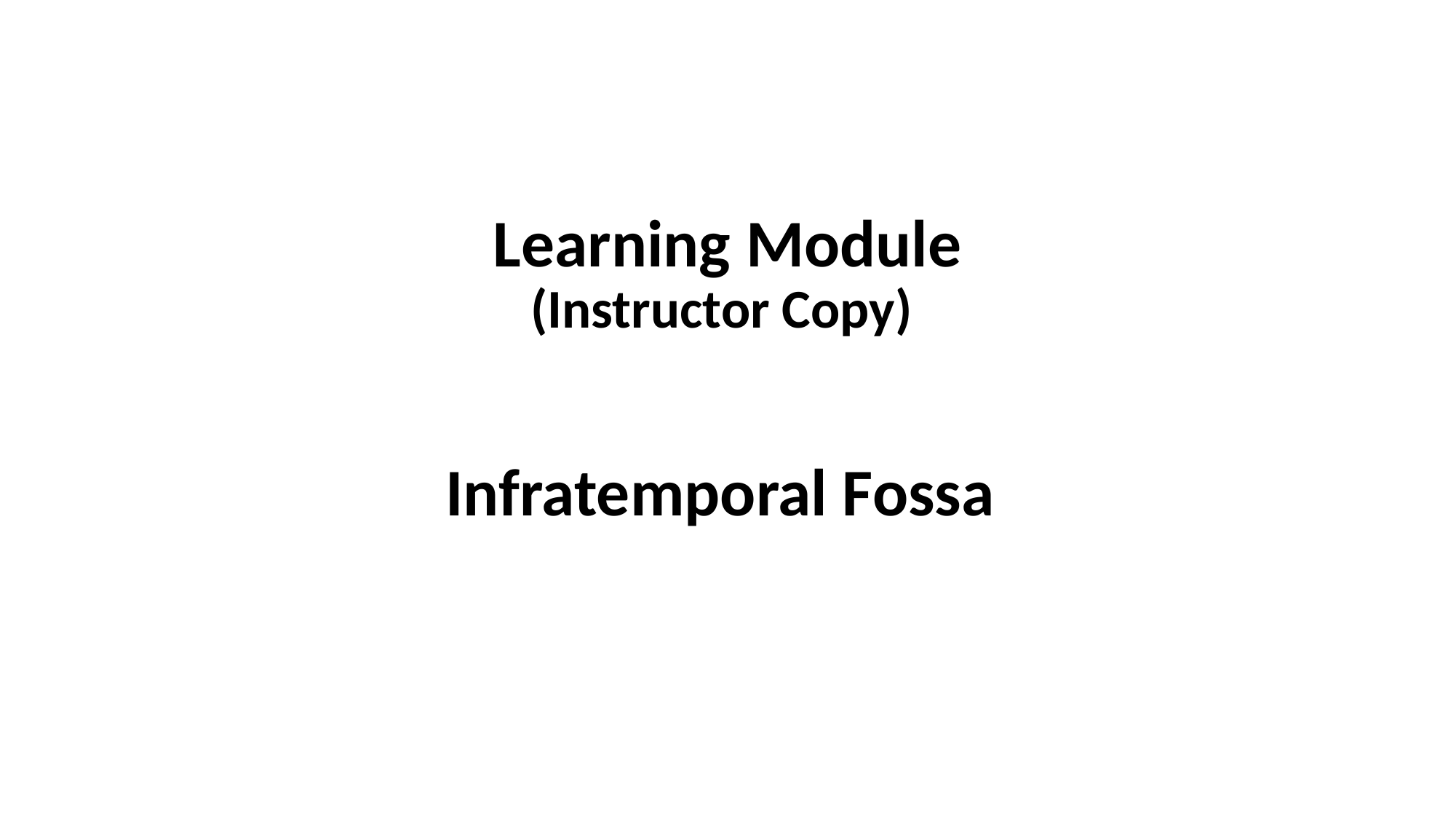

# Learning Module (Instructor Copy) Infratemporal Fossa

## Slide 2
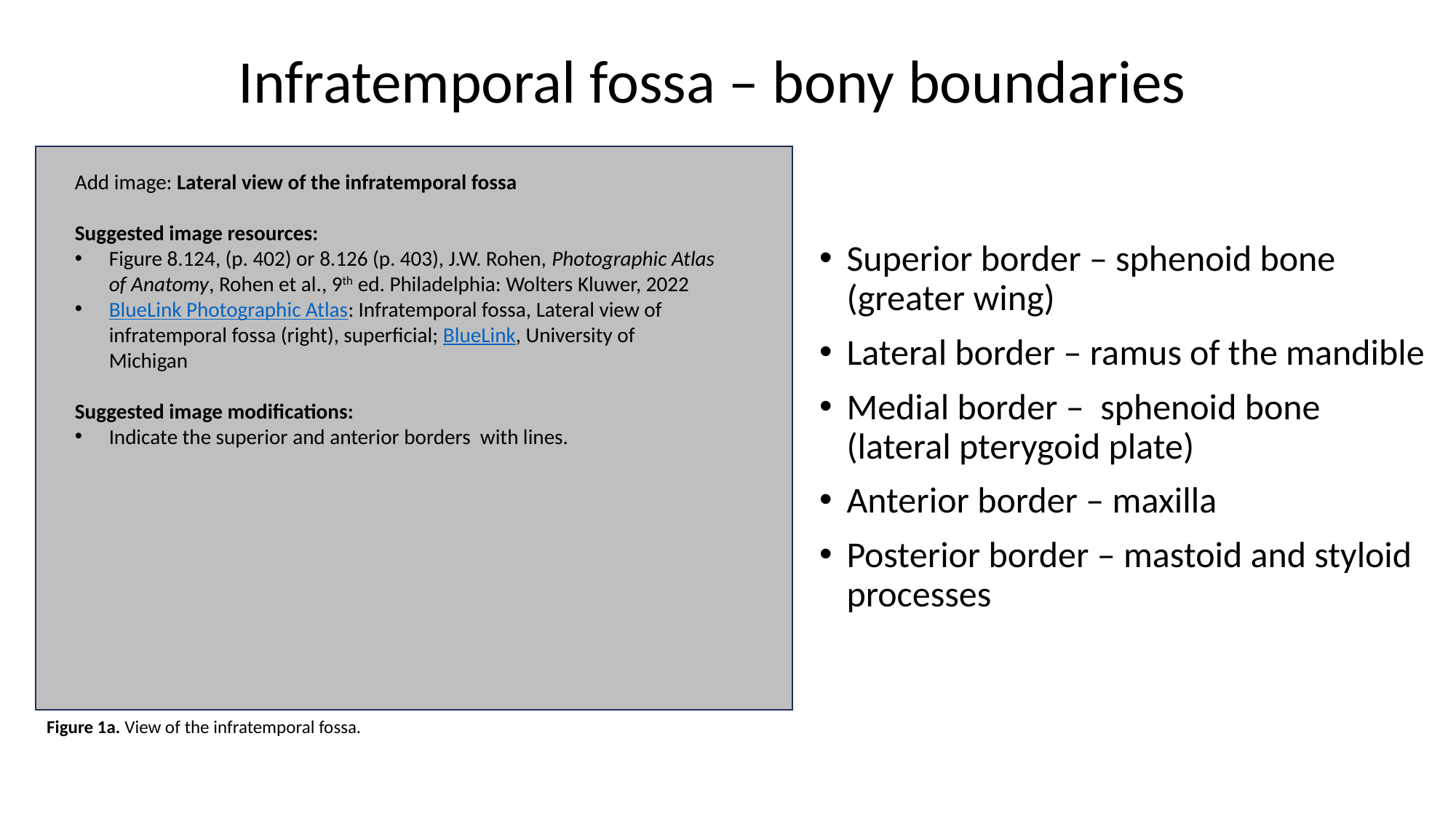

# Infratemporal fossa – bony boundaries
Add image: Lateral view of the infratemporal fossa
Suggested image resources:
Figure 8.124, (p. 402) or 8.126 (p. 403), J.W. Rohen, Photographic Atlas of Anatomy, Rohen et al., 9th ed. Philadelphia: Wolters Kluwer, 2022
BlueLink Photographic Atlas: Infratemporal fossa, Lateral view of infratemporal fossa (right), superficial; BlueLink, University of Michigan
Suggested image modifications:
Indicate the superior and anterior borders with lines.
Superior border – sphenoid bone (greater wing)
Lateral border – ramus of the mandible
Medial border – sphenoid bone (lateral pterygoid plate)
Anterior border – maxilla
Posterior border – mastoid and styloid processes
Figure 1a. View of the infratemporal fossa.

## Slide 3
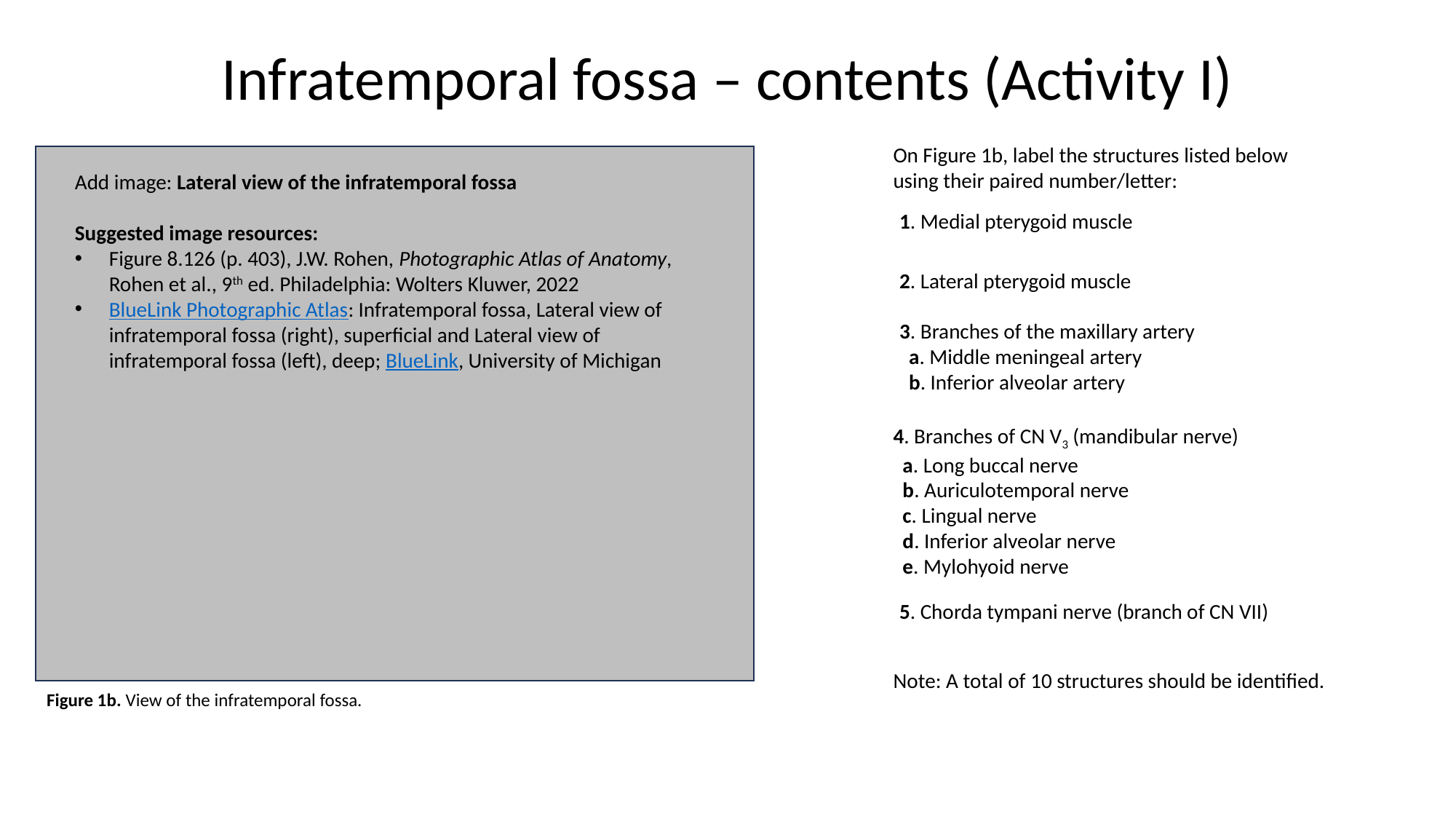

# Infratemporal fossa – contents (Activity I)
On Figure 1b, label the structures listed below using their paired number/letter:
Add image: Lateral view of the infratemporal fossa
Suggested image resources:
Figure 8.126 (p. 403), J.W. Rohen, Photographic Atlas of Anatomy, Rohen et al., 9th ed. Philadelphia: Wolters Kluwer, 2022
BlueLink Photographic Atlas: Infratemporal fossa, Lateral view of infratemporal fossa (right), superficial and Lateral view of infratemporal fossa (left), deep; BlueLink, University of Michigan
1. Medial pterygoid muscle
Right Ear
2. Lateral pterygoid muscle
3. Branches of the maxillary artery
 a. Middle meningeal artery
 b. Inferior alveolar artery
4. Branches of CN V3 (mandibular nerve)
 a. Long buccal nerve
 b. Auriculotemporal nerve
 c. Lingual nerve
 d. Inferior alveolar nerve
 e. Mylohyoid nerve
Parotid gland
5. Chorda tympani nerve (branch of CN VII)
Masseter
Note: A total of 10 structures should be identified.
Figure 1b. View of the infratemporal fossa.

## Slide 4
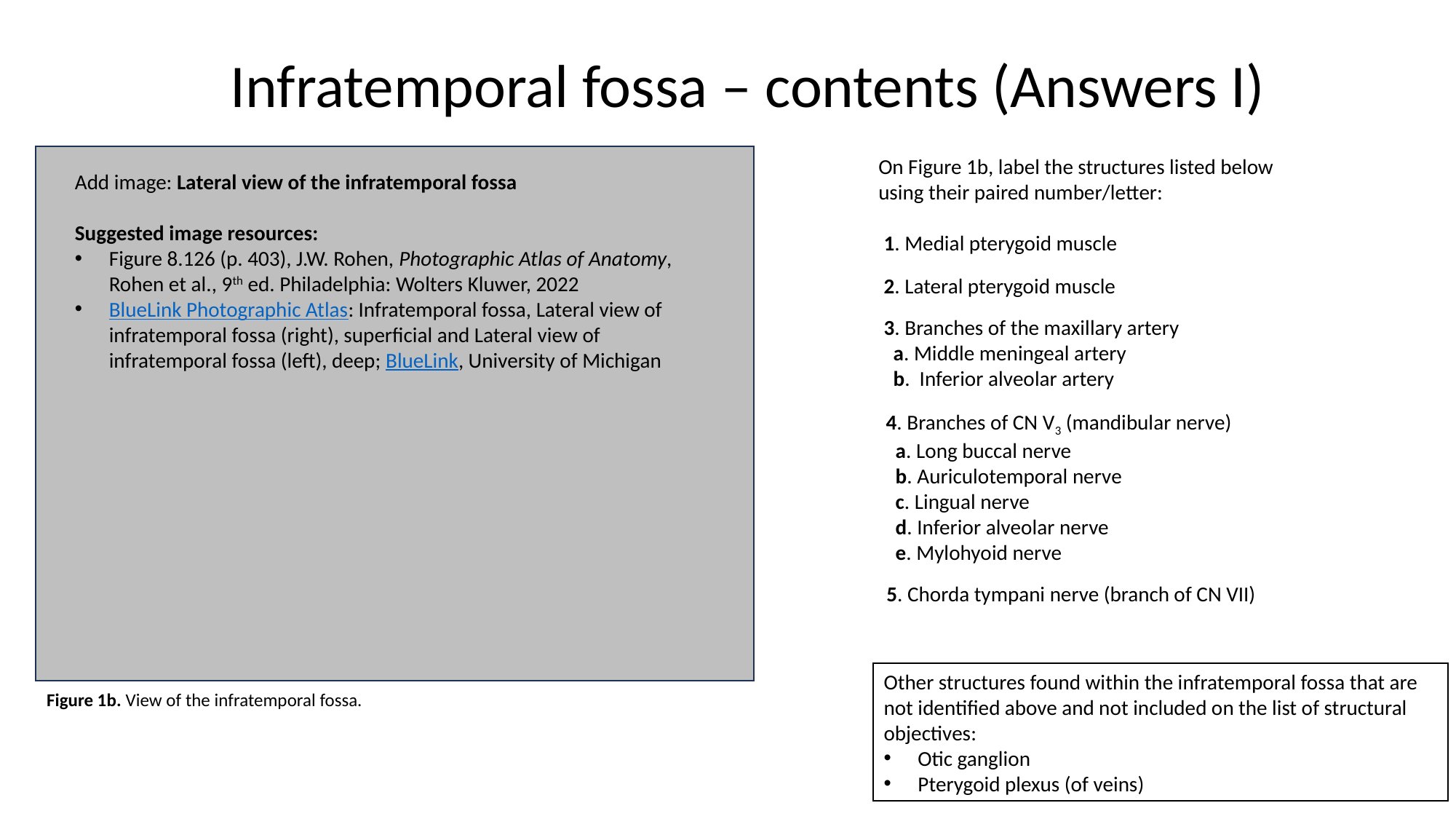

# Infratemporal fossa – contents (Answers I)
On Figure 1b, label the structures listed below using their paired number/letter:
Add image: Lateral view of the infratemporal fossa
Suggested image resources:
Figure 8.126 (p. 403), J.W. Rohen, Photographic Atlas of Anatomy, Rohen et al., 9th ed. Philadelphia: Wolters Kluwer, 2022
BlueLink Photographic Atlas: Infratemporal fossa, Lateral view of infratemporal fossa (right), superficial and Lateral view of infratemporal fossa (left), deep; BlueLink, University of Michigan
1. Medial pterygoid muscle
2. Lateral pterygoid muscle
3. Branches of the maxillary artery
 a. Middle meningeal artery
 b. Inferior alveolar artery
4. Branches of CN V3 (mandibular nerve)
 a. Long buccal nerve
 b. Auriculotemporal nerve
 c. Lingual nerve
 d. Inferior alveolar nerve
 e. Mylohyoid nerve
5. Chorda tympani nerve (branch of CN VII)
Other structures found within the infratemporal fossa that are not identified above and not included on the list of structural objectives:
Otic ganglion
Pterygoid plexus (of veins)
Figure 1b. View of the infratemporal fossa.

## Slide 5
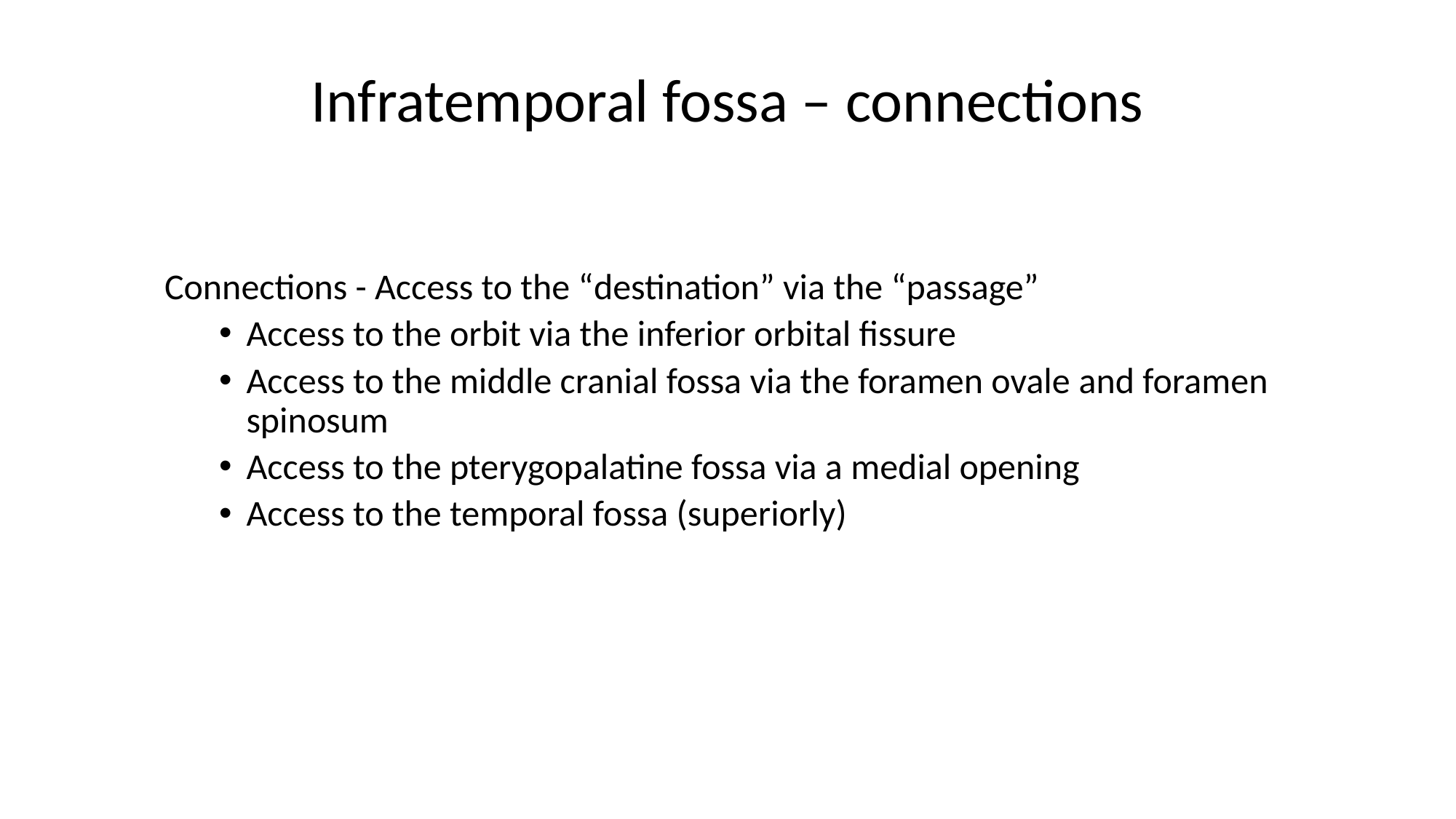

# Infratemporal fossa – connections
Connections - Access to the “destination” via the “passage”
Access to the orbit via the inferior orbital fissure
Access to the middle cranial fossa via the foramen ovale and foramen spinosum
Access to the pterygopalatine fossa via a medial opening
Access to the temporal fossa (superiorly)

## Slide 6
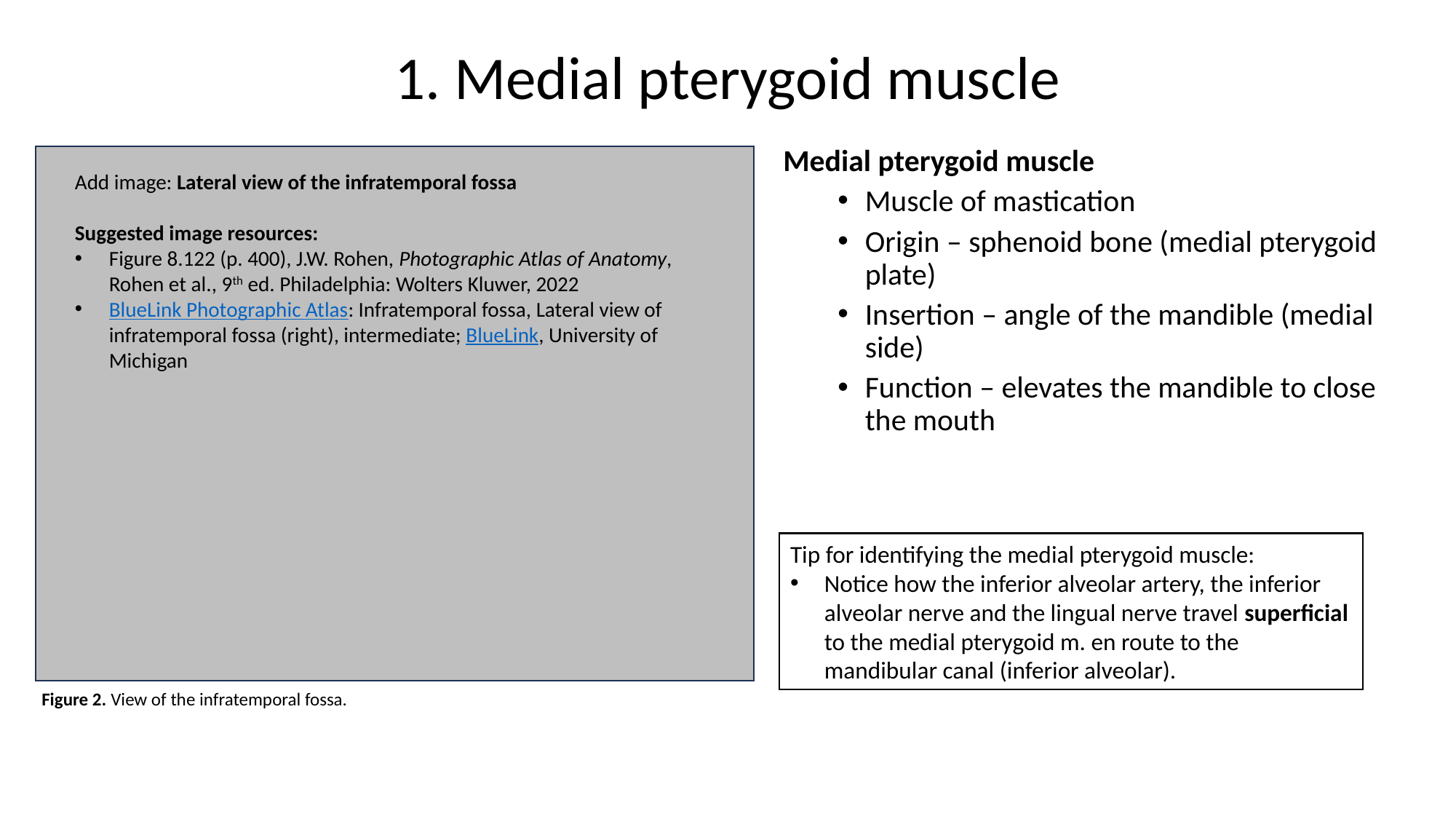

# 1. Medial pterygoid muscle
Medial pterygoid muscle
Muscle of mastication
Origin – sphenoid bone (medial pterygoid plate)
Insertion – angle of the mandible (medial side)
Function – elevates the mandible to close the mouth
Add image: Lateral view of the infratemporal fossa
Suggested image resources:
Figure 8.122 (p. 400), J.W. Rohen, Photographic Atlas of Anatomy, Rohen et al., 9th ed. Philadelphia: Wolters Kluwer, 2022
BlueLink Photographic Atlas: Infratemporal fossa, Lateral view of infratemporal fossa (right), intermediate; BlueLink, University of Michigan
Tip for identifying the medial pterygoid muscle:
Notice how the inferior alveolar artery, the inferior alveolar nerve and the lingual nerve travel superficial to the medial pterygoid m. en route to the mandibular canal (inferior alveolar).
Figure 2. View of the infratemporal fossa.

## Slide 7
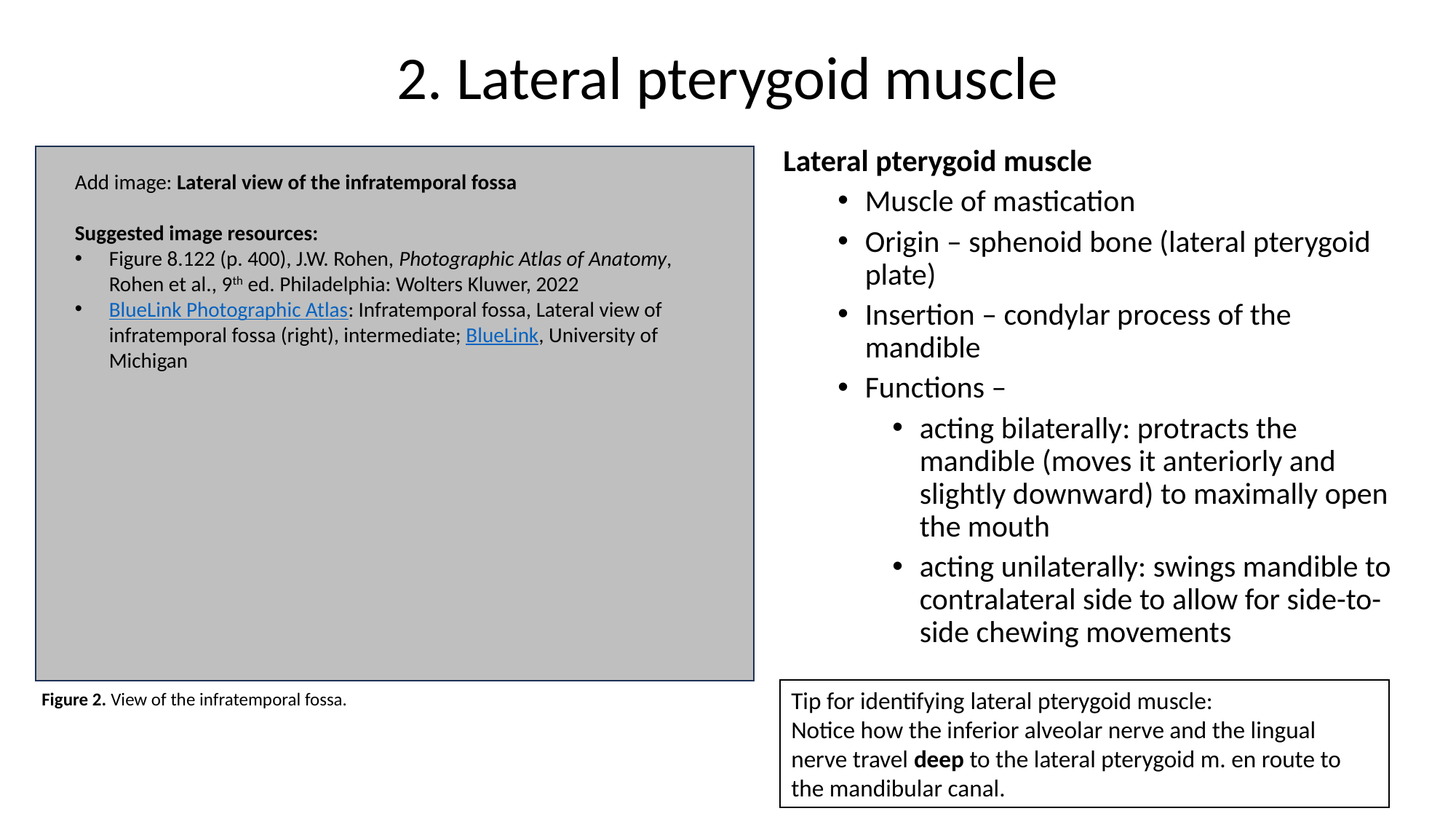

# 2. Lateral pterygoid muscle
Lateral pterygoid muscle
Muscle of mastication
Origin – sphenoid bone (lateral pterygoid plate)
Insertion – condylar process of the mandible
Functions –
acting bilaterally: protracts the mandible (moves it anteriorly and slightly downward) to maximally open the mouth
acting unilaterally: swings mandible to contralateral side to allow for side-to-side chewing movements
Add image: Lateral view of the infratemporal fossa
Suggested image resources:
Figure 8.122 (p. 400), J.W. Rohen, Photographic Atlas of Anatomy, Rohen et al., 9th ed. Philadelphia: Wolters Kluwer, 2022
BlueLink Photographic Atlas: Infratemporal fossa, Lateral view of infratemporal fossa (right), intermediate; BlueLink, University of Michigan
Tip for identifying lateral pterygoid muscle:
Notice how the inferior alveolar nerve and the lingual nerve travel deep to the lateral pterygoid m. en route to the mandibular canal.
Figure 2. View of the infratemporal fossa.

## Slide 8
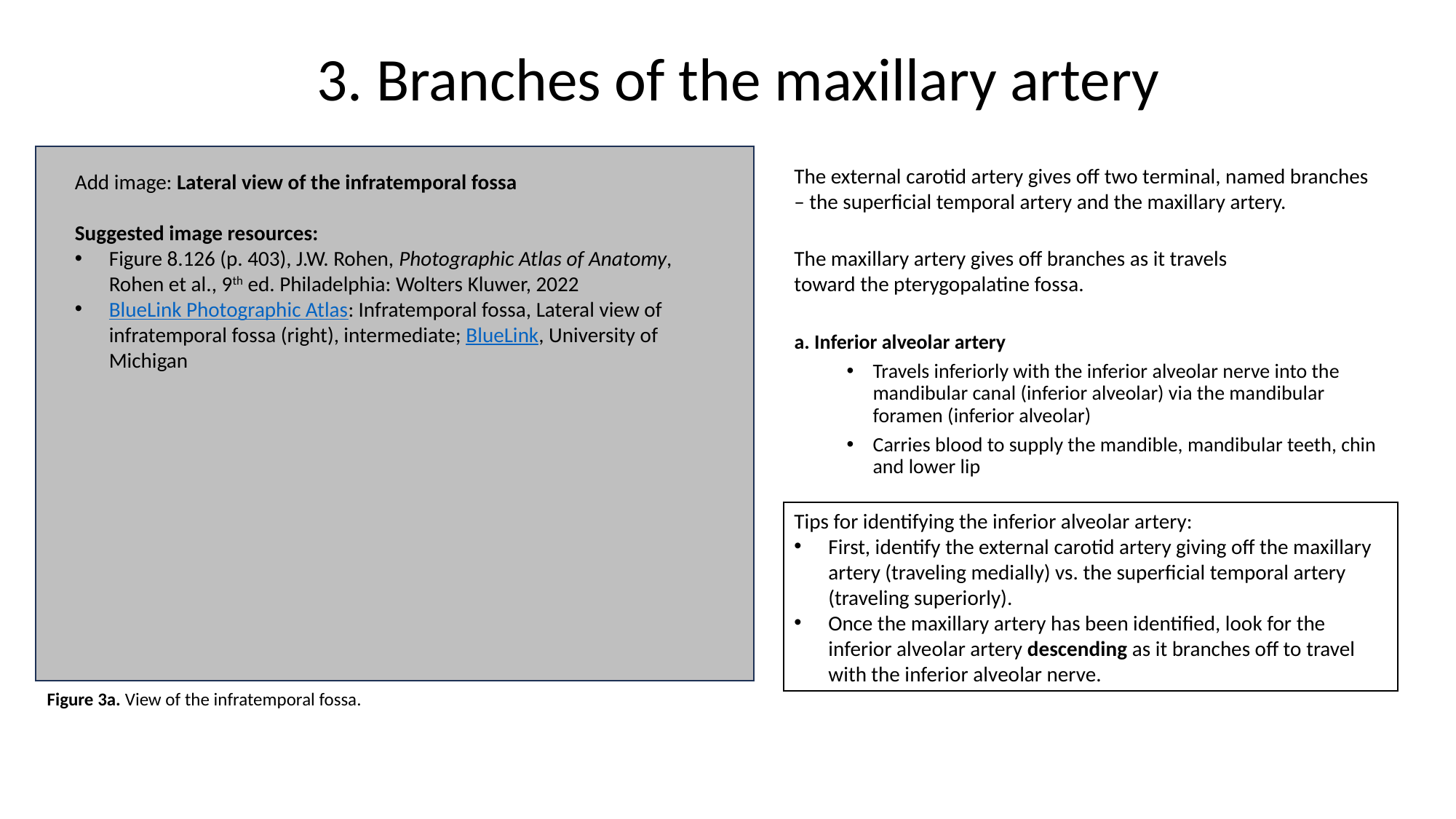

# 3. Branches of the maxillary artery
The external carotid artery gives off two terminal, named branches – the superficial temporal artery and the maxillary artery.
Add image: Lateral view of the infratemporal fossa
Suggested image resources:
Figure 8.126 (p. 403), J.W. Rohen, Photographic Atlas of Anatomy, Rohen et al., 9th ed. Philadelphia: Wolters Kluwer, 2022
BlueLink Photographic Atlas: Infratemporal fossa, Lateral view of infratemporal fossa (right), intermediate; BlueLink, University of Michigan
The maxillary artery gives off branches as it travels toward the pterygopalatine fossa.
a. Inferior alveolar artery
Travels inferiorly with the inferior alveolar nerve into the mandibular canal (inferior alveolar) via the mandibular foramen (inferior alveolar)
Carries blood to supply the mandible, mandibular teeth, chin and lower lip
Tips for identifying the inferior alveolar artery:
First, identify the external carotid artery giving off the maxillary artery (traveling medially) vs. the superficial temporal artery (traveling superiorly).
Once the maxillary artery has been identified, look for the inferior alveolar artery descending as it branches off to travel with the inferior alveolar nerve.
Figure 3a. View of the infratemporal fossa.

## Slide 9
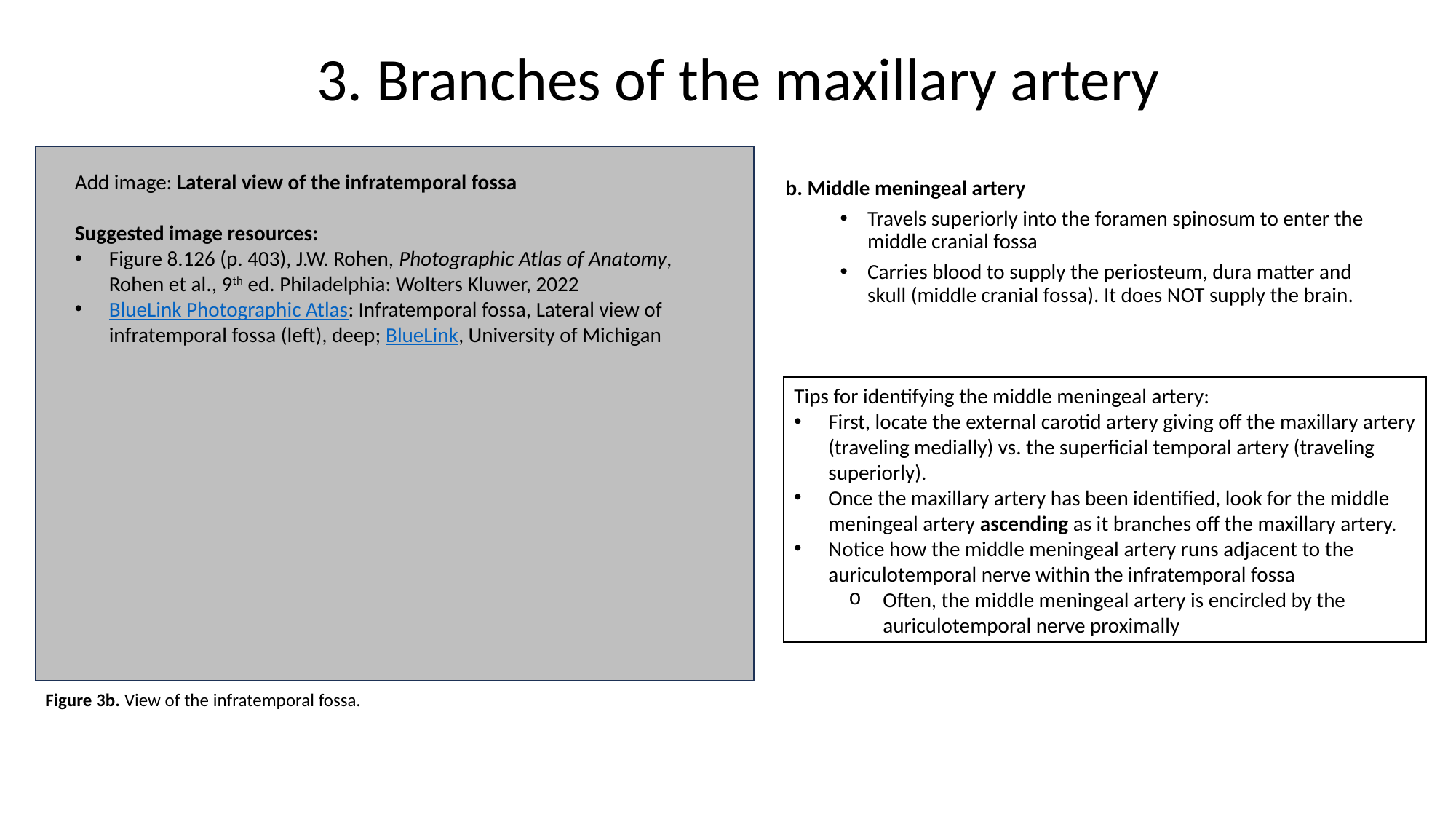

# 3. Branches of the maxillary artery
Add image: Lateral view of the infratemporal fossa
Suggested image resources:
Figure 8.126 (p. 403), J.W. Rohen, Photographic Atlas of Anatomy, Rohen et al., 9th ed. Philadelphia: Wolters Kluwer, 2022
BlueLink Photographic Atlas: Infratemporal fossa, Lateral view of infratemporal fossa (left), deep; BlueLink, University of Michigan
b. Middle meningeal artery
Travels superiorly into the foramen spinosum to enter the middle cranial fossa
Carries blood to supply the periosteum, dura matter and skull (middle cranial fossa). It does NOT supply the brain.
Tips for identifying the middle meningeal artery:
First, locate the external carotid artery giving off the maxillary artery (traveling medially) vs. the superficial temporal artery (traveling superiorly).
Once the maxillary artery has been identified, look for the middle meningeal artery ascending as it branches off the maxillary artery.
Notice how the middle meningeal artery runs adjacent to the auriculotemporal nerve within the infratemporal fossa
Often, the middle meningeal artery is encircled by the auriculotemporal nerve proximally
Figure 3b. View of the infratemporal fossa.

## Slide 10
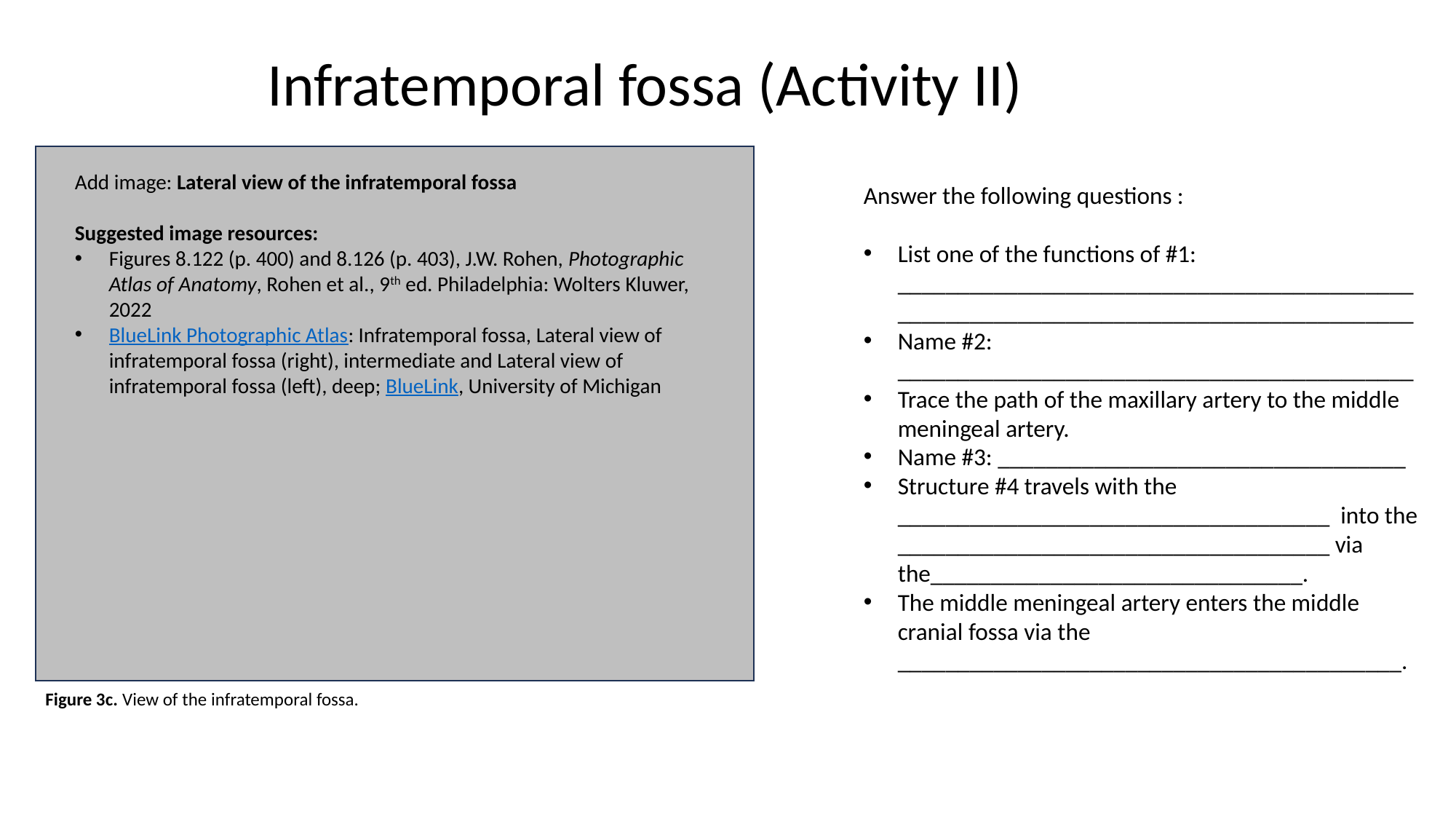

# Infratemporal fossa (Activity II)
Add image: Lateral view of the infratemporal fossa
Suggested image resources:
Figures 8.122 (p. 400) and 8.126 (p. 403), J.W. Rohen, Photographic Atlas of Anatomy, Rohen et al., 9th ed. Philadelphia: Wolters Kluwer, 2022
BlueLink Photographic Atlas: Infratemporal fossa, Lateral view of infratemporal fossa (right), intermediate and Lateral view of infratemporal fossa (left), deep; BlueLink, University of Michigan
Answer the following questions :
List one of the functions of #1: ______________________________________________________________________________________
Name #2: ___________________________________________
Trace the path of the maxillary artery to the middle meningeal artery.
Name #3: __________________________________
Structure #4 travels with the ____________________________________ into the ____________________________________ via the_______________________________.
The middle meningeal artery enters the middle cranial fossa via the __________________________________________.
Figure 3c. View of the infratemporal fossa.

## Slide 11
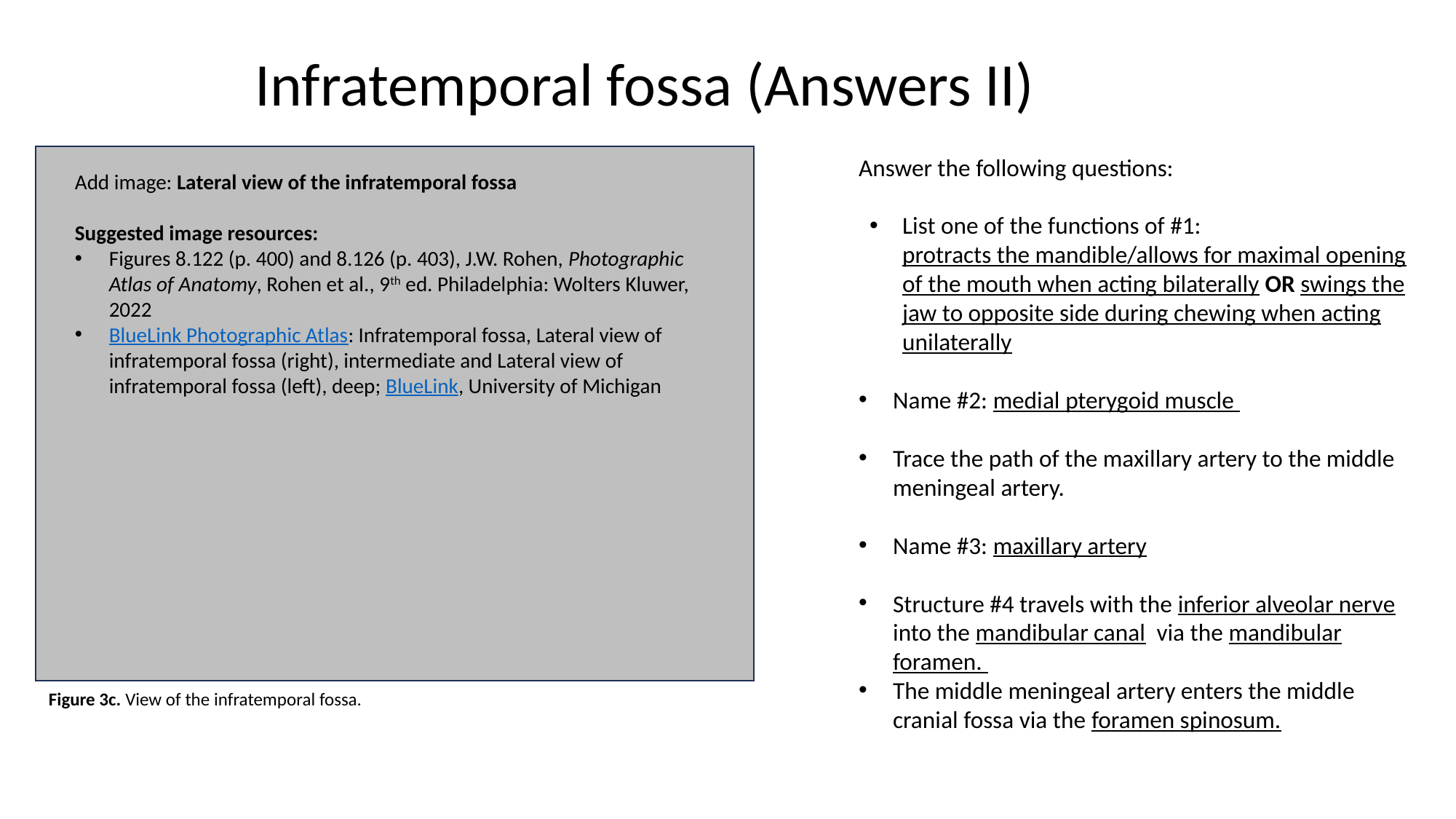

# Infratemporal fossa (Answers II)
Answer the following questions:
List one of the functions of #1:
protracts the mandible/allows for maximal opening of the mouth when acting bilaterally OR swings the jaw to opposite side during chewing when acting unilaterally
Name #2: medial pterygoid muscle
Trace the path of the maxillary artery to the middle meningeal artery.
Name #3: maxillary artery
Structure #4 travels with the inferior alveolar nerve into the mandibular canal via the mandibular foramen.
The middle meningeal artery enters the middle cranial fossa via the foramen spinosum.
Add image: Lateral view of the infratemporal fossa
Suggested image resources:
Figures 8.122 (p. 400) and 8.126 (p. 403), J.W. Rohen, Photographic Atlas of Anatomy, Rohen et al., 9th ed. Philadelphia: Wolters Kluwer, 2022
BlueLink Photographic Atlas: Infratemporal fossa, Lateral view of infratemporal fossa (right), intermediate and Lateral view of infratemporal fossa (left), deep; BlueLink, University of Michigan
Figure 3c. View of the infratemporal fossa.

## Slide 12
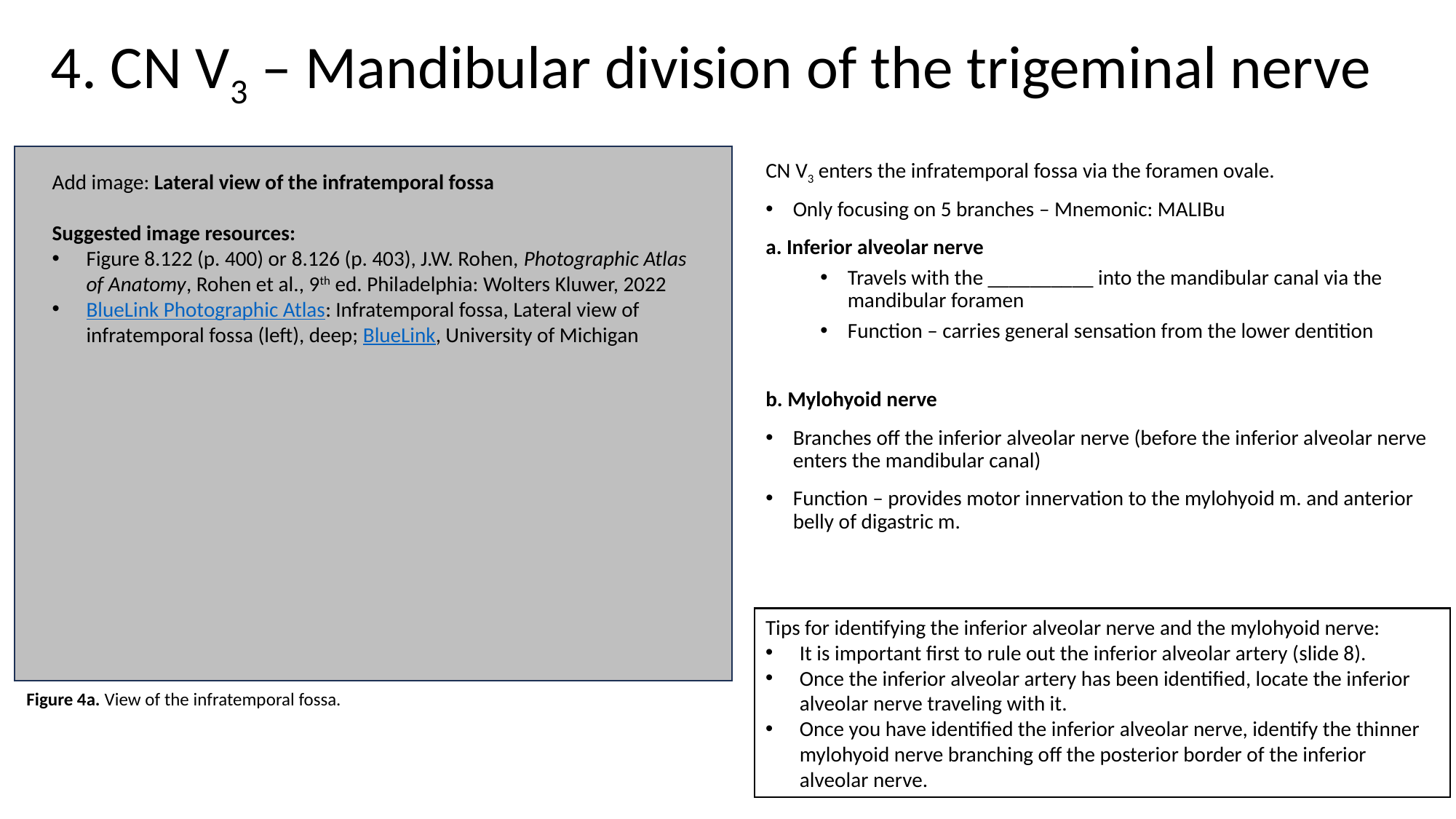

# 4. CN V3 – Mandibular division of the trigeminal nerve
CN V3 enters the infratemporal fossa via the foramen ovale.
Only focusing on 5 branches – Mnemonic: MALIBu
a. Inferior alveolar nerve
Travels with the __________ into the mandibular canal via the mandibular foramen
Function – carries general sensation from the lower dentition
b. Mylohyoid nerve
Branches off the inferior alveolar nerve (before the inferior alveolar nerve enters the mandibular canal)
Function – provides motor innervation to the mylohyoid m. and anterior belly of digastric m.
Add image: Lateral view of the infratemporal fossa
Suggested image resources:
Figure 8.122 (p. 400) or 8.126 (p. 403), J.W. Rohen, Photographic Atlas of Anatomy, Rohen et al., 9th ed. Philadelphia: Wolters Kluwer, 2022
BlueLink Photographic Atlas: Infratemporal fossa, Lateral view of infratemporal fossa (left), deep; BlueLink, University of Michigan
Tips for identifying the inferior alveolar nerve and the mylohyoid nerve:
It is important first to rule out the inferior alveolar artery (slide 8).
Once the inferior alveolar artery has been identified, locate the inferior alveolar nerve traveling with it.
Once you have identified the inferior alveolar nerve, identify the thinner mylohyoid nerve branching off the posterior border of the inferior alveolar nerve.
Figure 4a. View of the infratemporal fossa.

## Slide 13
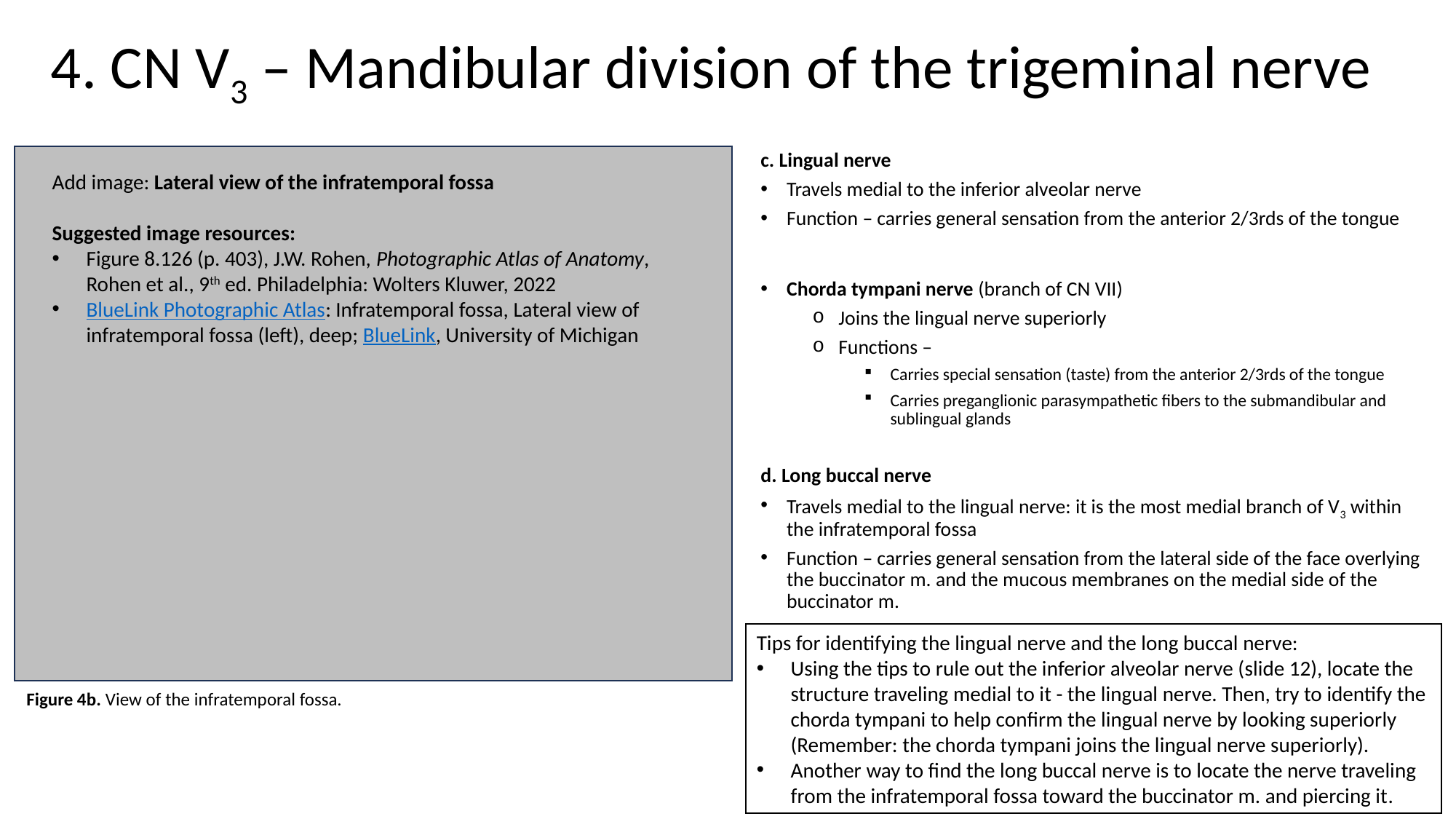

# 4. CN V3 – Mandibular division of the trigeminal nerve
c. Lingual nerve
Travels medial to the inferior alveolar nerve
Function – carries general sensation from the anterior 2/3rds of the tongue
Chorda tympani nerve (branch of CN VII)
Joins the lingual nerve superiorly
Functions –
Carries special sensation (taste) from the anterior 2/3rds of the tongue
Carries preganglionic parasympathetic fibers to the submandibular and sublingual glands
d. Long buccal nerve
Travels medial to the lingual nerve: it is the most medial branch of V3 within the infratemporal fossa
Function – carries general sensation from the lateral side of the face overlying the buccinator m. and the mucous membranes on the medial side of the buccinator m.
Add image: Lateral view of the infratemporal fossa
Suggested image resources:
Figure 8.126 (p. 403), J.W. Rohen, Photographic Atlas of Anatomy, Rohen et al., 9th ed. Philadelphia: Wolters Kluwer, 2022
BlueLink Photographic Atlas: Infratemporal fossa, Lateral view of infratemporal fossa (left), deep; BlueLink, University of Michigan
Tips for identifying the lingual nerve and the long buccal nerve:
Using the tips to rule out the inferior alveolar nerve (slide 12), locate the structure traveling medial to it - the lingual nerve. Then, try to identify the chorda tympani to help confirm the lingual nerve by looking superiorly (Remember: the chorda tympani joins the lingual nerve superiorly).
Another way to find the long buccal nerve is to locate the nerve traveling from the infratemporal fossa toward the buccinator m. and piercing it.
Figure 4b. View of the infratemporal fossa.

## Slide 14
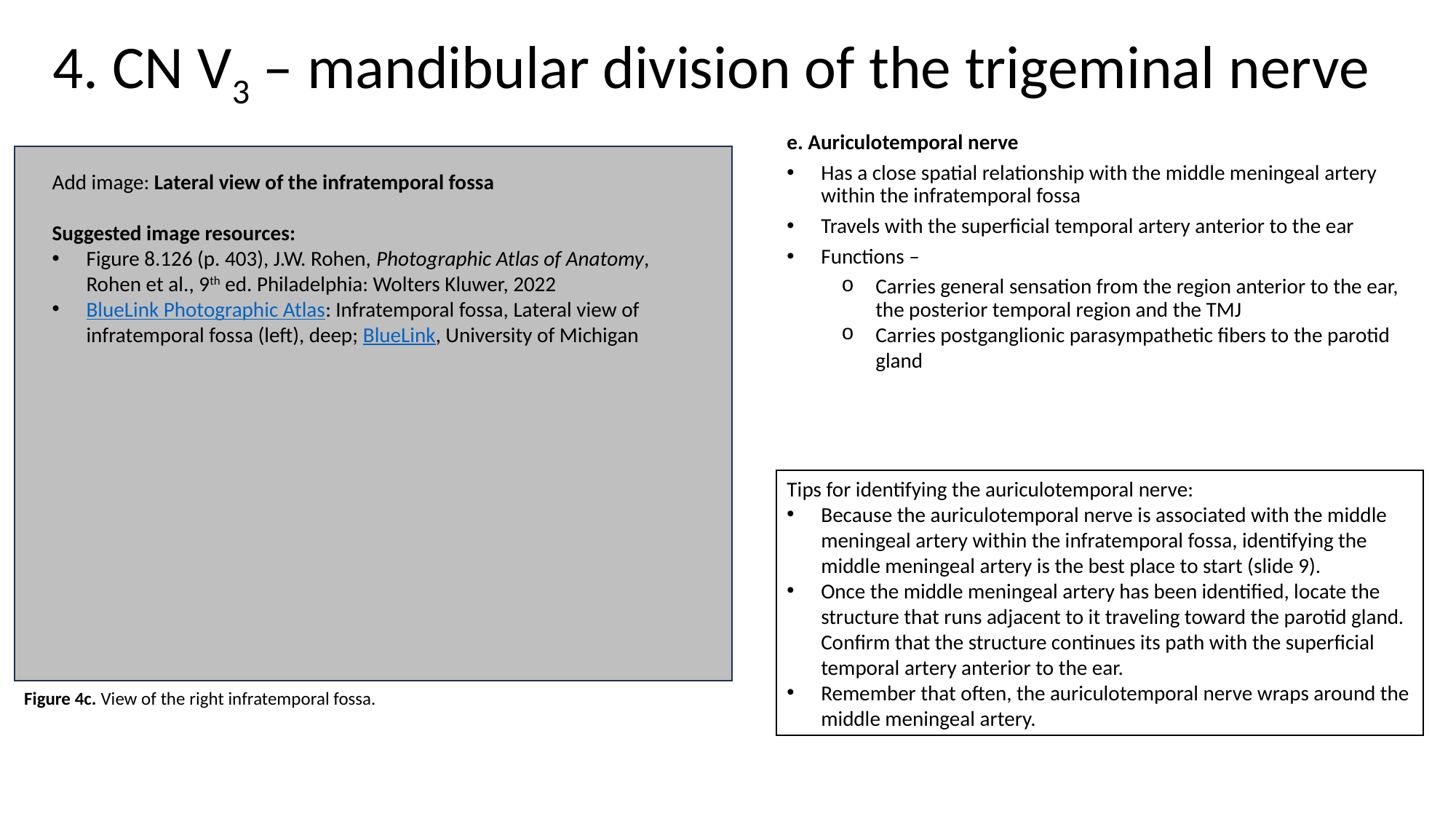

# 4. CN V3 – mandibular division of the trigeminal nerve
e. Auriculotemporal nerve
Has a close spatial relationship with the middle meningeal artery within the infratemporal fossa
Travels with the superficial temporal artery anterior to the ear
Functions –
Carries general sensation from the region anterior to the ear, the posterior temporal region and the TMJ
Carries postganglionic parasympathetic fibers to the parotid gland
Add image: Lateral view of the infratemporal fossa
Suggested image resources:
Figure 8.126 (p. 403), J.W. Rohen, Photographic Atlas of Anatomy, Rohen et al., 9th ed. Philadelphia: Wolters Kluwer, 2022
BlueLink Photographic Atlas: Infratemporal fossa, Lateral view of infratemporal fossa (left), deep; BlueLink, University of Michigan
Tips for identifying the auriculotemporal nerve:
Because the auriculotemporal nerve is associated with the middle meningeal artery within the infratemporal fossa, identifying the middle meningeal artery is the best place to start (slide 9).
Once the middle meningeal artery has been identified, locate the structure that runs adjacent to it traveling toward the parotid gland. Confirm that the structure continues its path with the superficial temporal artery anterior to the ear.
Remember that often, the auriculotemporal nerve wraps around the middle meningeal artery.
4
Figure 4c. View of the right infratemporal fossa.

## Slide 15
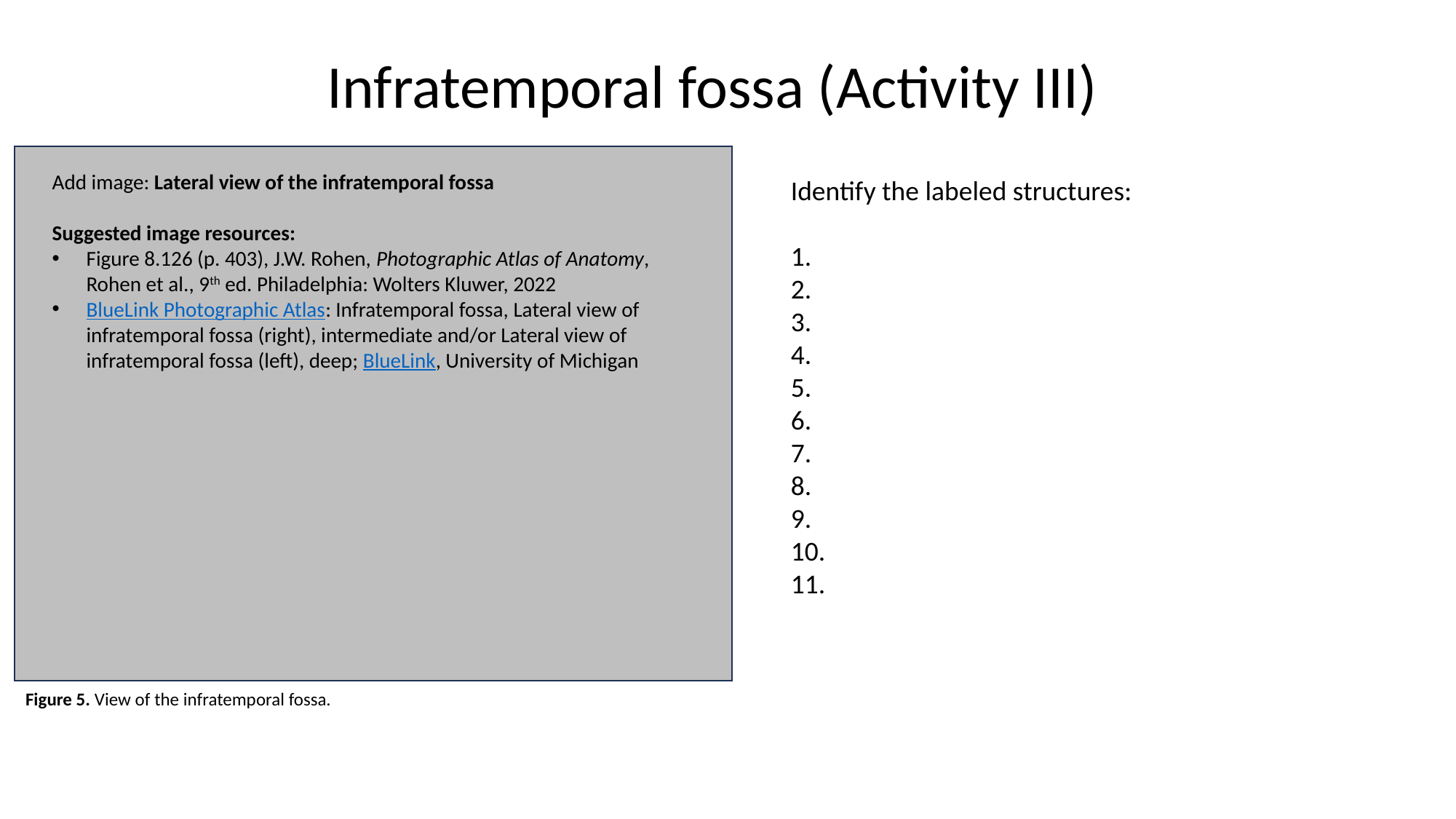

# Infratemporal fossa (Activity III)
Add image: Lateral view of the infratemporal fossa
Suggested image resources:
Figure 8.126 (p. 403), J.W. Rohen, Photographic Atlas of Anatomy, Rohen et al., 9th ed. Philadelphia: Wolters Kluwer, 2022
BlueLink Photographic Atlas: Infratemporal fossa, Lateral view of infratemporal fossa (right), intermediate and/or Lateral view of infratemporal fossa (left), deep; BlueLink, University of Michigan
Identify the labeled structures:
1.
2.
3.
4.
5.
6.
7.
8.
9.
10.
11.
Figure 5. View of the infratemporal fossa.

## Slide 16
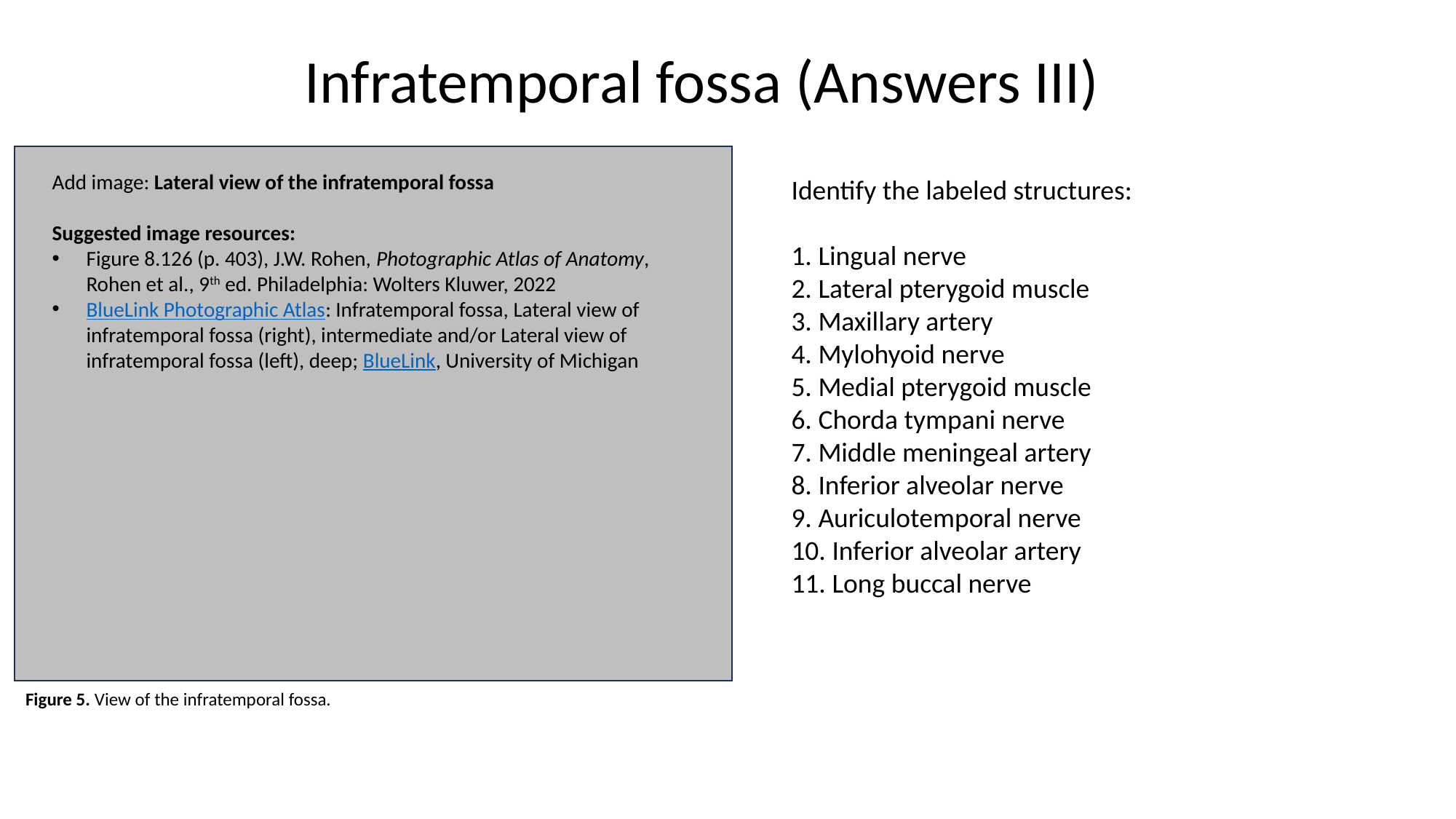

# Infratemporal fossa (Answers III)
Add image: Lateral view of the infratemporal fossa
Suggested image resources:
Figure 8.126 (p. 403), J.W. Rohen, Photographic Atlas of Anatomy, Rohen et al., 9th ed. Philadelphia: Wolters Kluwer, 2022
BlueLink Photographic Atlas: Infratemporal fossa, Lateral view of infratemporal fossa (right), intermediate and/or Lateral view of infratemporal fossa (left), deep; BlueLink, University of Michigan
Identify the labeled structures:
1. Lingual nerve
2. Lateral pterygoid muscle
3. Maxillary artery
4. Mylohyoid nerve
5. Medial pterygoid muscle
6. Chorda tympani nerve
7. Middle meningeal artery
8. Inferior alveolar nerve
9. Auriculotemporal nerve
10. Inferior alveolar artery
11. Long buccal nerve
Figure 5. View of the infratemporal fossa.

## Slide 17
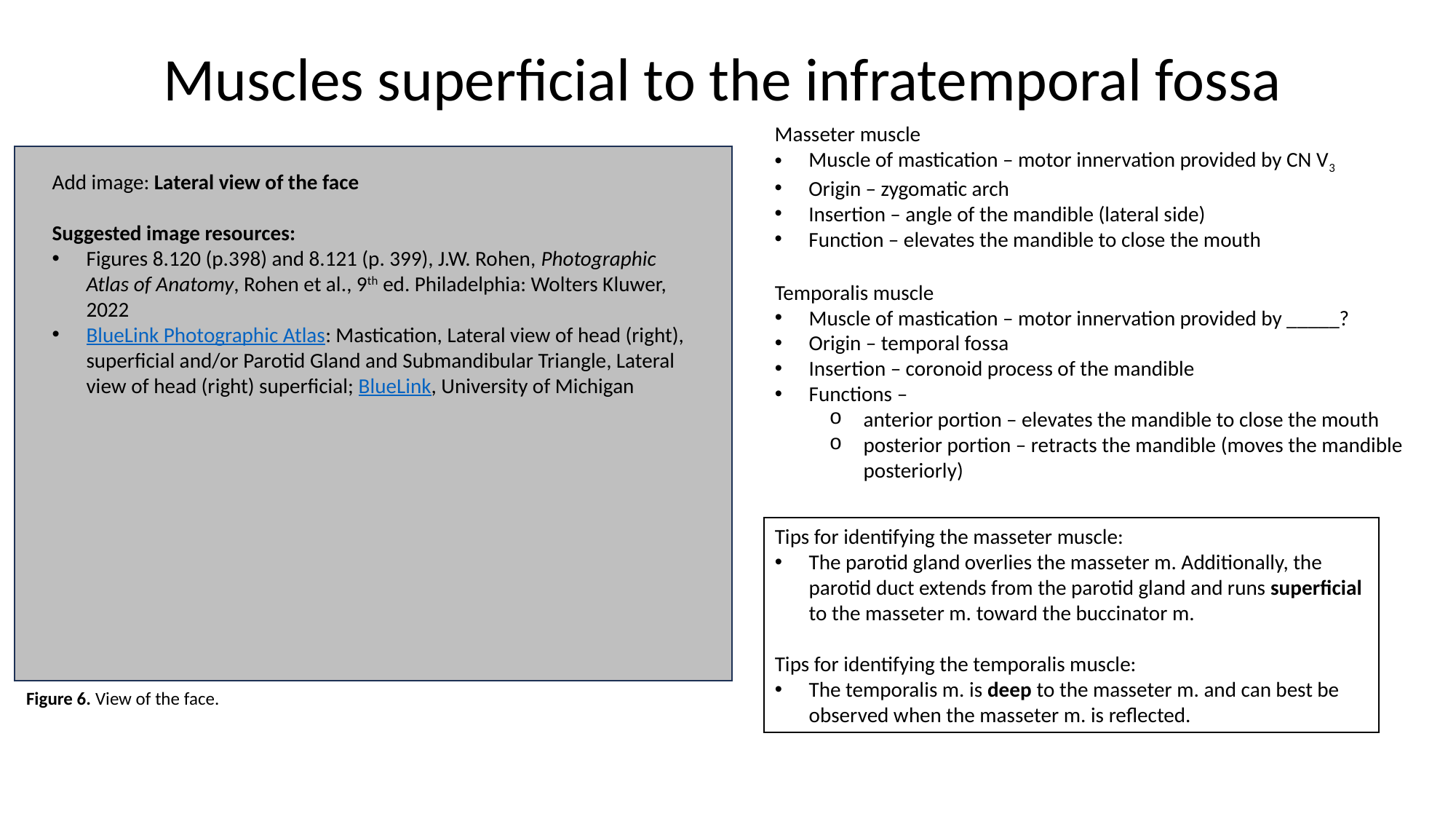

# Muscles superficial to the infratemporal fossa
Masseter muscle
Muscle of mastication – motor innervation provided by CN V3
Origin – zygomatic arch
Insertion – angle of the mandible (lateral side)
Function – elevates the mandible to close the mouth
Add image: Lateral view of the face
Suggested image resources:
Figures 8.120 (p.398) and 8.121 (p. 399), J.W. Rohen, Photographic Atlas of Anatomy, Rohen et al., 9th ed. Philadelphia: Wolters Kluwer, 2022
BlueLink Photographic Atlas: Mastication, Lateral view of head (right), superficial and/or Parotid Gland and Submandibular Triangle, Lateral view of head (right) superficial; BlueLink, University of Michigan
Temporalis muscle
Muscle of mastication – motor innervation provided by _____?
Origin – temporal fossa
Insertion – coronoid process of the mandible
Functions –
anterior portion – elevates the mandible to close the mouth
posterior portion – retracts the mandible (moves the mandible posteriorly)
Tips for identifying the masseter muscle:
The parotid gland overlies the masseter m. Additionally, the parotid duct extends from the parotid gland and runs superficial to the masseter m. toward the buccinator m.
Tips for identifying the temporalis muscle:
The temporalis m. is deep to the masseter m. and can best be observed when the masseter m. is reflected.
Figure 6. View of the face.

## Slide 18
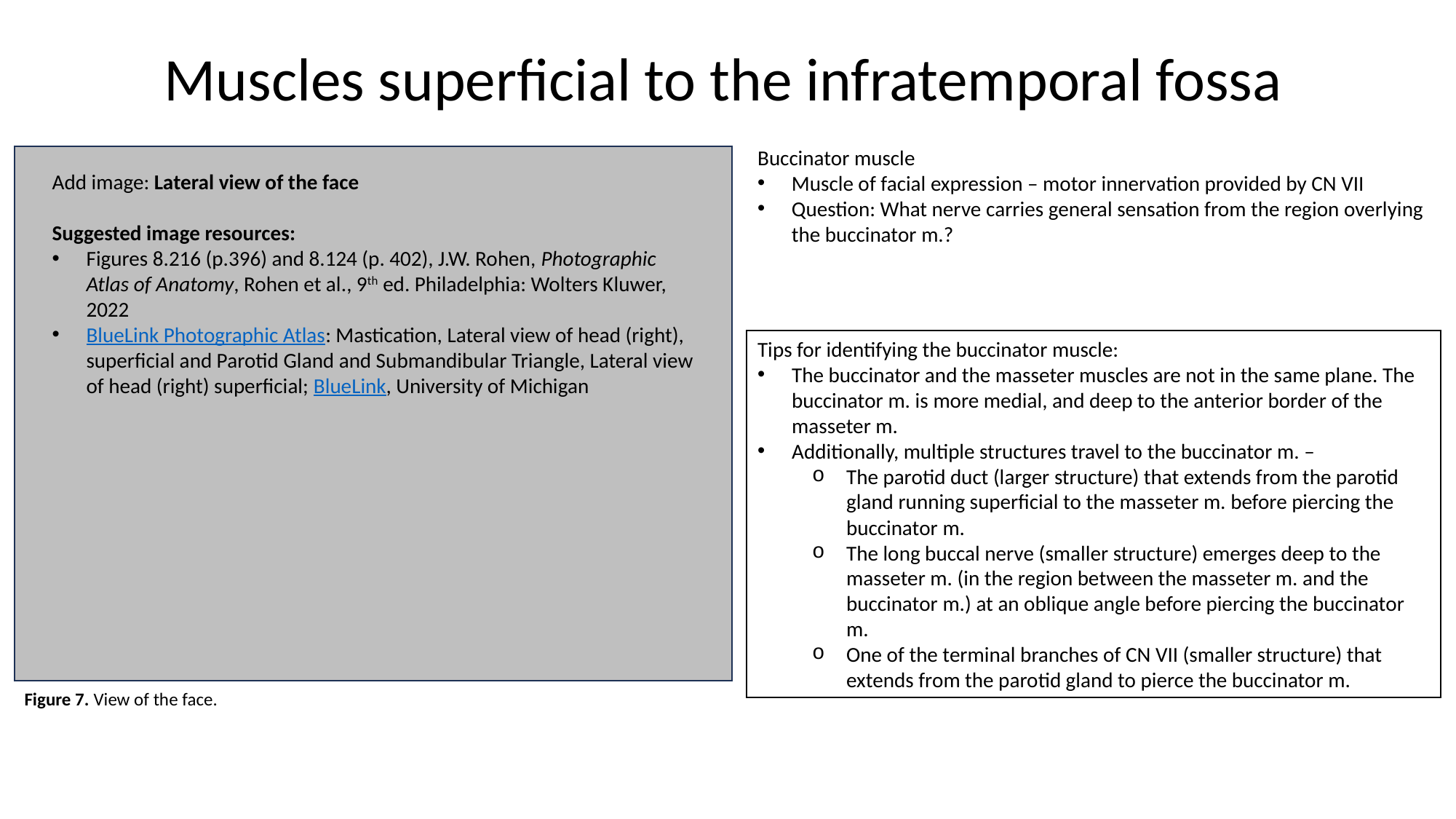

# Muscles superficial to the infratemporal fossa
Buccinator muscle
Muscle of facial expression – motor innervation provided by CN VII
Question: What nerve carries general sensation from the region overlying the buccinator m.?
Add image: Lateral view of the face
Suggested image resources:
Figures 8.216 (p.396) and 8.124 (p. 402), J.W. Rohen, Photographic Atlas of Anatomy, Rohen et al., 9th ed. Philadelphia: Wolters Kluwer, 2022
BlueLink Photographic Atlas: Mastication, Lateral view of head (right), superficial and Parotid Gland and Submandibular Triangle, Lateral view of head (right) superficial; BlueLink, University of Michigan
Tips for identifying the buccinator muscle:
The buccinator and the masseter muscles are not in the same plane. The buccinator m. is more medial, and deep to the anterior border of the masseter m.
Additionally, multiple structures travel to the buccinator m. –
The parotid duct (larger structure) that extends from the parotid gland running superficial to the masseter m. before piercing the buccinator m.
The long buccal nerve (smaller structure) emerges deep to the masseter m. (in the region between the masseter m. and the buccinator m.) at an oblique angle before piercing the buccinator m.
One of the terminal branches of CN VII (smaller structure) that extends from the parotid gland to pierce the buccinator m.
Figure 7. View of the face.

## Slide 19
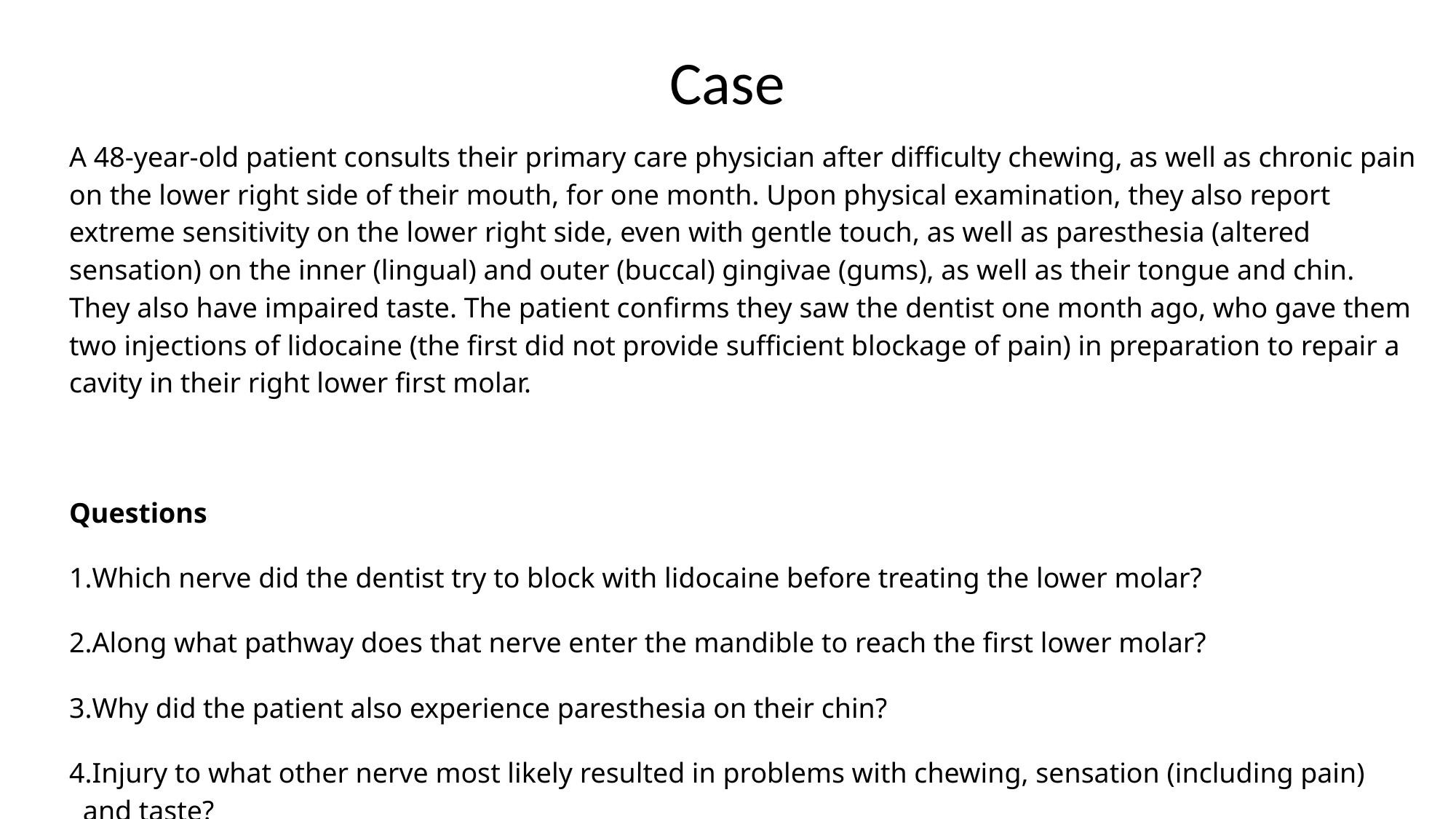

# Case
A 48-year-old patient consults their primary care physician after difficulty chewing, as well as chronic pain on the lower right side of their mouth, for one month. Upon physical examination, they also report extreme sensitivity on the lower right side, even with gentle touch, as well as paresthesia (altered sensation) on the inner (lingual) and outer (buccal) gingivae (gums), as well as their tongue and chin. They also have impaired taste. The patient confirms they saw the dentist one month ago, who gave them two injections of lidocaine (the first did not provide sufficient blockage of pain) in preparation to repair a cavity in their right lower first molar.
Questions
Which nerve did the dentist try to block with lidocaine before treating the lower molar?
Along what pathway does that nerve enter the mandible to reach the first lower molar?
Why did the patient also experience paresthesia on their chin?
Injury to what other nerve most likely resulted in problems with chewing, sensation (including pain) and taste?

## Slide 20
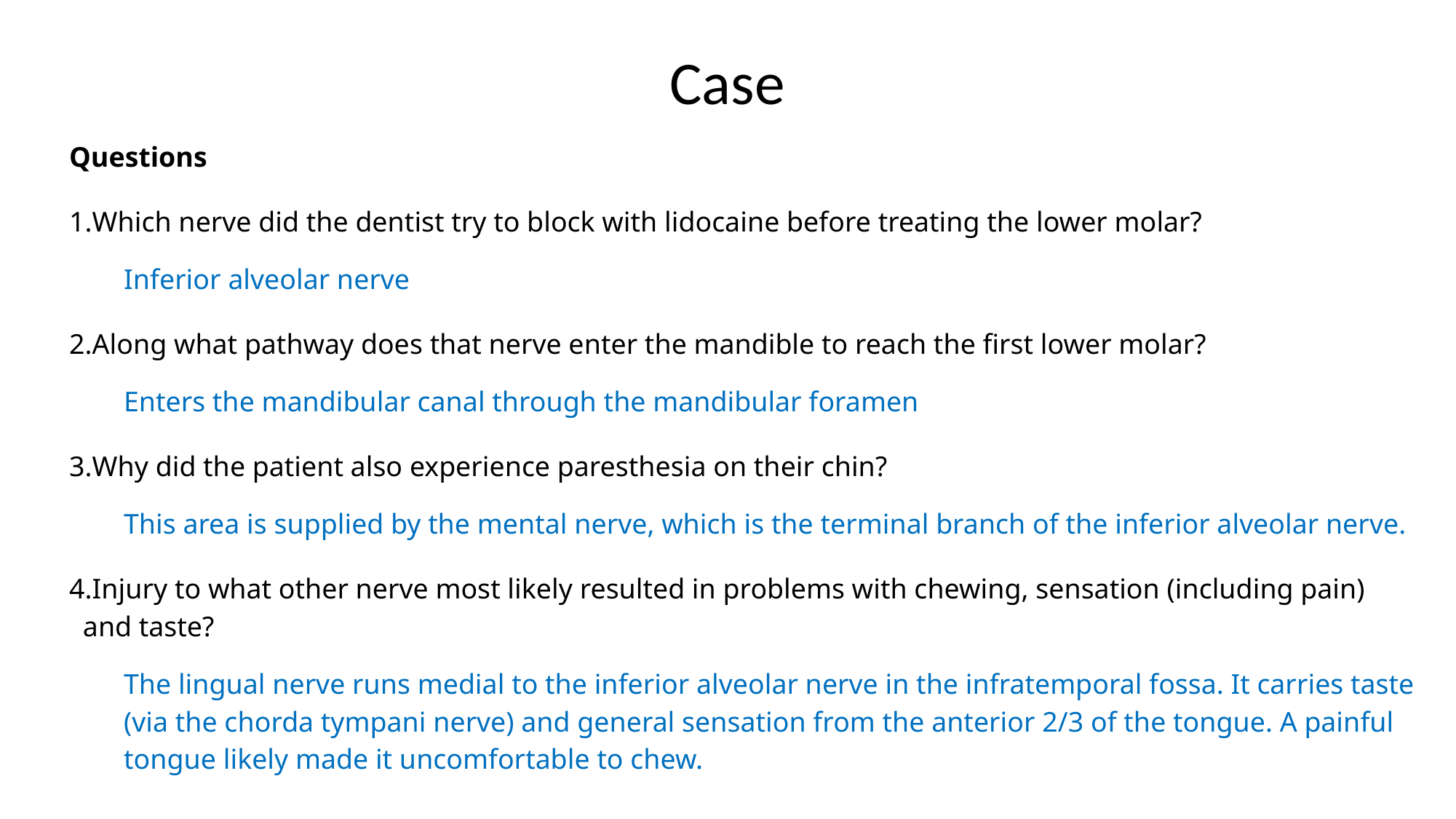

# Case
Questions
Which nerve did the dentist try to block with lidocaine before treating the lower molar?
Inferior alveolar nerve
Along what pathway does that nerve enter the mandible to reach the first lower molar?
Enters the mandibular canal through the mandibular foramen
Why did the patient also experience paresthesia on their chin?
This area is supplied by the mental nerve, which is the terminal branch of the inferior alveolar nerve.
Injury to what other nerve most likely resulted in problems with chewing, sensation (including pain) and taste?
The lingual nerve runs medial to the inferior alveolar nerve in the infratemporal fossa. It carries taste (via the chorda tympani nerve) and general sensation from the anterior 2/3 of the tongue. A painful tongue likely made it uncomfortable to chew.

## Slide 21
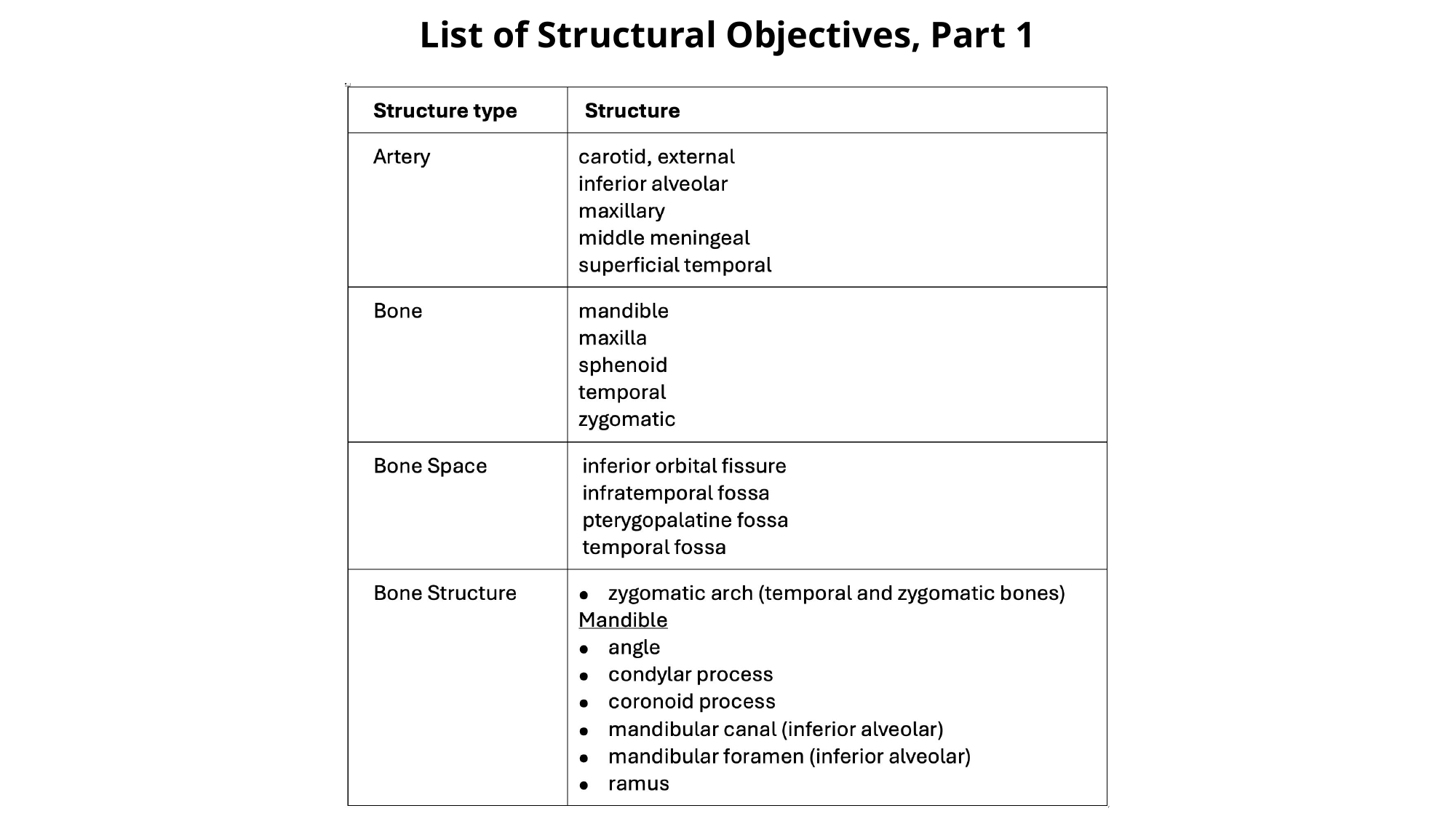

# List of Structural Objectives, Part 1

## Slide 22
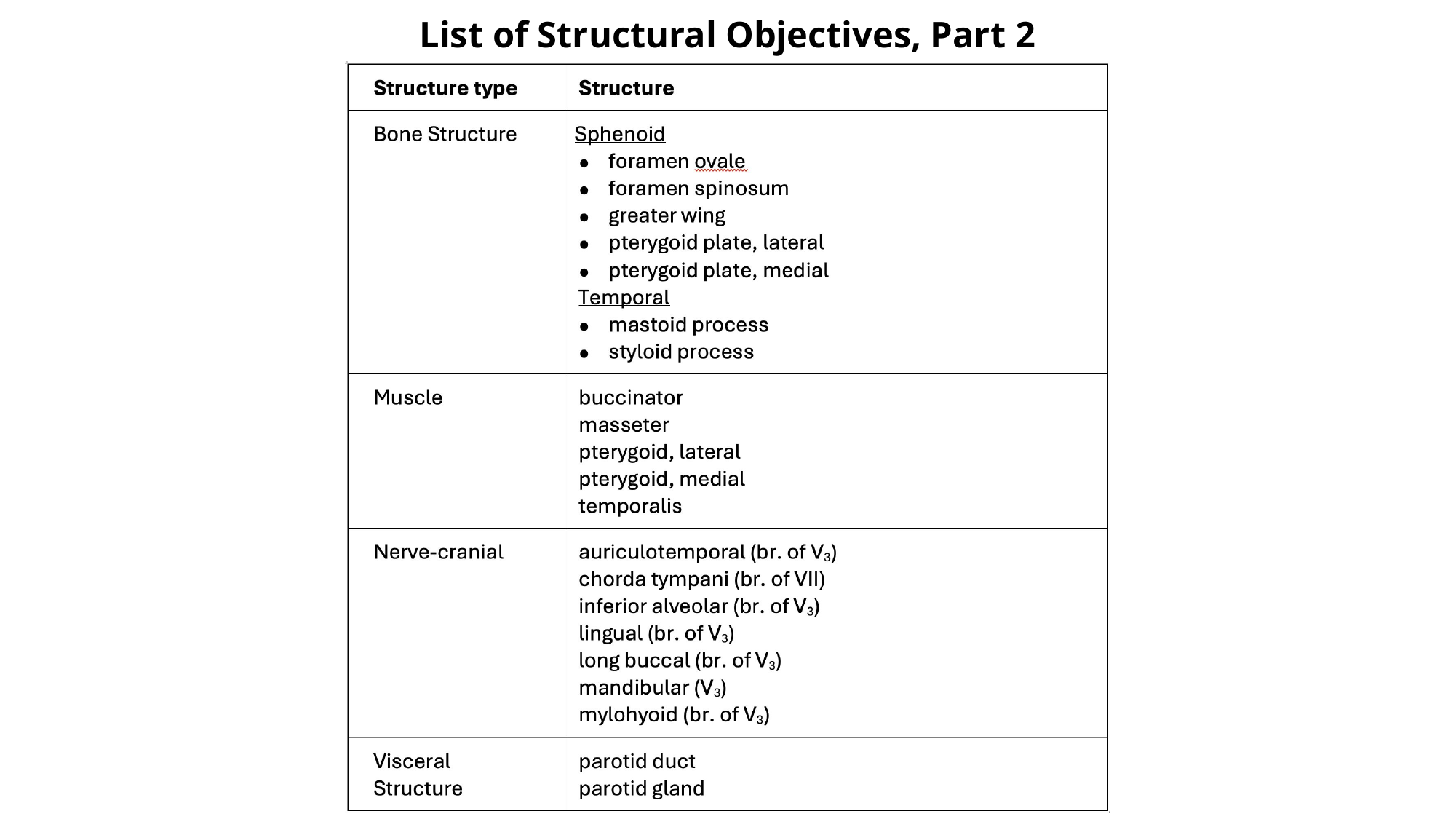

# List of Structural Objectives, Part 2
