# Supplementary material for: Using the Situated Learning-Guided Educational Framework to Teach Anatomy of the Infratemporal Fossa and Retromandibular Region
Source: MedEdPORTAL. 2025 Oct 3;21:11550. doi: 10.15766/mep_2374-8265.11550 (PMC12491565; doi:10.15766/mep_2374-8265.11550)
Supplement: Supplementary file 1 — Infratemporal Fossa Module (Instructor).pptxRetromandibular Region Module (Instructor).pptxInfratemporal Fossa Module (Student).pptxRetromandibular Region Module (Student).pptxPretest.docxPosttest.docxSurvey - Infratemporal Fossa.docxSurvey - Retromandibular Region.docx [file mep_2374-8265.11550-s001.zip › E. Pretest.docx]

**Pre-test – Retromandibular Region & Infratemporal Fossa**

*Tagged structures and correct answers are shown in grey. If more than one answer is accepted as correct, the acceptable options are separated by “/”.*

Instructions:

- You will have a total of 10 minutes to complete this test.
- For Questions 1-6, fill in the blanks with the name of structures tagged by a colored pin on the prosection.
- For Questions 7-10, read the clinical vignette and provide a free-text answer for each question.

1. Mylohyoid nerve/nerve to mylohyoid
2. Buccinator muscle
3. Maxillary artery
4. Parotid duct
5. Superior thyroid artery
6. Retromandibular vein

**(Q7-8)** You are performing a neurological exam on patient JK. JK is able to close his eyes tightly so that you cannot open them.

1. Name the muscle involved above: Orbicularis oculi
2. Name the innervation of muscle: Facial nerve (CN VII)
3. You are caring for a teenage boy that was involved in a fight at school. He was punched in the side of his face and now has a decreased ability to taste. What structure do you think was damaged? Chorda tympani
4. The muscles of mastication are innervated by what structure? Trigeminal nerve/CN V/Mandibular division of trigeminal nerve/Mandibular branch of trigeminal nerve/CN V_3_
