# Supplementary material for: Using the Situated Learning-Guided Educational Framework to Teach Anatomy of the Infratemporal Fossa and Retromandibular Region
Source: MedEdPORTAL. 2025 Oct 3;21:11550. doi: 10.15766/mep_2374-8265.11550 (PMC12491565; doi:10.15766/mep_2374-8265.11550)
Supplement: Supplementary file 1 — Infratemporal Fossa Module (Instructor).pptxRetromandibular Region Module (Instructor).pptxInfratemporal Fossa Module (Student).pptxRetromandibular Region Module (Student).pptxPretest.docxPosttest.docxSurvey - Infratemporal Fossa.docxSurvey - Retromandibular Region.docx [file mep_2374-8265.11550-s001.zip › F. Posttest.docx]

**Post-test – Retromandibular Region & Infratemporal Fossa**

*Tagged structures and correct answers are shown in grey. If more than one answer is accepted as correct, the acceptable options are separated by “/”.*

Instructions:

- You will have a total of 10 minutes to complete this test.
- For Questions 1-6, fill in the blanks with the name of structures tagged by a colored pin on the prosection.
- For Questions 7-10, read the clinical vignette and provide a free-text answer for each question.

1. Medial pterygoid muscle
2. Auriculotemporal nerve
3. Inferior alveolar artery
4. Hypoglossal nerve
5. External carotid artery
6. Mylohyoid nerve/nerve to mylohyoid
7. A patient was taken to surgery for a broken mandible. You see them a couple days after, and they report that they are now having some decreased sensation to their jaw. What structure would you be concerned for injury? Inferior alveolar nerve
8. A couple months have passed, and the same patient has returned to the clinic. They report that every time they eat, the skin around their ear turns red and is sweating as well. What condition does the patient have? Frey’s syndrome/gustatory sweating
9. You are reviewing the anatomy for a thyroidectomy on your surgery rotation. What artery supplies the thyroid gland from the external carotid artery? Superior thyroid artery
10. You are in the operating room assisting with a “temporal artery biopsy” procedure on a patient. You identify the superficial temporal artery correctly, but your Attending asks you to identify **the nerve** that courses with it. Auriculotemporal nerve
