# Supplementary material for: Using the Situated Learning-Guided Educational Framework to Teach Anatomy of the Infratemporal Fossa and Retromandibular Region
Source: MedEdPORTAL. 2025 Oct 3;21:11550. doi: 10.15766/mep_2374-8265.11550 (PMC12491565; doi:10.15766/mep_2374-8265.11550)
Supplement: Supplementary file 1 — Infratemporal Fossa Module (Instructor).pptxRetromandibular Region Module (Instructor).pptxInfratemporal Fossa Module (Student).pptxRetromandibular Region Module (Student).pptxPretest.docxPosttest.docxSurvey - Infratemporal Fossa.docxSurvey - Retromandibular Region.docx [file mep_2374-8265.11550-s001.zip › H. Survey - Retromandibular Region.docx]

**RETROMANDIBULAR REGION: SURVERY**

Q1. Overall, I think the guided learning session (module + cadaver prosection) is an excellent tool to study the retromandibular region.

- 1: Strongly Disagree
- 2: Disagree
- 3: Neither agree nor disagree
- 4: Agree
- 5: Strongly Agree

Q2. After this learning session, I can identify all required structures of the retromandibular region.

- 1: Strongly Disagree
- 2: Disagree
- 3: Neither agree nor disagree
- 4: Agree
- 5: Strongly Agree

Q3. The amount of time allotted for the learning session is appropriate.

- 1: Strongly Disagree
- 2: Disagree
- 3: Neither agree nor disagree
- 4: Agree
- 5: Strongly Agree

Q4. The use of cadaveric prosections is better for teaching the anatomy of the retromandibular region than student cadaveric dissection.

- 1: Strongly Disagree
- 2: Disagree
- 3: Neither agree nor disagree
- 4: Agree
- 5: Strongly Agree

Q5. I would prefer to participate in a guided learning session (module + cadaver prosection) than dissect the retromandibular region.

- 1: Strongly Disagree
- 2: Disagree
- 3: Neither agree nor disagree
- 4: Agree
- 5: Strongly Agree

Q6. I participated in the dissection of the retromandibular region on 09/11/2023:

1: Yes

2: No

**If you answered Yes above, answer Q7. If No, proceed to Q8.**

Q7. Please rank on a scale of 0 – 5 an estimate of your average stress experienced during cadaveric dissection of the retromandibular region.

- 1: No Stress
- 2: Mild Stress
- 3: Moderate Stress
- 4: Much Stress
- 5: Extreme Stress

Q8. Please rank on a scale of 0 – 5 an estimate of your average stress experienced during the guided learning session (module + cadaveric dissection) of the retromandibular region.

- 1: No Stress
- 2: Mild Stress
- 3: Moderate Stress
- 4: Much Stress
- 5: Extreme Stress

Q9. How could the workshop improve?

Q10. Please add any other feedback you would like to give the developers.

THANK YOU.
