# Supplementary material for: Using the Situated Learning-Guided Educational Framework to Teach Anatomy of the Infratemporal Fossa and Retromandibular Region
Source: MedEdPORTAL. 2025 Oct 3;21:11550. doi: 10.15766/mep_2374-8265.11550 (PMC12491565; doi:10.15766/mep_2374-8265.11550)
Supplement: Supplementary file 1 — Infratemporal Fossa Module (Instructor).pptxRetromandibular Region Module (Instructor).pptxInfratemporal Fossa Module (Student).pptxRetromandibular Region Module (Student).pptxPretest.docxPosttest.docxSurvey - Infratemporal Fossa.docxSurvey - Retromandibular Region.docx [file mep_2374-8265.11550-s001.zip › G. Survey - Infratemporal Fossa.docx]

**Infratemporal Fossa: Survey**

Q1. Record below the main group and focus group to which you were assigned:

Q2. I participated in the dissection of the infratemporal fossa (9/11/2023).

- Yes
- No

Q3. How adequate was the amount of time for the guided workshop?

- 1: needed more time
- 2: just the right amount of time
- 3: needed less time

**If you answered “Yes” for Q2, answer question 4. If you answered “No” for Q2 skip to Q5.**

Q4. The workshop (module + prosection) was an effective tool that helped me learn the anatomy of the infratemporal fossa.

- 1: Strongly disagree
- 2: Disagree
- 3: Neither agree nor disagree
- 4: Agree
- 5: Strongly agree

Q5. The workshop (module + prosection) was an effective tool that helped me revise the anatomy of the infratemporal fossa.

- 1: Strongly disagree
- 2: Disagree
- 3: Neither agree nor disagree
- 4: Agree
- 5: Strongly agree

Q6. I would prefer to learn the anatomy of the infratemporal fossa by attending the guided workshop (module + prosection) than by dissecting this region.

- 1: Strongly disagree
- 2: Disagree
- 3. Neutral
- 4: Agree
- 5: Strongly agree

Q7. The cadaveric dissection of the infratemporal fossa was a stressful experience.

- 1: Strongly disagree
- 2: Disagree
- 3. Neutral
- 4: Agree
- 5: Strongly agree

Q8. The guided workshop (module + prosection) of the infratemporal fossa was a stressful experience.

- 1: Strongly disagree
- 2: Disagree
- 3. Neutral
- 4: Agree
- 5: Strongly agree

Q9: How could this workshop improve?

Q10: Please provide any other feedback or comments.
