# Supplementary material for: Using the Situated Learning-Guided Educational Framework to Teach Anatomy of the Infratemporal Fossa and Retromandibular Region
Source: MedEdPORTAL. 2025 Oct 3;21:11550. doi: 10.15766/mep_2374-8265.11550 (PMC12491565; doi:10.15766/mep_2374-8265.11550)
Supplement: Supplementary file 1 — Infratemporal Fossa Module (Instructor).pptxRetromandibular Region Module (Instructor).pptxInfratemporal Fossa Module (Student).pptxRetromandibular Region Module (Student).pptxPretest.docxPosttest.docxSurvey - Infratemporal Fossa.docxSurvey - Retromandibular Region.docx [file mep_2374-8265.11550-s001.zip › _Educational Summary Report_11550.pdf]

MedEdPORTAL® The AAMC Journal of Teaching and Learning Resources

Original Publication

Open Access

Using the Situated Learning-Guided Educational Framework to Teach Anatomy of the Infratemporal Fossa and Retromandibular Region

Kevin T. Mutore, MD[†], Nina Stephen, MD[†], Kendra Stephen, MD[†], Alisa Winkler, PhD, Janine Prange-Kiel, PhD*

*Corresponding author: janine.prange-kiel@utsouthwestern.edu

[†]Co-primary author

Abstract

Introduction: Head and neck anatomy is a complex topic for students to learn and educators to teach. The current, most common pedagogical approach is student-conducted dissection. However, dissection of this region can be difficult, may be stressful, and might not optimally impart the foundational knowledge necessary to understand clinical scenarios. To address these challenges, we developed an instructor-guided, situated learning workshop using prosections of the infratemporal fossa and retromandibular region. This workshop aimed to reduce learner stress and improve their knowledge of these anatomic areas. **Methods:** Twenty-four first-year medical students, who were enrolled in a dissection-based anatomy course, participated in the 40-minute, instructor-facilitated workshop. Students completed a survey and a pre/postworkshop test to gauge perceived stress levels and knowledge retention. Mean values of the pre/postworshop tests were compared using a paired two-sample *t* test. **Results:** Workshop participation significantly improved student learning of head and neck anatomic subregions. Postworkshop knowledge scores were significantly higher than preworkshop scores (mean of 8.75 [95% CI, 8.12-9.38] vs. 4.79 [95% CI, 3.68-5.90]; $p < .001$). Additionally, in 75% of the survey answers, the learners reported mild or no stress during the workshop (36 of 48 total replies). **Discussion:** Our findings suggest that a prosection-centered, instructor-guided workshop is an effective and low-stress approach for teaching students complex head and neck anatomy. This workshop can serve as an alternative to, or enhancement of, student-conducted dissection of the area. Although originally designed for medical students, the approach may also benefit learners in other health professions.

Keywords
Gross Anatomy, Anatomy, Retromandibular Region, Infratemporal Fossa, Otolaryngology, Primary Care, Laboratory Education

Educational Objectives

By the end of this activity, learners will be able to:

1. Identify anatomic structures in the infratemporal fossa and retromandibular region of the head on prosections.
2. Describe the spatial relationships among structures within these locations of the head.
3. Apply their knowledge of the anatomy of these areas to interpret the anatomic basis for clinical presentations.

Introduction

Head and neck anatomy is uniquely challenging both for medical students to study and for anatomy educators to teach.[1] This is particularly true for the infratemporal fossa and retromandibular region, which contain multiple small, intricate structures within a spatially constrained section of the body. These challenges make it difficult for learners to dissect and identify important structures and their spatial relationships.[2] Dissection in this region often requires the removal of bony structures and other dense tissues to improve visualization, adding a layer of complexity and stress. A less stressful, more efficient learning experience is needed to enhance understanding and knowledge retention of these areas. Therefore, this workshop is based on situated-learning theory (SLT) and utilizes prosections to provide an effective and engaging learning experience in a low-stress environment.

While dissection of body donors has traditionally been fundamental for learning anatomy, many institutions are increasingly using prosections (i.e., parts of the body dissected by an expert) in their curricula.[3] Prosections significantly reduce the time required for students to learn complex anatomy while being more cost effective and without sacrificing educational

Citation:
Mutore KT, Stephen N, Stephen K, Winkler A, Prange-Kiel J. Using the situated learning-guided educational framework to teach anatomy of the infratemporal fossa and retromandibular region. *MedEdPORTAL*. 2025;21:11550. https://doi.org/10.15766/mep_2374-8265.11550

Copyright © 2025 Mutore et al. This is an open-access publication distributed under the terms of the Creative Commons Attribution license.

outcomes.[4] Furthermore, prosections are useful when delicate structures and limited space require difficult dissections that students struggle to perform adequately.[5] Thus, prosections provide clear views of complex structures and allow students to observe the normal anatomic variations in different specimens.[6] However, dissection offers students valuable experiential learning by allowing them to explore anatomic relationships themselves.[7] Implementing prosections instead of student-conducted dissection eliminates this experiential learning component. This gap can be reduced by incorporating SLT into head and neck anatomy workshops.

Grounded in constructivist theory, SLT was introduced in the 1990s and describes learning that is enhanced through the use of real-world contexts, collaborative environments, and engagement with communities of practice.[8] This theory has gained prominence as a leading model in health care professions education.[9,10] SLT also encourages educators to create interactive experiences that facilitate deeper comprehension of complex anatomic structures through hands-on learning and peer collaboration. Based on these principles, we designed our workshop to preserve the benefits of exploratory learning, with students interacting in small groups and lessons led by an instructor using anatomic specimens. This allowed us to leverage SLT, while preserving the flexibility associated with prosections.

Although alternative pedagogical approaches to student-led dissection for teaching anatomy have been explored, few specifically focus on detailed head or neck areas such as the infratemporal fossa or retromandibular region.[11,12] For example, existing *MedEdPORTAL* articles primarily offer general anatomy lessons with some modules providing specific instruction on blood supply, lymphatics, or cranial nerves.[13-15] Given the potential benefits of utilizing prosections, we developed and implemented this prosection-based, SLT-informed, active-learning workshop for teaching difficult head and neck anatomy to medical students.

Methods

We developed a supplemental workshop for first-year medical students while they are enrolled in the human anatomy course. We piloted this workshop in a course (Human Structure) that included instruction in anatomy, embryology, and introductory radiology. Prior to this workshop, students had dissected the back, upper extremity, neck, and face, and also had studied prosections of these regions. Our class included 235 students and employed an alternating dissection approach. The class was divided into two groups, with 3–4 students from each group assigned to the same dissection table. These groups performed dissections on an alternating schedule, but the students were responsible for all course content. Thus, only about half of the students in our course (and enrolled in our workshop) had previously dissected the infratemporal fossa and retromandibular region. The students had not been formally tested on the material prior to the workshop. The University of Texas Southwestern (UTSW) Institutional Review Board reviewed this project and deemed it to be exempt prior to its execution (IRB No. STU-2023-0916). The workshop was conducted in the Fall of 2022.

Student Recruitment

We recruited learners via an email outlining the workshop's aims and offered them the opportunity to sign up for a 40-minute workshop. The number of attendees was capped at 30 because we had limited resources (number of prosections and available facilitators). While the learners who participated in the workshop had the opportunity to experience an innovative approach to the material, all students in the class had ample opportunity to be exposed to the material. For example, students can access the laboratory during off-hours, and teaching assistants and faculty members are available during help sessions.

Facilitators

Two of the authors (Nina Stephen and Kendra Stephen) facilitated and developed this curriculum during their third year of medical school. The workshop was also conducted during this period. In developing the curriculum, they applied their proficiency in head and neck anatomy gained through their experience as anatomy teaching assistants and lab tutors following completion of the Human Structure course during their first year of medical school. By using near-peer instructors, a more accessible, mentorship-based learning environment was intentionally created for the curriculum. This environment should improve the adoption of the SLT curriculum, as it allows for learning through guided participation. This apprenticeship-style approach has been shown to improve knowledge retention.[9] However, it would also be possible for anatomy faculty to lead this workshop.

Preparation of Materials

Prosections: We used six prosections that were dissected prior to the workshop — three infratemporal fossae and three retromandibular regions. We dissected the prosections according to our institution's in-house dissection guide to ensure that a defined set of structures was exposed. However, the methodology for the dissections is adaptable to other institution's instructions. For both regions, we used one prosection each for the preworkshop and postworkshop tests, and one for the instructor-guided active learning sessions.

Copyright © 2025 Mutore et al. This is an open-access publication distributed under the terms of the Creative Commons Attribution license.

Learning module: We developed learning modules for facilitators (instructor copy, Appendices A and B) and students (student copy, Appendices C and D) for both prosection sessions. The instructor modules discussed educational objectives and provided a step-by-step description of how to conduct each session. The documents contained didactic information about the contents of each region. During the learning sessions, the facilitators demonstrated the contents of each region as described in the learning modules. In our pilot, these documents also featured labeled images of prosections showing relevant structures. In the student copy, however, some images included unlabeled structures, allowing the learners to test themselves at specific points during the active learning session. This was done deliberately, as teaching through an SLT lens emphasizes that learning is best done through active participation. By employing learning through action, students were able to obtain feedback on their knowledge base. Although images of our donors are not included in Appendices A–D, we recommend using images that are either owned by the user's institution or sourced from other resources, some of which are suggested in the instructor copies of the modules.

Preworkshop and postworkshop tests: Preworkshop and postworkshop tests (Appendices E and F) covered the list of structural objectives (testable structures) that we provided to the students in their learning module. Each test lasted 10 minutes and included 10 questions, asking students to identify six tagged structures on the prosections and to provide short answers to four questions. These questions were developed by authors Nina Stephen and Kendra Stephen, and their degree of difficulty was comparable to that of questions used in the course exams. The preworkshop and postworkshop test scores are reported as number of questions correctly answered.

Surveys: Directly after the workshop, learners completed surveys based on 5-point Likert scales to evaluate their perception of the prosection-centered learning sessions and to assess their stress levels during the sessions. The surveys ended with a section for the students to provide unstructured feedback on their workshop experience (Appendices G and H). The surveys are different for the infratemporal fossa and retromandibular region because we originally developed these modules as separate UTSW Scholarly Activity projects. The workshop was held 10 days after the student-led dissection of the relevant areas. Therefore, students who participated in both the dissection and the workshop were well positioned to compare their stress levels between the two approaches.

Workshop Session

We conducted the workshop in three cohorts (morning, afternoon, and evening) 10 days after the students dissected the infratemporal fossa and retromandibular region. Students were randomly assigned into two groups—Group A or Group B—each comprising 3–5 students. To de-identify test and survey results, each student received a random number, which was used as their identification for the preworkshop and postworkshop tests.

Prior to the workshop, we as facilitators briefly explained the format for the session. The workshop began with the preworkshop test, after which the students received paper copies of the student learning modules (Appendices C and D) for reference during the learning sessions.

Group A started with the retromandibular region, while Group B began with the infratemporal fossa. Importantly, one facilitator worked with both groups for the retromandibular region and the other worked with both groups for the infratemporal fossa. Each facilitator utilized the respective learning module (Appendix A or B) and prosection. Groups participated in a 20-minute instructor-guided session before switching to the other learning session.

After the sessions, the students completed the postworkshop test and were encouraged to complete the survey.

Statistical Analysis

Mean scores on the preworkshop and postworkshop tests and surveys were calculated and the scores for the preworkshop and postworkshop tests were compared in Microsoft Excel using a paired two-sample t test. Significance was set at $p < .05$.

Results

Twenty-four students participated. Thirteen students (54%) had performed the student-conducted dissections prior to the workshop, while the remaining 11 students (46%) had not previously performed the student-conducted dissections of the study regions.

Preworkshop and Postworkshop Tests

We evaluated student learning among all participants ($N = 24$) using a preworkshop test to assess knowledge prior to the workshop and a postworkshop test to measure knowledge acquisition after completing the workshop. Overall, the postworkshop knowledge scores were significantly higher than the preworkshop knowledge scores. Mean (*SD*) knowledge scores were 4.79 (2.62) points (95% CI, 3.68-5.90) preworkshop versus 8.75 (1.48) points (95% CI, 8.12-9.38) postworkshop

Copyright © 2025 Mutore et al. This is an open-access publication distributed under the terms of the Creative Commons Attribution license.

($p < .001$). Improvement in postworkshop test scores was comparable between students who had previously dissected these regions and students who had only self-studied the material.

Survey

We analyzed survey results from students who completed both the student-led dissections and the workshop and from those who participated in the workshop only. Students rated their perceived stress levels during these experiences using 5-point-Likert scales (for infratemporal fossa [Appendix G]: 1 = *strongly disagree*, 5 = *strongly agree*; for retromandibular region [Appendix H], 1 = *no stress*, 5 = *extreme stress*). As shown in Figures 1 and 2, the perceived stress levels were higher during dissection compared to the prosection-centered learning sessions. For the infratemporal fossa, students' mean (*SD*) stress level rating was 3.23 (1.17) for the dissection compared to 2.07 (1.55) for the respective workshop ($n = 13$). Similarly, for the retromandibular region, students' mean (SD) stress level rating was 2.50 (0.70) for the dissection but only 1.70 (1.06) for the workshop ($n = 10$). Overall, students who attended only the learning sessions also reported a low stress level during the learning sessions (Figures 3 and 4). For the workshop on the infratemporal fossa, students' mean (*SD*) stress level rating was 1.92 (1.44; $n = 13$), and for the workshop on the retromandibular region, students' mean (*SD*) stress level rating was 1.75 (1.06; $n = 12$).

Nineteen learners provided written feedback on their experience. Eight students requested more time, two students wanted more quizzes, and one student asked for additional clinical correlates during the learning sessions. Three students felt that no improvements were necessary. One student felt pressured during the workshop, and another noted that, although the workshop served as a good review, it was unsuitable for first-time learners. Additionally, two students recommended including a review at the end of the learning sessions, and one suggested smaller group sizes.

Below are some students' comments regarding their workshop experience:

- "The interactive nature of this workshop was really helpful for learning the structures. The prosections were cleanly dissected and easy to learn from."
- "We should do this for all dissections."
- "Just perfect but needs more time."
- "This was extremely helpful and is a better job of teaching the material. I hope these sessions can be implemented as part of the regular curriculum."
- "Please implement prosections. They are a much better use of our time."

Discussion

Head and neck anatomy can be difficult to teach and learn. We successfully deployed an instructor-guided workshop that enhanced learning outcomes for first-year medical students. This prosection-centered workshop significantly improved the students' understanding of these regions, which are highly relevant to students in preclinical training and are not covered by current literature. The workshop also received positive student feedback and allowed students to learn in a low-stress environment. This outcome is important, as such low-stress learning environments can enhance cognitive function and retention of information.[16] The positive survey responses regarding the workshop format suggest that the students found this method engaging and manageable, potentially contributing to their improved performance. Furthermore, it is important to

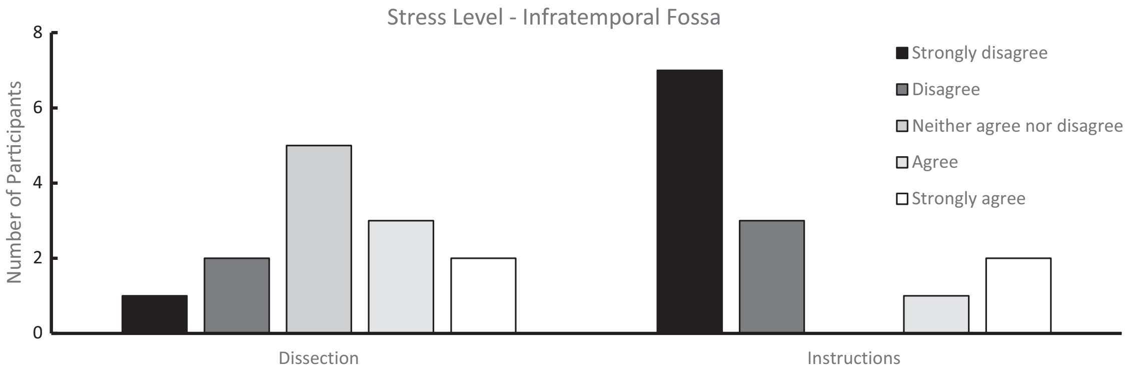

Figure 1. Survey results on stress level for learners who attended both student-conducted dissection (left) and the prosection-centered learning session (right) for the infratemporal fossa ($n = 13$). Ratings were on a 5-point Likert scale (1 = *strongly disagree*, 5 = *strongly agree*) in response to the statement: "The cadaveric dissection/guided workshop (learning module and prosection) was a stressful experience."

Copyright © 2025 Mutore et al. This is an open-access publication distributed under the terms of the Creative Commons Attribution license.

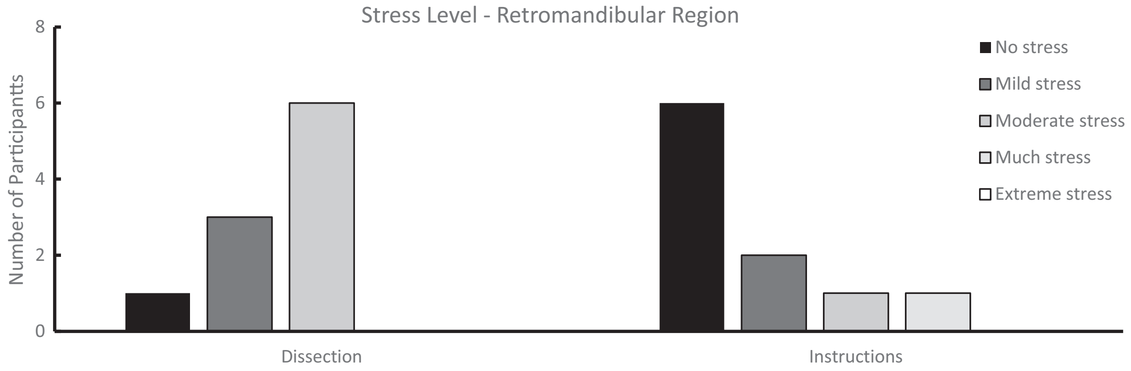

Figure 2. Survey results on stress level for learners who attended both student-conducted dissection (left) and the prosection-centered learning session (right) for the retromandibular region (n = 10). Ratings were on a 5-point Likert scale (1 = *no stress*, 5 = *extreme stress*) in response to the statement: "Rank … your average stress experienced during cadaveric dissection/guided learning session (learning module and prosection) of the retromandibular region".

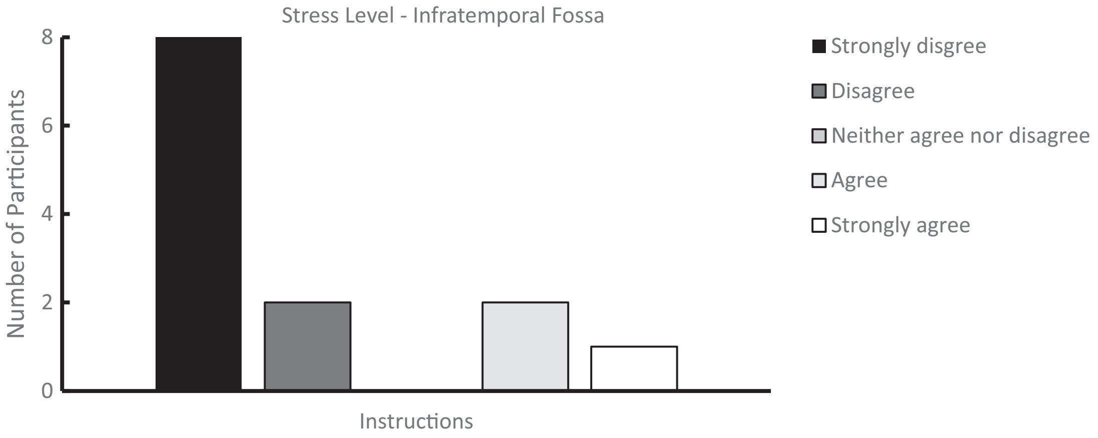

Figure 3. Survey results on stress level for learners who attended only the prosection-centered learning session for the infratemporal fossa (n = 13). Ratings were on a 5-point Likert scale (1 = *strongly disagree*, 5 = *strongly agree*) in response to the statement: "The guided workshop (learning module and prosection) was a stressful experience."

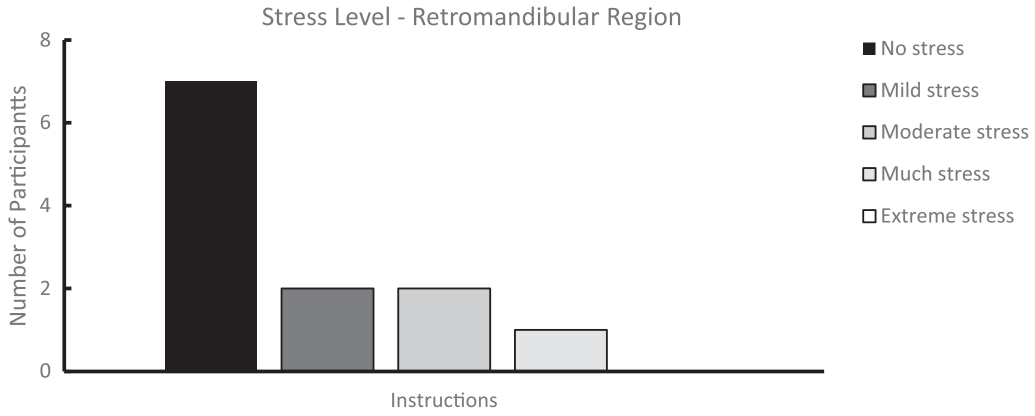

Figure 4. Survey results on stress level for learners who attended only the prosection-centered learning session for the retromandibular region (n = 12). Ratings were on a 5-point Likert scale (1 = *no stress*, 5 = *extreme stress*) in response to the statement: "Rank … your average stress experienced during guided learning session (learning module and prosection) of the retromandibular region."

Copyright © 2025 Mutore et al. This is an open-access publication distributed under the terms of the Creative Commons Attribution license.

note that the use of high-quality prosections will likely increase the reproducibility of these results.

This workshop also utilized the principles of SLT, which emphasizes collaborative, real-life context-based learning. To our knowledge, this is the first published anatomy instruction that uses SLT to teach the infratemporal fossa and retromandibular region. The workshop was led by medical student teaching assistants who were trained to deliver this material, demonstrating that this format empowers experienced students to facilitate sessions for their near peers. Furthermore, the small number of students per group allows for cooperation and hands-on guided exploration of the prosections. In addition, anatomic specimens were utilized in the workshop, which allows students to engage and learn in a context that mirrors the cases that they will encounter during their clinical years. We also encouraged social interaction, a tenet of SLT, to prompt students to talk through what they saw, question each other, and jointly problem-solve. This created an environment where students could acquire knowledge and contribute to their learning community.

Although knowledge test scores significantly improved postworkshop in this cohort, several limitations must be acknowledged. First, the small sample size may limit the generalizability of the findings. Second, given the short duration of this project, we were unable to assess the long-term retention of knowledge gained through our workshop. Furthermore, while the preworkshop and postworkshop tests provided some quantitative information about the efficacy of this workshop, we could not capture the students' ability to apply this knowledge practically. Finally, the voluntary nature of the workshop may have resulted in some self-selection bias, as it is possible that students who perceived themselves as struggling participated to gain additional anatomy instruction. If indeed the participating students represented student populations with limited knowledge, the results may not be applicable to students with more extensive knowledge within the class.

The workshop was developed in response to feedback from first-year medical students, particularly those receiving additional tutoring through the institutional Student Academic Support Service, who expressed difficulty with dissecting and conceptualizing these regions. As anatomy educators, we continuously move the curriculum toward more case-based learning. Therefore, this project was considered to be a pilot test to assess whether difficult, time-consuming dissections could successfully be replaced with prosection-based sessions led by advanced medical students. In 2025, we introduced a modified version of the workshop in the first-semester Health Profession Anatomy course. Thirty-minute–long interactive sessions, led by medical student teaching assistants using prosections, replaced student dissection of the retromandibular and infratemporal regions. These small-group sessions focused on module-relevant structures, including skull anatomy. The teaching assistant–led format was well received, with students benefiting from clear views of complex structures. Faculty members and teaching assistants found the approach to be effective, consistent with the outcomes from the initial pilot.

In addition to the tested approach, the workshop could also be used in prosection-based courses or as a stand-alone teaching tool for precourse preparation. This type of preparation not only might make the lab experience more effective but also could reduce the stress level for students. This material was developed for medical students but can be modified for a variety of educational levels and professional training programs, such as programs for dental students, surgical trainees, and physician assistants.

Moving forward, we plan to leverage student feedback to further enhance the learning experience. Our future efforts will include adding more clinical correlations and potentially expanding the prosection workshops to add additional anatomic regions. By including these changes, we aim to provide a more robust preclinical anatomy curriculum, ultimately improving student readiness for clinical practice.

Appendices

A. Infratemporal Fossa Module (Instructor).pptx
B. Retromandibular Region Module (Instructor).pptx
C. Infratemporal Fossa Module (Student).pptx
D. Retromandibular Region Module (Student).pptx
E. Pretest.docx
F. Posttest.docx
G. Survey - Infratemporal Fossa.docx
H. Survey - Retromandibular Region.docx

All appendices are peer reviewed as integral parts of the Original Publication.

Kevin T. Mutore, MD: Resident, Department of Surgery, University of Texas Southwestern Medical Center; ORCID: https://orcid.org/0000-0002-0667-9937

Copyright © 2025 Mutore et al. This is an open-access publication distributed under the terms of the Creative Commons Attribution license.

Nina Stephen, MD: Resident, Department of Head and Neck Surgery and Communication Sciences, Duke University School of Medicine

Kendra Stephen, MD: Resident, Department of Otolaryngology—Head and Neck Surgery, University of Mississippi Medical Center

Alisa Winkler, PhD: Associate Professor, Department of Surgery, University of Texas Southwestern Medical Center

Janine Prange-Kiel, PhD: Associate Professor, Department of Surgery, University of Texas Southwestern Medical Center; ORCID: https://orcid.org/0000-0003-4584-0188

Disclosures
None to report.

Funding/Support
None to report.

Ethical Approval
The University of Texas Southwestern reviewed this project.

References

1. Rehman U, Perwaiz I, Sarwar MS, Brennan PA. Are clinical medical students confident about their head and neck anatomy knowledge? *Br J Oral Maxillofac Surgy*. 2022;60(7):922-926. https://doi.org/10.1016/j.bjoms.2022.01.015
2. Cheung CC, Bridges SM, Tipoe GL. Why is anatomy difficult to learn? The implications for undergraduate medical curricula. *Anat Sci Educ.* 2021;14(6):752-763. https://doi.org/10.1002/ase.2071
3. Sugand K, Abrahams P, Khurana A. The anatomy of anatomy: a review for its modernization. *Anat Sci Educ.* 2010;3(2):83-93. https://doi.org/10.1002/ase.139
4. Koh ZJ, Yeow M, Srinivasan DK, Ng YK, Ponnamperuma GG, Chong CS. A randomized trial comparing cadaveric dissection and examination of prosections as applied surgical anatomy teaching pedagogies. *Anat Sci Educ.* 2023;16(1):57-70. https://doi.org/10.1002/ase.2166
5. Lopez EKN, Johnson JH, Cunningham EP, Warshaw J, Baker EW. A unique and effective method of anatomy education: Small-group learning with prosected plastinated specimens. *J Dent Educ.* 2022;86(4):425-436. https://doi.org/10.1002/jdd.12817
6. Topp KS. Prosection vs. dissection, the debate continues: rebuttal to Granger. *Anat Rec B New Anat.* 2004;281(1):12-14. https://doi.org/10.1002/ar.b.20037
7. Ghosh SK. Cadaveric dissection as an educational tool for anatomical sciences in the 21st century. *Anat Sci Educ.* 2017;10(3):286-299. https://doi.org/10.1002/ase.1649
8. O'Brien BC, Battista A. Situated learning theory in health professions education research: a scoping review. *Adv Health Sci Educ Theory Pract.* 2020;25(2):483-509. https://doi.org/10.1007/s10459-019-09900-w
9. Cruess RL, Cruess SR, Steinert Y. Medicine as a community of practice: implications for medical education. *Acad Med.* 2018;93(2):185-191. https://doi.org/10.1097/ACM.0000000000001826
10. Kaufman DM, Mann KV. Teaching and learning in medical education: how theory can inform practice. In: Swanwick T, ed. *Understanding Medical Education: Evidence, Theory and Practice*. The Association for the Study of Medical Education; 2010:16-36.
11. Estai M, Bunt S. Best teaching practices in anatomy education: A critical review. *Ann Anat.* 2016;208:151-157. https://doi.org/10.1016/j.aanat.2016.02.010
12. Wainman B, Aggarwal A, Birk SK, Gill JS, Hass KS, Fenesi B. Virtual dissection: an interactive anatomy learning tool. *Anat Sci Educ.* 2021;14(6):788-798. https://doi.org/10.1002/ase.2035
13. Klinkhachorn PS, Battin J, Lucas J, et al. Angiographic anatomy of the head and neck. *MedEdPORTAL.* 2015;11:10182. https://doi.org/10.15766/mep_2374-8265.10182
14. Reilly F, Allen E. Head and neck lymphatics. *MedEdPORTAL*. 2010;6:7858. https://doi.org/10.15766/mep_2374-8265.7858
15. Lack CM, Pena J, Grubb EXL, et al. Cranial nerve anatomy using a modular and multimodal radiologic approach. *MedEdPORTAL.* 2022;8:11261. https://doi.org/10.15766/mep_2374-8265.11261
16. Bangasser DA, Shors TJ. Critical brain circuits at the intersection between stress and learning. *Neurosci Biobehav Rev.* 2010;34(8):1223-1233. https://doi.org/10.1016/j.neubiorev.2010.02.002

Received: March 10, 2025
Accepted: August 19, 2025
Published: October 3, 2025

Copyright © 2025 Mutore et al. This is an open-access publication distributed under the terms of the Creative Commons Attribution license.
